# Ferrihydrite transformations in flooded paddy soils: rates, pathways, and product spatial distributions†

**DOI:** 10.1039/d2em00290f

**Published:** 2022-09-22

**Authors:** Andrew R. C. Grigg, Laurel K. ThomasArrigo, Katrin Schulz, Katherine A. Rothwell, Ralf Kaegi, Ruben Kretzschmar

**Affiliations:** Soil Chemistry Group, Institute of Biogeochemistry and Pollutant Dynamics, Department of Environmental Systems Science, ETH Zurich Universitätstrasse 16, CHN CH-8092 Zurich Switzerland andrew.grigg@usys.ethz.ch; Eawag, Swiss Federal Institute of Aquatic Science and Technology Überlandstrasse 133 CH-8600 Dübendorf Switzerland

## Abstract

Complex interactions between redox-driven element cycles in soils influence iron mineral transformation processes. The rates and pathways of iron mineral transformation processes have been studied intensely in model systems such as mixed suspensions, but transformation in complex heterogeneous porous media is not well understood. Here, mesh bags containing 0.5 g of ferrihydrite were incubated in five water-saturated paddy soils with contrasting microbial iron-reduction potential for up to twelve weeks. Using X-ray diffraction analysis, we show near-complete transformation of the ferrihydrite to lepidocrocite and goethite within six weeks in the soil with the highest iron(ii) release, and slower transformation with higher ratios of goethite to lepidocrocite in soils with lower iron(ii) release. In the least reduced soil, no mineral transformations were observed. In soils where ferrihydrite transformation occurred, the transformation rate was one to three orders of magnitude slower than transformation in comparable mixed-suspension studies. To interpret the spatial distribution of ferrihydrite and its transformation products, we developed a novel application of confocal micro-Raman spectroscopy in which we identified and mapped minerals on selected cross sections of mesh bag contents. After two weeks of flooded incubation, ferrihydrite was still abundant in the core of some mesh bags, and as a rim at the mineral–soil interface. The reacted outer core contained unevenly mixed ferrihydrite, goethite and lepidocrocite on the micrometre scale. The slower rate of transformation and uneven distribution of product minerals highlight the influence of biogeochemically complex matrices and diffusion processes on the transformation of minerals, and the importance of studying iron mineral transformation in environmental media.

## Abstract 2

The rate and pathway of ferrihydrite transformation in soil depends on the properties of the soil pore water and diffusion processes.

## Graphical abstract

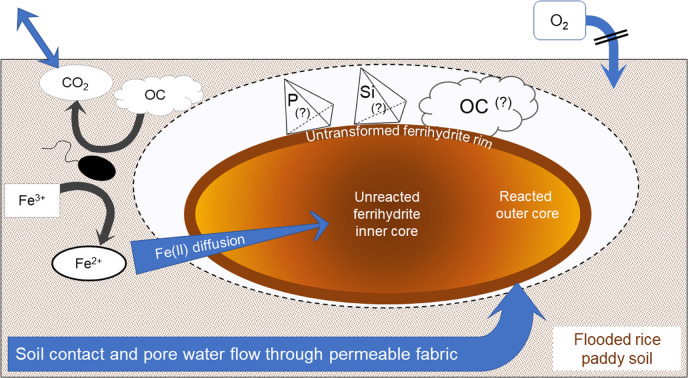

**Environmental significance**

In rice paddy soils, ferrihydrite plays an important role in the cycling of pollutants, nutrients and carbon, but those same compounds may also influence the rates and pathways of ferrihydrite transformation. We take a step towards understanding transformation of ferrihydrite in flooded soils by demonstrating that ferrihydrite transformation in soils may be slower than has been observed in mixed laboratory systems. The transformation rates and pathways are highly dependent on the geochemical composition of the soil, particularly the iron-reduction potential. Moreover, the spatial distributions of mineral transformation products indicate that diffusion limitation and competing crystallisation pathways can influence the outcomes of ferrihydrite transformation processes in porous media such as soils.

## Introduction

Soils are complex media that facilitate simultaneous interactions of numerous coupled biogeochemical processes. In flooded soils, oxygen is depleted during the oxidation of organic carbon (OC) by microbial respiration, and alternative redox couples sequentially become favourable, allowing diverse microbial communities to catalyse the cascade of energy through inter-meshed cycles of redox-active species.^1,2^ However, not all redox couples are directly tied to OC. Chemical species within the cycles of N, Mn, Fe, S and trace elements may form redox couples with one another, often including short-lived species that are difficult to detect.^1,3–5^ In flooded soils, the cycling of Fe has particularly strong links to other element cycles because Fe is abundant, reactive under moderately reducing conditions and exists in a variety of phases (dissolved, chelated, sorbed and structural Fe) with varying reduction potentials.^1,6–8^ Iron cycling interfaces with the redox cycles of many major and trace elements, such as C, N, Mn, S, As, Cr, Cu, U, Hg, Se and Sb,^1,2,7–11^ and additionally influences the fate of redox-inactive chemical species by sorption–desorption interactions and incorporation/substitution–dissolution processes.^1,10,12–16^ At the same time, Fe cycling is mediated by the availability and kinetics of microbially mediated redox couples^7,8^ and by the presence of natural organic matter (NOM), cations and anions.^17–24^ Therefore, Fe cycling both regulates, and is regulated by, the complex web of biogeochemical reactions in redox-dynamic soils.

Ferrihydrite (Fe_10_O_14_(OH)_2_) is an abundant redox-dynamic short-range-ordered Fe(iii) oxyhydroxide present in many soils, particularly those containing freshly oxidised Fe.^25,26^ Ferrihydrite contributes disproportionately to the available sites that facilitate sorption and desorption of trace elements in soils due to its large specific surface area (up to ∼500 m^2^ g^−1^ in natural samples^27,28^) and high density of reactive surface sites.^28,29^ However, ferrihydrite is thermodynamically unstable with respect to more crystalline iron (oxyhydr)oxides,^30^ and the dissolution, recrystallisation or transformation of ferrihydrite may mobilise incorporated or substituted trace elements or immobilise trace elements by incorporation and substitution in secondary phases.^16^ Under reducing conditions, ferrihydrite will be among the first Fe minerals to reductively dissolve, either biotically^31,32^ or abiotically,^33^ releasing Fe(ii) into solution.

In laboratory studies, Fe(ii) (0.1–0.7 w/w Fe(ii)-to-ferrihydrite ratio) at circumneutral pH (5–8) and room temperature has been shown to catalyse the transformation of ferrihydrite to more thermodynamically stable phases such as lepidocrocite (γ-FeOOH), goethite (α-FeOOH) or magnetite (Fe_3_O_4_) within days.^34–39^ Transformation to goethite and lepidocrocite is understood to proceed by sorption of Fe(ii) to mineral surfaces, followed by oxidation of the sorbed Fe and electron transfer to the mineral.^35,37,40,41^ The rates and products depend strongly on the ratio of Fe(ii) to ferrihydrite.^21,37,42–46^ Moreover, previous studies have measured effects on the rates and products of Fe(ii)-catalysed ferrihydrite transformation caused by diverse dissolved or sorbed metal ions,^20,23,47,48^ structurally incorporated cations,^9,17,19,23,49,50^ dissolved, sorbed or structurally incorporated inorganic anions,^19,21,22,51–54^ co-precipitated or sorbed organic compounds,^17,18,55–57^ cultivated bacteria,^56,58,59^ as well as varying pH,^34,37,60^ temperature,^36,51,60^ and ferrihydrite-to-solution ratio.^44^ These studies suggest that soil components may reduce electron flow from sorbed Fe(ii), for example, by competition with Fe(ii) for ferrihydrite surface sorption sites, or that soil components may be toxic to soil microbes, reducing the rate of mineral transformation. Nonetheless, ferrihydrite recrystallisation may occur when transformation is inhibited, for example by the presence of co-precipitated NOM^18,61,62^ or Si.^17,50,52,53^ Complex media may also change the products of transformation, by altering the interactions of Fe(ii) with the mineral surface and thereby influencing nucleation and growth of secondary mineral phases.^17,21,23,42,51,63^

Whereas much of the current understanding about ferrihydrite transformation is based on experiments in mixed suspensions, there is increasing interest in examining the effect of complex biogeochemical and physical interactions on mineral transformation using semi-permeable or permeable containers to observe mineral behaviour under environmental conditions. In one example, ferrihydrite from a wastewater treatment plant, mixed with glass balls, was aged in heavy-metal contaminated soils inside polyamide bags with pore size of 20 μm.^64^ More than 50% of the ferrihydrite transformed to goethite within the four-year span of the experiment in a process that removed contaminants from soil.^64^ In another study, synthetic jarosite and schwertmannite, in nylon mesh bags (1 μm pore size) with an empty perforated polypropylene housing, partially transformed to goethite within twelve months of exposure to acidic surface water and neutral anoxic sediments in an acid-sulfate soil environment.^65^ One study of redox-active minerals in rice paddies employed polyamide mesh bags (20 μm pore size) to react synthetic ferrihydrite, goethite and nontronite-rich natural clays in a quartz sand bed that was installed below the puddled layer of three Chinese paddy fields.^66^ Over twelve months, goethite remained untransformed, the crystal structure of nontronite was altered and ferrihydrite transformed into lepidocrocite, goethite and hematite, with relative abundance depending on the geochemical characteristics of the field.^66^ A study that exposed gel-embedded ferrihydrite and lepidocrocite to sediment pore water using DGT samplers found partial transformation to iron sulfide in four weeks.^67^ Sequential extraction techniques have also been used measure ferrihydrite transformation to crystalline products when mixed into wet paddy soils.^68^ These studies showed that Fe minerals exposed to flooded soils and sediments transformed over months or years,^64–68^ which is slower than has been observed in laboratory studies of Fe(ii)-catalysed transformation of ferrihydrite,^34–39^ jarosite at circumneutral pH,^17,69,70^ and schwertmannite at circumneutral pH.^71–74^

Recognising the nexus between Fe mineral transformations and the biogeochemical cycling of other ions and dissolved OC (DOC) in soils, we sought to investigate the transformation of ferrihydrite under the direct influence of flooded paddy soils. Firstly, we incubated ferrihydrite in soils with contrasting microbial Fe reduction potential to determine if soil pore water with varying Fe(ii) concentrations, or contact with soil, alters the ferrihydrite transformation rate and/or pathway with respect to simplified laboratory systems such as mixed-suspension experiments. Secondly, we investigated the impact of diffusion and competing recrystallisation pathways on the spatial relationship between ferrihydrite and its transformation products on millimetre to micrometre scales. To these ends, mesh bags, filled with synthetic two-line ferrihydrite, were placed in flooded soil microcosms for up to twelve weeks. X-ray diffraction (XRD) and wet chemical analysis revealed the effect of reducing conditions on soil chemical properties and ferrihydrite transformation inside the mesh bags. The spatial distribution of ferrihydrite or its transformation products was measured using a novel application of micro-Raman (μ-Raman) spectroscopy at ∼1 μm resolution across geochemical gradients within mesh bags. The results offer a new perspective on the ferrihydrite transformation processes that may occur in reducing soil environments.

## Methods

### Mineral synthesis

Two-line ferrihydrite was synthesised by neutralisation of a ferric iron solution similar to the method of Schwertmann and Cornell.^75^ Metallic Fe powder was dissolved in 2 M HCl and oxidised with excess H_2_O_2_. Traces of undissolved Fe(0) were removed with a magnetic stirrer bar and 2 μm cellulose filter. Ferrihydrite was precipitated by rapidly neutralising the Fe(iii) solution with 2 M KOH. The precipitates were washed through repeated cycles of centrifugation (3575*g*), decantation and re-suspension in ultra-pure water (Milli-Q, Millipore, 18.2 MΩ cm), until supernatant conductivity was <100 μS cm^−1^. The ferrihydrite was then shock frozen by drop-wise addition of the suspension into liquid N_2_, and subsequently freeze-dried. The dried ferrihydrite was gently crushed and homogenised using a mortar and pestle, then stored in an amber glass vial in a desiccator. The minerals were checked for structural impurities by XRD (details below), and elemental impurities by inductively coupled plasma optical emission spectroscopy (ICP-OES, 5100, Agilent Technologies, USA) after dissolution in 4 M HCl.

### Soils

Five rice paddy soils were chosen for their contrasting physico-chemical properties (pH, texture, C content, Fe content) and potential for Fe reductive dissolution under flooded conditions (measured under anoxic conditions in pre-tests, data not shown). Soils PT-T (topsoil from 0–15 cm depth) and PT-S (subsoil from ∼1 m depth), collected from the same paddy field in Pathum Thani province, Thailand, are examples of the acid sulfate clay soils that are widespread in the central provinces of Thailand.^76^ Soil UB is a sandy loam from a highly weathered paddy soil (0–15 cm depth) collected in Ubon Ratchathani province, Thailand. Soil CS is also a sandy loam, but with a much lower sand content than UB, collected at a rice cultivation research station (0–50 cm depth) in Hunan province, China.^77^ Soil BD is a silty clay loam paddy soil (0–10 cm depth) from Bangladesh.^78,79^ Details about the soils are provided in Tables S1 and S2.† All soils were dried and sieved to 2 mm. Elemental composition of bulk soils was measured by XRF (XEPOS, Spectro, Germany). Soil C content was measured by combustion on milled soils (vario MAX cube, Elementar, Germany).

### Microcosm experiment

Porous mesh bags were used to contain ferrihydrite and mineral transformation products during the microcosm experiments. The mesh bags were made with approximate internal dimension of 30 mm × 12 mm × 3 mm using polyethylene terephthalate (PETE) filter fabric with nominal pore size of 51 μm (yarn diameter 38 μm; SEFAR, Switzerland). This pore size was chosen to allow free exchange of water and large dissolved molecules through the mesh. Fabric sheets were folded on one side and heat sealed on two others. Each mesh bag was filled with approximately 0.5 g of dry ferrihydrite before the third side was heat sealed. Because ferrihydrite microaggregates may be smaller than the pore size of the mesh material, mesh bags were handled with care to minimise losses of mineral through the fabric during installation. The filled mesh bags were immediately transferred to the microcosms.

The microcosms were made from 500 mL plastic bottles with holes in the cap to maintain CO_2_ equilibrium, as illustrated in Fig. S1.† First, 100 g of sieved oven-dry soil was added to each microcosm. One mesh bag was laid horizontally on the soil in each microcosm and covered with a further 150 g of the same soil. Rhizon pore water samplers (Rhizosphere Research Products, Netherlands, 0.6 μm pore cut-off, custom filter length 20 mm) were placed into the dry soil vertically with porous filter at the depth of the mesh bag. Soils were flooded with 300 mL of 5 mM CaCl_2_ solution, which saturated and submerged all soils for the duration of the experiment. The CaCl_2_ was added to prevent dispersion of soil particles. After flooding, the microcosms were transferred to a glove box (MBRAUN, N_2_ atmosphere) and stored in the dark for the duration of the experiment.

Pore water was sampled (first 1–2 mL discarded, then ∼7 mL retained) from duplicate microcosms prior to sacrificial sampling and removal of mesh bags after one, two, six and twelve weeks of flooding. Pore water was drawn by vacuum through pre-positioned Rhizon samplers and additionally passed through 0.22 μm nylon filters (BGB, Switzerland) in the glove box. Additionally, pore water was extracted after one hour of flooding (in microcosms intended for twelve-week incubation) as an initial timepoint, before entry into the glove box. Reduction potential (Eh) and pH measurements were also taken in the microcosms prior to each sacrificial sampling, and additionally without sacrificial sampling after one day and four weeks. Reduction potential was measured using a series of redox probes (Pt with Ag/AgCl (3 M KCl) in-built reference) that were placed into each respective microcosm at the soil surface towards the corner of the microcosm on the day before reading. If readings did not stabilise overnight, a second probe was used to measure the Eh after 1–2 h stabilisation. Measured values were converted to Eh by adding 210 mV and corrected to remove small differences observed during the measurement of a standard (475 mV, Hamilton Company, Switzerland) with each probe. The pH was measured *in situ* in microcosm headwater, at the soil surface, with a single pH probe (Pt with Ag/AgCl (3 M KCl) in-built reference). The sampling strategy is illustrated in Fig. S1.†

During sacrificial sampling, mesh bags were removed from the soil with tweezers and allowed to dry under glove box atmosphere in the dark. In contrast to the powdered ferrihydrite at the beginning of the experiment, mesh bags containing (partially) transformed mineral comprised a single large aggregate. After ∼24 hours, the aggregates were carefully removed from the fabric. The aggregates were crushed and the powder homogenised with mortar and pestle. Mineral samples were stored in amber glass vials in the anoxic glove box atmosphere. For analysis of the spatial distribution of minerals inside the mesh bags, samples were taken from an additional set of replicate reactors of each soil after two weeks. These mineral aggregates were not homogenised once dried. Although regions with high ferrihydrite content were brittle when handled, sufficiently transformed samples maintained cohesive aggregate structure once removed from the mesh bags. These aggregates were cross sectioned with a stainless-steel blade and mounted on a glass slide with adhesive putty.

### Pore water analysis

Total element concentrations in the pore water were measured by ICP-OES and Fe speciation was measured by UV-VIS spectrometry (Cary 60, Agilent Technologies, USA) using colourimetric reaction with 1,10-phenanthroline (procedure adapted from Loeppert *et al.*^80^ whereby Fe(ii) was determined after an excess of nitriloacetic acid was added to mask the Fe(iii)^81^). Dissolved organic carbon (DOC) was measured with a DIMATOC 2000 carbon analyser (Dimatec, Essen, Germany). Anion concentrations were measured using ion chromatography (IC; 940 Professional IC Vario with Metrosep A sup 5–250/4.0 column, Metrohm, Switzerland) on non-acidified samples in glass vials, which were stored at −18 °C until measured.

### Mineral characterisation

X-ray diffraction measurements were performed on homogenised mineral contents of mesh bags from each microcosm. Approximately 20 mg of mineral powder was resuspended in ethanol and pipetted onto polished silicon wafers. Mineral abundance was estimated by Rietveld quantitative phase analysis (QPA) in TOPAS software (version 5, Bruker, USA). An empirically mass-calibrated *hkl* (PONKCS^82^) phase of pre-transformation ferrihydrite was used to quantitatively estimate the abundance of ferrihydrite in reacted samples.^18^ Details of the XRD analysis are available in Section 6 of the ESI.†

Electron microscope (EM) images were obtained on a scanning transmission electron microscope (STEM, 2700Cs, Hitachi) operated at 200 kV accelerating voltage. Under oxic conditions, approximately 2 mg of mineral was resuspended in ultra-pure water and drop deposited onto a 200-mesh Cu grid with a holey carbon support film (SPI supplies, USA). A secondary electron (SE) or a high angular annular dark field (HAADF) detector was used for image formation.

Raman spectroscopic analysis was carried out with an inVia 2 Raman Microscope (Renishaw, UK) using a 532 nm laser (100 mW, Nd-YAG), 1800 L mm^−1^ grating and 50× objective (Leica N-plan L). The expected laser spot size was 1.3 μm. Sample spectra were collected with 0.5% laser power for 8 s. The chosen parameters produced sufficiently strong signals for mineral identification at each measurement point with minimal beam damage, and maximised the number of locations that could be measured within ∼18 h (total measurement time chosen to minimise calibration drift). Automatic focusing (LiveTrack focus tracking^83^) was used to account for the rough surface. Example spectra (Fig. 4G, S14D and S15D†), were collected with the same instrument and optical set-up, and are the average of 50 spectra collected consecutively at the same location with 8 s exposure. Details of reference materials and measurement parameters are provided in Section 9 of the ESI.†

Images of the cross-section surfaces of unhomogenised mineral aggregates were collected using an optical microscope with digital camera (Stemi 2000-C with AxioCam ERc 5s, Zeiss, Germany), before Raman spectroscopic analysis. Additionally, samples from rim and inner core of the unhomogenised mineral aggregates were isolated using a scalpel, dissolved in 4 M HCl, and analysed for elemental content by ICP-OES.

### Processing of Raman spectra

All Raman spectra were processed by removing signals attributed to cosmic rays and subtracting the baseline (Wire 5.2 software, Renishaw, UK). A component analysis (with normalisation by subtraction of minimum value and scaling to unit variance; Wire 5.2 software, Renishaw, UK) was applied to each sample spectrum to create maps of mineral distribution and estimate mineral abundance in the sample. The component analysis used reference spectra of ferrihydrite, lepidocrocite, goethite and hematite (hematite produced by beam damage and used to check for beam damage, details in Section 9 of the ESI†). The size and distribution of regions with distinct mineral identity in the 1 μm-resolution μ-Raman spectral component analysis maps were measured using image processing techniques. Binary images were produced by identifying pixels with mineral content above 50% of the normalised total range measured in the sample. Image segmentation analysis on the binary images employed an unsupervised watershed model with 3 μm minimum distance between particle centres, implemented with the SciPy and Scikit-image analysis packages.^84,85^ Particle dimensions were measured with Scikit-image^85^ and nearest neighbour distances were calculated using a ball-tree model from the Scikit-learn package.^86^

## Results

### Pore water composition

**Fig. 1:**
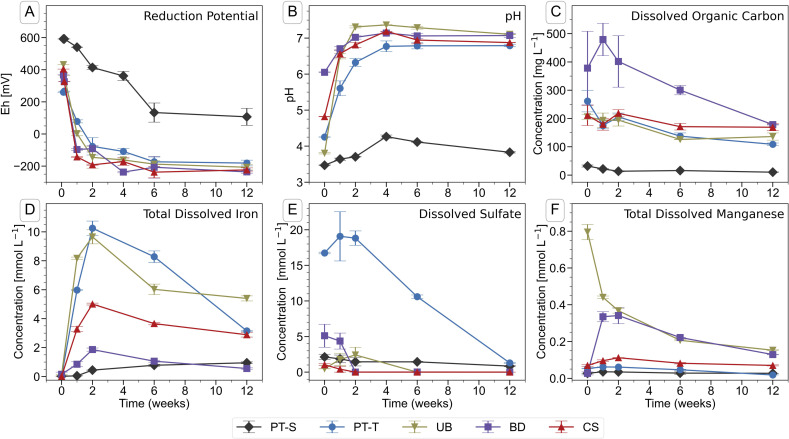
Chemical parameters in the pore water of soil microcosms during the course of the incubation experiment. Error bars indicate the range of measurements from duplicate microcosms. Data are presented in Table S3.†

Soil flooding led to a decrease in reduction potential (Eh) (Fig. 1A) and associated rise in pH (Fig. 1B) in the soil solutions during the first four to six weeks of incubation. The onset of reducing conditions occurred quickly in all four microcosms containing topsoil (PT-T, UB, BD, CS), and more slowly in the only microcosm containing subsoil (PT-S). In twelve weeks, the Eh fell to between −181 (PT-T) and −236 mV (BD) in the topsoils but did not fall below 100 mV in the subsoil. The pH of all flooded soils stabilised at 7.0 ± 0.2 after four weeks, apart from PT-S, which reached a maximum of 4.4 after four weeks.

Iron was released into solution within days of the flooding of the topsoils (Fig. 1D). Iron reduction in paddy soils typically begins below an Eh of ∼100 mV at pH 7,^2,87^ dependent on mineral and pore water characteristics,^6^ with abundant amorphous Fe(iii) phases reduced at ∼0 mV.^1,88^ In this study, the redox potential was below 0 mV in BD, UB and CS within one week, and shortly thereafter in PT-T (Fig. 1A). Within analytical uncertainty, all Fe measured in pore water at each timepoint was ferrous (Table S4†). The concentration of Fe in the PT-T soil solution reached 10.3 mM at its peak after two weeks of flooding, which corresponded to high soil Fe content (Table S2†) and strongly reducing conditions. Despite very low soil Fe content in UB, peak Fe concentration in pore water after two weeks was similar to that observed in PT-T pore water (9.6 mM). The other soils produced moderate (4.9 mM in CS) or low (1.8 mM in BD) peak Fe release into pore water. After peaks at two weeks, the Fe concentrations decreased in all soil pore waters for the remainder of the experiment, except in PT-S where dissolved concentrations rose continuously to a peak below 1.0 mM at the end of the experiment.

Dissolved sulfate (Fig. 1E and Table S3†) was initially highest in pore water from the acid-sulfate topsoil, PT-T (16.7 mM), but decreased throughout the experiment (to 1.29 mM at twelve weeks). Dissolved sulfate concentrations also fell in BD (5.1 mM initially, <0.001 mM after two weeks), UB (1.8 mM peak at one week, <0.001 mM after six weeks) and CS (1.0 mM initially, <0.001 mM after two weeks). Dissolved chloride concentrations (Table S4†) were dominated by the addition of 5 mM CaCl_2_ in the flooding water. Dissolved phosphate could not be detected by IC, but some P was measured by ICP-OES in CS and BD (up to 0.12 and 0.10 mM respectively; Table S4†). Dissolved silicon was highest in PT-S (up to 1.6 mM). Major cations measured in the pore water included Mg (up to 5.9 mM in BD and 4.9 mM in PT-T; Table S4†), Na (up to 13 mM in PT-T and up to 12.4 mM in PT-S; Table S4†), K (up to 1.1 mM in PT-S and 0.9 mM in PT-T; Table S4†) and Mn (up to 0.8 mM in UB; Fig. 1F and Table S3†).

The DOC concentration in pore water (Fig. 1C and Table S3†) was highest in the topsoils, with most released from soil BD (479 ± 57 mg L^−1^, week one), followed by soils PT-T (262 ± 38 mg L^−1^, initial), CS (219 ± 12 mg L^−1^, two weeks), UB (206 ± 13 mg L^−1^, initial), and PT-S (<32 ± 4 mg L^−1^ throughout). Release of DOC in the topsoils corresponded to the falling redox potential by enabling biological metabolism in the soils. The low DOC in PT-S, which could have limited biological activity and explained its low redox activity, corresponded to its low solid C content (0.7%, Table S1†) and the strong binding capacity of the clay minerals for OC (PT-S is 67% clay, Table S1†). In contrast, soil UB produced a high DOC release despite its low carbon content (0.8%).

### Iron mineral transformations: bulk measurements

**Fig. 2:**
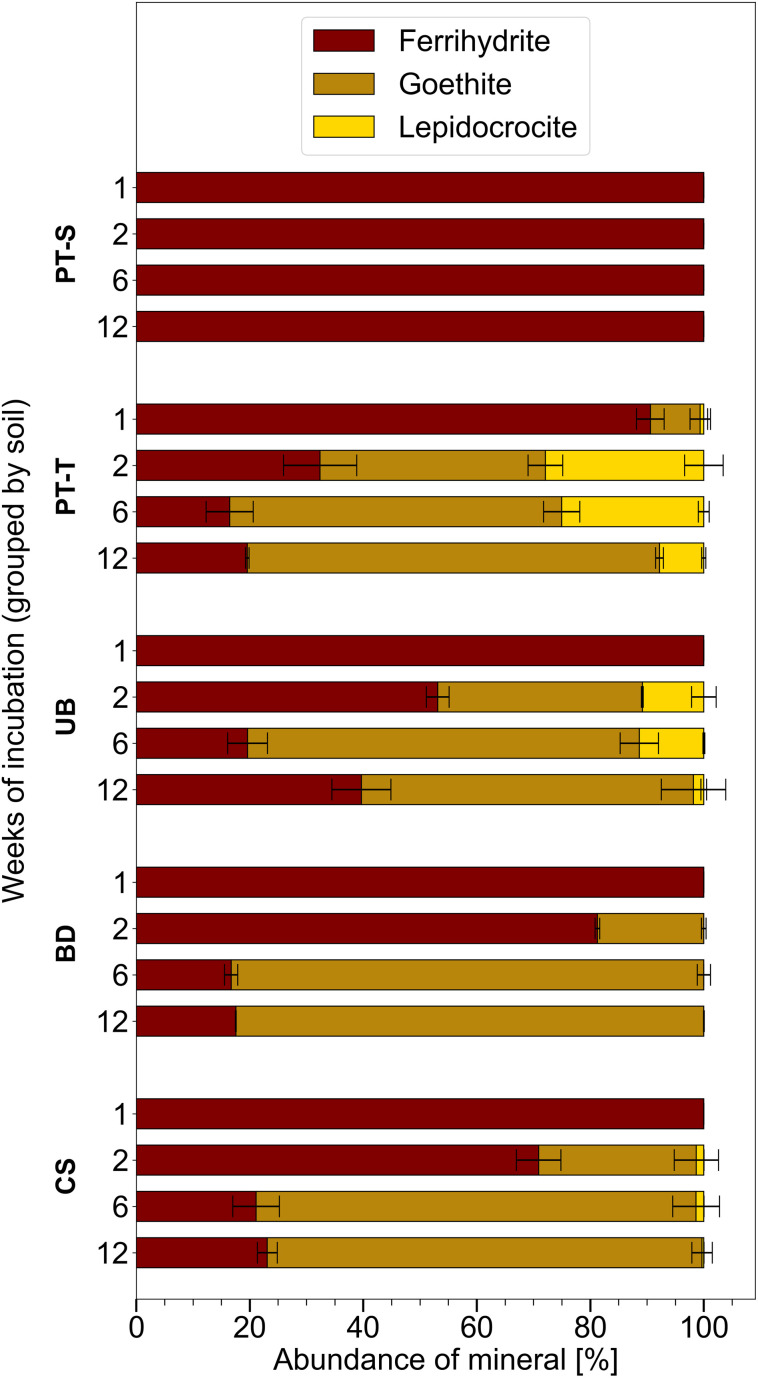
Proportion of Fe minerals measured in homogenised contents of mesh bags, based on Rietveld fitting of XRD patterns (Fig. S4–S9 and Table S6†). Proportions including non-ferric minerals are presented in Table S6.† Error bars indicate the range of measurements from duplicate microcosms (and only exceed 100% when compensated by smaller proportions of other minerals).

**Fig. 3:**
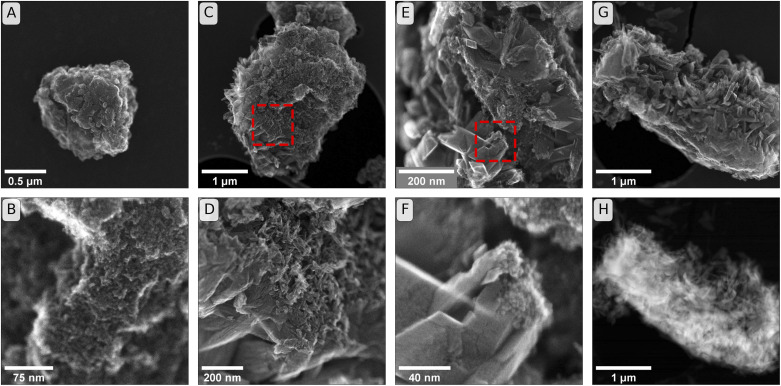
Secondary electron (SE) images of initial and reacted minerals. Panels (A & B): Initial ferrihydrite used in incubation experiments that were sampled at weeks one, two and six (comparison to starting material for twelve-week and Raman-cross-section experiments in Fig. S13†). Panels (C & D): SE images from incubations of ferrihydrite in soil BD after six weeks of incubation. (The frame of panel (D) is the region identified by the dashed square in panel (C)). Panels (E & F): SEM images from incubations in soil PT-T after six weeks of incubation. (The frame of panel (F) is the region identified by the dashed square in panel (E)). Panel (G): SE image from soil PT-T after six weeks of incubation. Panel (H): High angular annular dark field (HAADF) image of sample as shown in panel (G).

**Fig. 4:**
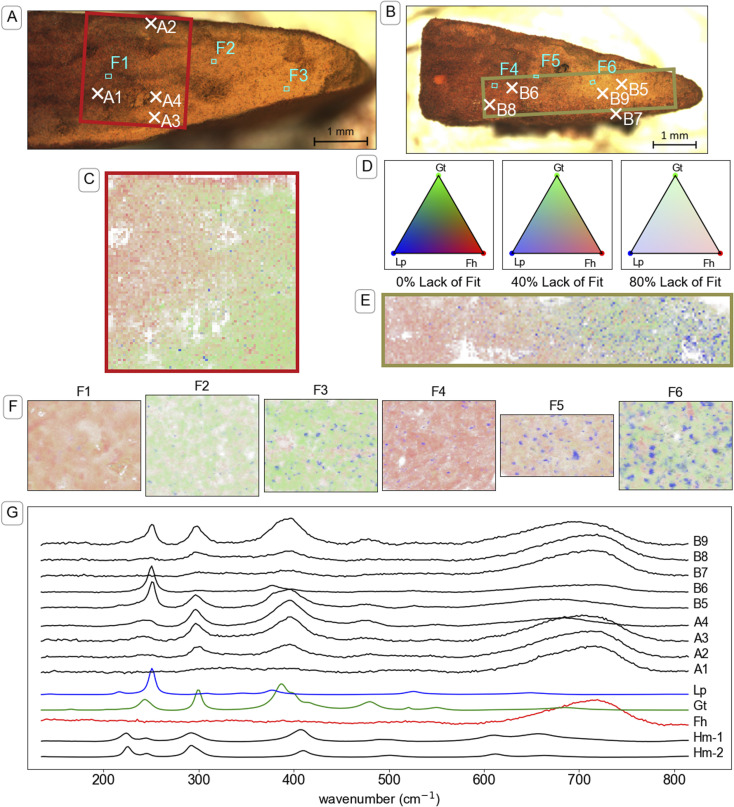
Investigation of the mineral identity in cross-sections of mineral aggregates from mesh bags incubated in two soils. Panel (A): Microscope image of a cross-section of a mineral aggregate that was incubated in CS soil for two weeks. The red box indicates the area that is mapped in panel (C) and the marked locations correspond to the maps in panel (F) (F1–3) and spectra shown in panel (G). Panel (B): Microscope image of a cross-section of a mineral aggregate that was incubated in UB soil for two weeks. The green box indicates the area that is mapped in panel (E) and the marked locations correspond to the maps in panel (F) (F4–6) and spectra shown in panel (G). Panels (C & E): Map of mineral identity on a cross-section of a mineral aggregate that was incubated in CS soil and UB soil respectively. Raman spectra at each point in panels (C, E and F) were analysed using component analysis with the reference spectra for ferrihydrite (Fh), goethite (Gt), lepidocrocite (Lp) and hematite (Hm) shown in panel (G). Panel (D): Colour legend for Raman spectral component analysis maps in panels (C, D and F). Colours are based on a Maxwell colour triangle^105^ where Fh, Gt and Lp are plotted as the red, green and blue colour components, respectively. The transparency of the colours is set according to the lack of fit in the component analysis, with bright colours corresponding with complete fits using the three available components, white corresponding to the calculated lack of fit component, and dark colours corresponding to regions where other spectra were included in the component analysis but not displayed on the chart, specifically a reference for beam damage (Hm-1), which matches a reference spectrum of hematite (Hm-2) (see explanation in Section 9 of the ESI†). Representations of panels (C, E and F) with grey-scale maps for individual components (eliminating red–green contrast) are available in Fig. S19–S23 and S28–S30.† Panel (F): Maps taken in the boxed regions F1–F6 in panels (A and B), and ordered left to right, respectively, with 1 μm resolution. Panel (G): Plot of Raman spectra taken at individual locations indicated in panels (A and B). The Raman spectra are truncated and normalised to standard range. Full spectra from 135 to 1900 cm^−1^ (Fig. S18†) were used for component analysis.

Ferrihydrite that was incubated in mesh bags in the topsoils was partially transformed to goethite and lepidocrocite within twelve weeks, whereas mineral transformation was not detected in mesh bags from the subsoil, PT-S (Fig. 2 and S4–S9 and Table S6†). Other Fe phases were not identified in the products. After one week, XRD patterns revealed that mineral transformations had only occurred in PT-T microcosms (9% transformation). By week two, the transformation in PT-T increased to 68% and transformation in the other soils began, with most observed in UB (47%) followed by CS (29%) and BD (19%). In all topsoils, most ferrihydrite transformation occurred within the first six weeks (77–84% transformation), and in three soils, between six and twelve weeks, the residual ferrihydrite fraction remained relatively stable. The estimation of ferrihydrite fractions with QPA based on XRD patterns is challenging when ferrihydrite contributes <10% of the total (Section 6 of the ESI†). However, the presence of ferrihydrite was confirmed in two-week reacted samples by Raman spectroscopy (Fig. 4G, S14 and S15†) and six-week reacted samples by the observation of <5 nm diameter spherical crystallites in EM images (*e.g.*Fig. 3F).

Bulk mineral transformation rates were estimated for ferrihydrite in PT-T, UB and CS by exponential decay fits of ferrihydrite abundance in the samples at weeks one, two, and six (Fig. S10†). Ferrihydrite in soil PT-T transformed fastest (rate coefficient is 3 × 10^−6^ s^−1^) followed by UB (1 × 10^−6^ s^−1^) and CS (1 × 10^−7^ s^−1^). Sampling density was not enough to model the exponential decay in BD satisfactorily. The ratio of lepidocrocite to goethite in the products varied between soils and over time. The highest lepidocrocite-to-goethite ratio was observed after two weeks with PT-T (0.72) > UB (0.30) > CS (0.04) > BD (no lepidocrocite observed by XRD). A smaller lepidocrocite-to-goethite ratio was observed in all transformed soils after six weeks with PT-T (0.43) > UB (0.16) > CS (0.01) > BD (no lepidocrocite observed). Between six and twelve weeks, when less ferrihydrite transformation was observed, some lepidocrocite was replaced by goethite, especially in PT-T (lepidocrocite-to-goethite ratio decreased to 0.12).

Based on the LVol-IB values obtained from Rietveld fitting of whole diffractograms, the crystallite size of lepidocrocite (73–113 nm in all samples, Fig. S11†) was much larger than goethite (20 nm in samples from PT-T, UB, and CS soils and 13–15 nm in samples from BD) in all samples (Fig. S12†). There was no clear size change of lepidocrocite crystallites in any sample until week six. Between weeks six and twelve, there was an increasing trend in the lepidocrocite crystallite size of samples from CS and UB soils (from 99 nm to 113 nm and from 73 nm to 100 nm, respectively) which cannot be definitively discerned from the uncertainty of the replicate measurements. Over the same period, the crystallite size of lepidocrocite in samples from PT-T fell simultaneously with the decrease in lepidocrocite-to-goethite ratio. There was no clear growth trend of average goethite crystallites in bulk samples during the experiment.

The secondary electron (SE) images, shown in Fig. 3, reveal changes in the mineral morphology within mesh bags during the incubation. Six-week reacted samples of BD (Fig. 3C and D) and PT-T (Fig. 3E–G) contained micro-aggregates of similar scale to ferrihydrite before incubation (Fig. 3A and B and S13†). Lepidocrocite and goethite crystals are closely associated with the apparent ferrihydrite micro-aggregates in SE images from both soils. The HAADF image in Fig. 3H shows the difference in thickness between the initial ferrihydrite micro-aggregate (bright interior) as opposed to the secondary growth minerals on the particle exterior. The growth of small needles on the ferrihydrite micro-aggregate exterior from soil BD can be attributed to goethite crystal growth, since XRD analysis showed that goethite was the only transformation product of ferrihydrite in this sample. The crystals that formed during incubation in PT-T were larger than the needles observed in soil BD. These crystals were likely lepidocrocite, corresponding to the larger LVol-IB of the lepidocrocite crystals measured by XRD, and the form of lepidocrocite crystals observed previously.^42^

Quartz and clay, which were detected in the mesh bags at trace levels (Table S6†), are abundant in these soils and were most likely introduced to the contents of the mesh bags through the permeable fabric during incubation or as contamination during sampling. In contrast, the rhodochrosite (MnCO_3_) that was fit in XRD patterns of mesh bag contents from soil BD most likely formed from solution in the mesh bags, because the amount increased at each consecutive timepoint and diffraction peaks consistent with rhodochrosite were not observed in powder XRD scans of BD soil (data not shown). Rhodochrosite was also not observed in XRD patterns of UB soil, or mesh bags contents from UB soil, despite higher pore water concentrations of Mn in UB than in BD (Fig. 1F). All mineral proportions in Fig. 2 and in the text refer only to proportions of each Fe mineral normalised to the total fraction of Fe minerals in the mesh bags, excluding quartz, kaolinite and rhodochrosite. Mineral proportions including non-Fe minerals are presented in Table S6.†

### Iron mineral transformations: spatially resolved measurements

Visual analysis of cross sections of the mineral aggregates from the mesh bags after two weeks of incubation revealed a millimetre-scale inhomogeneous spatial distribution of mineral products. Specifically, we noticed a rim, outer core and inner core (see illustration in TOC art). The millimetre-scale distribution of transformation products was crudely identifiable by visual analysis of the colours. For example, the identification of gradients from yellow–brown (corresponding to goethite^89^) to brown–orange (often corresponding to ferrihydrite^89^) was observed in optical microscope images, shown in Fig. 4A and B, S14A and S15A.† Mapping with μ-Raman spectroscopy was used to identify the relative abundance of mutually proximate (sub-micrometre-scale) minerals on a 25–35 μm grid across millimetre-scale colour gradients. Analysis of the Raman spectra confirmed that brown–orange regions were dominated by ferrihydrite and yellow–brown regions by goethite. Parts of mineral aggregates that were dominated by untransformed ferrihydrite were brittle when dry, indicating that the formation of a stable mineral aggregate was largely the result of mineral transformation reactions and not mechanical compression.

Brown–orange mineral was observed in the rim (<100 μm thick) of the mineral aggregates from all topsoils after two weeks of incubation. Raman spectra A2 and B7 in Fig. 4 are examples from the rim of mineral aggregates from CS and UB, respectively. Further example of spectra measured on the rim of mineral aggregates that were exposed to other soils are provided in Fig. S14 and S15.† Both Raman spectra A2 and B7 are dominated by a strong broad peak near 710 cm^−1^ consistent with the reference ferrihydrite spectrum, and contain small peaks near 299 and 386 cm^−1^ that indicate a minor goethite component. Although cross sections from other time points were not preserved for μ-Raman spectroscopy analysis, similar brown–orange-coloured coatings were observed on samples from CS (Fig. S3†) and UB at the twelve-week timepoint. Elemental analysis of samples from the exterior (mostly rim) and interior (inner core and outer core) after two weeks of reaction, presented in Table S5,† contained elevated concentrations of various elements in the exterior compared to the interior, including Al, Ca, P, and Si.

Underneath the rim, an outer core of ferrihydrite transformation products formed towards the edges of the mineral aggregates, most prominently near the narrow corners of the mesh bag (which form the pointed shape of mineral fragment, for example on the right of fragments in Fig. 4A and B). Raman spectra from the outer core of the mineral aggregates, such as spectra A3, A4, B5, B6 and B9 in Fig. 4, revealed higher abundance of both goethite and lepidocrocite compared to spectra measured on the rim. Further examples of spectra measured in the outer core of mineral aggregates that were exposed to other soils are provided in Fig. S14 and S15.† Lepidocrocite was not easily distinguishable from goethite or ferrihydrite by eye, despite its higher chroma,^89^ but μ-Raman spectroscopy analysis indicated large proportions of lepidocrocite in the yellow regions of some samples. The lepidocrocite-to-goethite ratios estimated by component analysis averaged across the maps were largely consistent with the XRD analysis of bulk samples (Fig. 2) within the bounds of quantification by the two methods (see comparison in Table S7†). In microcosms where lepidocrocite formed, it was largely co-located with goethite on a millimetre scale. The co-location was observed in microcosms with both high (*e.g.* UB, Fig. 4B) and low (*e.g.* CS, Fig. 4A) lepidocrocite-to-goethite ratio.

Large regions of brown–orange mineral, likely ferrihydrite, occurred in the inner core of the mineral aggregates from soils CS, UB and BD. The transition from the ferrihydrite-dominated inner core to the transformed outer core was gradual. Examples of spectra from the residual ferrihydrite in the inner core of mineral aggregates from CS and UB (Fig. 4, spectrum A1 and B8, respectively) strongly correspond to the standard spectrum of ferrihydrite. Example spectra from the inner core of mineral aggregates from soil BD are presented in Fig. S14.† In PT-T, the inner core was no longer discernible from the outer core by the two-week timepoint (Fig. S14†). Nonetheless, there was an uneven distribution of transformation products, with lepidocrocite most abundant nearer the top edge of the aggregate (Fig. S36†), and around intrusions of an unidentified substance, visible as large areas of Raman-inactive compounds, where the ‘lack-of-fit’ component is high (Fig. S35†).

Within the parts of the mineral aggregates containing abundant transformation products (outer core, and transition to the inner core), μ-Raman spectral component analysis mapping revealed a non-random distribution of minerals at the micrometre scale (Fig. 4F, S35–37 and S45†). Residual ferrihydrite was identifiable, dispersed among regions dominated by goethite or lepidocrocite. Mixed goethite–ferrihydrite (*e.g.* spectrum A3, Fig. 4) Raman spectra were common, as can be seen in strongly overlapping fractional abundance distributions (Fig. S25–S27, S32–S34 and S42–S44†), whereas goethite–lepidocrocite mixed spectra (spectrum B5 and B9, Fig. 4) were less common, as lepidocrocite tended to grow in mineralogically distinct regions. Fractional abundance distributions show that most measurements did not detect lepidocrocite because lepidocrocite preferentially occurred as a near-pure phase.

The spatial separation of lepidocrocite from other minerals made it possible to analyse lepidocrocite particle size distribution by segmentation analysis. The lepidocrocite regions that were identified by segmentation analysis (visualised in Fig. S24, S31 and S41†) were analysed for area, abundance and mutual proximity. The measured segment areas (1.7–13.1 μm^2^) were larger than the spot size of the Raman laser beam (1.3 μm^2^). In UB, the number and size of lepidocrocite segments were negatively correlated with ferrihydrite abundance in measurement areas (<100 × 100 μm) across the gradient between inner and outer core (Table S7†). In CS, lepidocrocite segments were similarly most abundant in areas (<100 × 100 μm) where ferrihydrite was least abundant, but segment area trends were less reliable because lepidocrocite segments were small in comparison to the 1 μm measurement resolution and 1.3 μm spot size (Fig. 4 and Table S7†). In BD, little transformation of ferrihydrite to lepidocrocite occurred and therefore segment statistics were unreliable (data not shown). In PT-T, patterns of lepidocrocite growth could be measured by segmentation analysis, but the inner core had reacted within two weeks and could not be differentiated from the outer core. Particle segmentation analysis was most successful in samples that had undergone only a moderate amount of transformation after two weeks.

## Discussion

### Influence of pore water chemistry on bulk transformation rates and pathways of ferrihydrite transformation

The Fe mineral transformation rates and product composition in the mesh bags reflected the geochemical conditions of the pore water during soil flooding, particularly the Fe(ii) concentration, as summarised by the conceptual diagram in Fig. S2.† The peak Fe(ii) pore water concentration, the degree of bulk transformation at week two and lepidocrocite-to-goethite ratio at weeks two, six, and twelve each followed the order PT-T > UB > CS > BD. The general trend observed in this experiment agrees with previous mixed-suspension experiments in which Fe(ii) concentration has been called the “master variable”.^21^ Nonetheless, bulk transformation rate coefficients in this study, calculated by an exponential decay model to be between 1 × 10^−7^ s^−1^ and 3 × 10^−6^ s^−1^, are approximately one to three orders of magnitude slower than the first-order bulk transformation rate constants in mixed-suspension experiments for ferrihydrite transformation to goethite and lepidocrocite in the presence of Fe(ii) (0.1–0.7 w/w Fe(ii)-to-ferrihydrite ratio) at circumneutral pH (5–8) and room temperature.^34,36,37^ For example, the transformation of 20 g L^−1^ ferrihydrite with 50, 100 or 250 mM Fe(ii) (0.14, 0.28 and 0.7 Fe(ii)-to-ferrihydrite, w/w, respectively) proceeded with first-order rate constants between 2 × 10^−5^ and 5 × 10^−5^ s^−1^ in two independent experiments^34,36^ and an experiment with 500 mg L^−1^ ferrihydrite and 1 or 3 mM Fe(ii) (0.11 and 0.33 Fe(ii)-to-ferrihydrite, w/w) produced first-order rate coefficients of labile Fe(iii) formation and transformation of labile Fe(iii) to lepidocrocite and goethite in the range of 1 × 10^−5^ s^−1^ to 25 × 10^−5^ s^−1^.^37^ In contrast, we observed transformation rates one to three orders of magnitude faster than rates (between 9.7 × 10^−9^ s^−1^ and 5.6 × 10^−8^ s^−1^) reported in a study that used sequential extractions to measure the rate of synthetic ferrihydrite transformation to crystalline products following mixing with paddy soils at 70% water holding capacity that contained pore water with peak Fe(ii) concentrations between ∼0.01 and ∼0.09 mM.^68^

Slower rate constants of ferrihydrite transformation in the mesh bags imply that the transformation rate was influenced by the spatial arrangement of components in the non-mixed system. The rate of Fe(ii)-catalysed ferrihydrite transformation depends on the association of Fe(ii) to ferrihydrite surfaces, where the local Fe(ii) concentration has a direct influence on the amount of surface-associated Fe(ii).^37^ In our microcosms, the ferrihydrite was concentrated inside the mesh bags, while Fe(ii) was present throughout the microcosms. Two important assumptions can be made about the mesh bags: firstly, that the composition of the pore water in the mesh bags approached that of the soil solution over time due to diffusion from high to low concentration regions, and secondly, that the ferrihydrite mesh bags in all microcosms were initially identical, and therefore the Fe(ii) concentration in the pore water acts as a proxy for the surface-associated Fe(ii) for the purposes of comparison between studies. Although the total amount of Fe(ii) in the system as a ratio of ferrihydrite (up to 0.3 w/w in PT-T at week two) was similar to previous mixed-suspension experiments (between 0.1 and 0.7, w/w),^34,36,37^ Fe(ii) in our system most likely originated in the soil, and diffusion into the mesh bags was required for Fe(ii)-catalysed ferrihydrite transformation. Therefore diffusion limitation of Fe would have restricted the surface association of Fe(ii) with the ferrihydrite, thereby lowering the transformation rate. Furthermore, Fe(ii) concentrations in soil pore water did not reach their peak until two weeks into the experiment. Although the relatively high ferrihydrite-to-solution ratio in the mesh bags in comparison to mixed-suspension experiments could have accelerated the transformation process,^44^ the Fe(ii) concentration in pore water of our microcosms was lower than concentrations used in mixed-suspension experiments.^34,36,37^ The slow transformation rate would have also been influenced by ferrihydrite in the rim, that contributed to the pool of remaining ferrihydrite in measurements of bulk mineral composition.

The relative favourability of goethite or lepidocrocite formation from Fe(ii)-catalysed ferrihydrite transformation is heavily influenced by the interaction of dissolved Fe(ii), mineral-sorbed Fe(ii), mineral-bound Fe(iii), and other ions in the system, by mechanisms that are still debated.^21,23,37,42,43,45,49,51,60^ Lepidocrocite is the favoured product when surface-associated concentrations of Fe(ii) are lower, because more surface-associated Fe(ii) ions enable more electron transfer reactions that convert lepidocrocite to goethite.^21,37,42^ However, sorption of ligands on product mineral surfaces may prevent clear correlation between the amount of Fe(ii) surface adsorption and abundance of transformation products in an experimental system.^42^ Lepidocrocite is favoured over goethite in media containing high chloride concentrations.^21,42,51^ Hansel *et al.*^21^ demonstrated that a 10-fold increase in Fe(ii) concentration decreased the lepidocrocite-to-goethite ratio in transformation products, but that lepidocrocite remained most abundant over 130 hours when the Fe(ii) was added as FeCl_2_ rather than FeSO_4_. Their fits of the initial mineral transformation rates show that lepidocrocite is formed faster than goethite regardless of Fe(ii) concentration in the presence of chloride (and with the presence of sulfate their reported rate of lepidocrocite formation is underestimated because of the low temporal sampling density).^21^ The stabilisation of lepidocrocite by chloride may be complementary to stabilisation of lepidocrocite by other dissolved ions that hinder transformation of lepidocrocite to goethite during Fe(ii)-catalysed transformation of ferrihydrite^20,48^ and greater nucleation of lepidocrocite in high Cl media.^42^

In our experiments, we observed a relationship linking high peak Fe(ii) concentrations in pore water with higher lepidocrocite-to-goethite ratios in the products, as outlined in Fig. S2.† Less lepidocrocite was observed at week twelve than week six in samples from all soils that induced lepidocrocite formation, consistent with the Fe(ii)-catalysed transformation of lepidocrocite to more thermodynamically stable minerals such as goethite.^37,43,50^ Yet the dissociation of goethite and lepidocrocite in 1 μm-scale Raman spectral component analysis maps, and close association of ferrihydrite with both lepidocrocite and goethite in SE images, indicate that both lepidocrocite and goethite form at the ferrihydrite mineral surface. Due to the addition of 5 mM CaCl_2_ in our study, chloride was present at similar concentrations in pore water from each soil, possibly inhibiting the transformation of lepidocrocite to goethite.^21^ Most likely, the simultaneous formation of lepidocrocite and goethite is explained by the nucleation of a labile Fe(iii) species on the surface of existing ferrihydrite,^37,42,43,45^ with the nature of the nucleation being dependent on Fe(ii) diffusion through the mesh bag, and decisive for the identity of the product mineral in a competitive crystallisation process.^21,42,43^ The prevalence of the less thermodynamically stable products, such as lepidocrocite, in samples containing high Fe(ii) concentrations, might be explained by stabilisation of those mineral phases with ions, such as chloride.^21^

Various ions in solution can affect the rates and pathways of ferrihydrite transformation, but their effect may be masked by the dominant influence of Fe(ii). In some cases, the pH can alter ferrihydrite transformation rates and pathways. At pH below the point of zero charge of ferrihydrite, Fe(ii)-catalysed transformation is hindered due to competitive sorption with H^+^.^37,54^ However, in the acidic pH range, the dissolution of ferrihydrite by H^+^ occurs more slowly than Fe(ii)-catalysed transformation and, therefore, may not be observed in the timeframe of this experiment.^68,90^ In the present study, ferrihydrite was fastest to transform in soils with low pH in the first week of the study, but this trend was also explained by Fe(ii) concentration and the relative pH of each soil after week two does not correspond to the rate of ferrihydrite transformation. Lower pH in an environmentally relevant range may also initially favour a higher lepidocrocite-to-goethite ratio.^37^ In this study, acidic conditions (Fig. 1B) may have supported the high lepidocrocite formation in PT-T and UB early in the experiment, but in the elevated pH of UB by week two, would have been antagonistic to ongoing lepidocrocite formation. The effect of some dissolved ions on the bulk transformation rates and pathways may have been limited to the rim (at the soil-mineral interface) because of diffusion limitations (Table S5†).

Iron sulfide minerals, mixed-valence Fe oxides and ferrous minerals were not observed in XRD patterns of mesh bag contents. Sulfate was the dominant S species observed in solution in the early sampling points (Fig. 1E), and was measured in high concentrations from the acid sulfate soil, PT-T. Decreasing concentrations of sulfate were likely associated with reduction of sulfate to sulfide. Sulfide is the dominant inorganic reductant of Fe-oxyhydroxide-associated Fe(iii) in sulfidic sediments.^5^ However, it is quickly removed from pore water solution in the presence of Fe(ii) or other chalcophile metals that form metal sulfide minerals,^5,91,92^ may be oxidised by Fe(iii) to other S species such as sulfite, elemental sulfur, polysulfides or thiosulfate,^5^ or may react with organic matter.^93^ It is likely that any number of these processes removed sulfide from pore water in the microcosm soil before it could diffuse into the mesh bags. The products of ferrihydrite transformation also did not include detectable ferrous minerals, such as siderite, or mixed-valence Fe minerals, such as magnetite or green rust, despite the strong Fe-reducing conditions. Ferrihydrite can transform into magnetite in the presence of sufficiently high Fe(ii) surface loading of ferrihydrite.^38,39,59,94^ In this experimental set-up, diffusion limitations on the supply of Fe, the large ferrihydrite surface area inside the mesh bag, presence of other ions sorbed to ferrihydrite, and the low pH during early stages of the experiment, may have prevented sufficient Fe(ii) surface sorption to produce magnetite.

### Spatial distribution of ferrihydrite and its transformation products

Gradients of mineral abundance were observed on the millimetre scale in the μ-Raman spectral component analysis maps of mineral aggregate cross sections made after two weeks of incubation. Distinct layers, referred to here as the rim, outer core and inner core, were identified in aggregates from soil CS, UB, and BD. In contrast, aggregates from soil PT-S contained no observable transformation products at week two and aggregates from soil PT-T, although mineralogically transformed, did not show clear layering. The complete transformation of ferrihydrite in soil PT-T confirms that the penetration of pore water into mesh bags buried in heavy clay soil was not limiting ferrihydrite transformation in the inner core of the mineral aggregates, and we expect this applies to the other coarser grained soil as well. Nonetheless, as Fe was reductively dissolved in flooded soil, it is likely that an Fe(ii) diffusion/reaction front advanced from the soil into the mesh bag, catalysing mineral transformation in the outer core before the inner core. Indeed, the products of ferrihydrite transformation, goethite and lepidocrocite, were least abundant in the inner core of aggregates from soil CS, UB, and BD compared to the outer core, corresponding to the effects of an Fe(ii) diffusion front. Despite mineral transformation in the outer core, within two weeks, the Fe(ii) had catalysed less transformation of ferrihydrite in the rim of mineral aggregates (at the soil–mineral interface) than outer core of aggregates from soils PT-T, UB, CS, and BD. We speculate that while Fe(ii) likely interacted with the mineral surface at the soil–mineral interface, the presence of some non-Fe ions or compounds in the soil solution may have led to ferrihydrite recrystallisation rather than transformation in the rim.

Numerous soil components could have inhibited the transformation of ferrihydrite in the rim, but it was not possible to identify which were responsible for the inhibition observed in this study. Phosphate,^22^ silica,^17,50^ Al,^23^ DOC,^17,55^ and various divalent metal cations^19,95^ have been shown to inhibit ferrihydrite transformation in model systems. Large organic compounds have also been shown to inhibit atom exchange by limiting sorption of Fe(ii) to ferrihydrite and to inhibit polymerisation of crystalline Fe (oxyhydr)oxides.^17^ Similarly, silica can react with corner-sharing sites of Fe octahedra, suppressing the polymerisation of 3-dimensional Fe oxyhydroxide crystals and instead directing the formation of ferrihydrite.^96^ Many of these pore water components have strong binding affinity to ferrihydrite surface sites, increasing their concentration and transformation-inhibition effect at the soil–mineral interface. In our microcosms, higher concentrations in pore water of chemical species that are known to inhibit ferrihydrite transformation were not necessarily associated with slower transformation of ferrihydrite in the rim. For example, the rim of mineral aggregates from soil BD were reacted within twelve weeks despite high concentrations of DOC (peak after one week of 6.7 mg L^−1^), P (peak after two weeks of 0.10 mM), Si (peak after two weeks of 1.1 mM) and Mn (peak after two weeks of 0.34 mM). By contrast, a prominent ferrihydrite rim was observed inside a mesh bag from soil CS after twelve weeks, despite low concentrations of known transformation inhibitors apart from P (peak after two weeks of 0.12 mM).

Whereas the diffusion of non-Fe soil components into the mineral aggregates may have been limited by sorption to the ferrihydrite surface near the soil–mineral interface, the diffusion of Fe(ii) species may have been less inhibited because aqueous Fe(ii) that was immobilised by adsorption to ferrihydrite was replaced by newly reduced Fe atoms through a process of electron transfer and atom exchange. This unique dynamic of the Fe(ii) interaction with Fe(iii) mineral may explain how Fe could diffuse deeper into the outer core than other ions during the first two weeks of the experiment. As the Fe(ii) diffusion/reaction front proceeded, lepidocrocite and goethite were gradually formed within the outer core. The μ-Raman spectral component analysis maps with 1 μm resolution revealed that ferrihydrite, goethite and lepidocrocite tended to occur in a non-random distribution, forming a patchwork of regions of dominant mineral identity that were several micrometres in diameter. The ferrihydrite hotspots in the μ-Raman maps correspond to the scale of the micro-aggregations observed in the SE images of untransformed ferrihydrite (Fig. 3). The SE images of reacted minerals show that the ferrihydrite micro-aggregations may give rise to newly formed goethite or lepidocrocite on their outer surfaces. The size of the lepidocrocite hotspots measured by segmentation analysis in the μ-Raman spectral component analysis maps (1.7–13.1 μm^2^ area, Fig. S24, S31 and S41†) were larger than the Rietveld-estimated crystallite size (0.073–0.113 μm diameter), indicating that crystals of mineral products tend to be grouped in regions of dominant mineral identity. This may reflect a tendency for newly crystallised mineral products to build on the template of pre-existing minerals.^97^

The comparison of mineral abundance across the reaction front (Table S7†) as measured by 1 μm-resolution μ-Raman spectral component analysis maps, indicates that lepidocrocite and goethite both formed as the Fe(ii) diffusion front progressed. This agrees with recent observations of simultaneous goethite and lepidocrocite nucleation on the surface of ferrihydrite during Fe(ii)-catalysed transformation.^42^ The fractional abundance distribution histograms (Fig. S25–S27, S32–S34 and S42–S44†) show that ferrihydrite and goethite both have peaks of occurrence in pixels of mixed mineral identity, with the peak of goethite measurements gradually shifting to higher fractional abundance with time since the progression of the Fe(ii) diffusion/reaction front. On the other hand, lepidocrocite showed an increasingly even fractional distribution histogram with progression of the reaction front. Notwithstanding the evidence of lepidocrocite replacement by goethite in the bulk mineral measurement, the unique growth behaviours of lepidocrocite and goethite is further evidence that lepidocrocite and goethite tend to form independently and could be driven by different reaction mechanisms.

### Application of μ-Raman spectral component analysis mapping to identify mineral abundance gradients

We have shown that Raman spectroscopy can provide new insights into the spatial distributions of Fe minerals during mineral transformation processes. Although changes in Fe mineralogy can be visually identified based on colour,^89^ or using bulk methods on coarsely sampled regions across geochemical gradients,^98–100^ μ-Raman spectroscopy provides effective high resolution (∼1.3 μm) identification and semiquantification of Fe minerals across gradients on the micrometre-to millimetre-scale. Examination of individual spectra (*e.g.*Fig. 4) provides verification that the component analysis effectively identifies the characteristic peaks of the reference minerals, and therefore qualitative analysis of the mineral distribution maps is robust. Although Raman spectroscopy is fundamentally quantitative,^101,102^ some measurement conditions that affect peak intensities could not be controlled in this experiment. Raman spectral component analysis maps produce internally consistent trends, but the absolute concentration values should be considered semiquantitative. Further discussion of the limits to quantification, elements of the Raman spectra that could not be fit in the component analysis (coloured white in spectral maps, *e.g.*Fig. 4C and E) and the effects of beam damage are provided in Section 8 of the ESI.† A direct comparison of the quantification of minerals by XRD and Raman spectral component analysis is presented in Table S7.†

### Environmental relevance

The transformation rates and pathways of ferrihydrite in soil depend on the chemical composition of soil pore water. The strong relationship between higher Fe(ii) concentration in pore water, faster ferrihydrite transformation, and higher lepidocrocite to goethite ratios in products, confirms the dominant effect of the Fe(ii) concentration on the rate and pathway of ferrihydrite transformation in flooded paddy soils.^21^ Nonetheless, other components of the pore water can explain various effects observed in the experiment. Ferrihydrite at the soil–mineral interface (mesh bag rim) was preserved longer than ferrihydrite in the outer core, which may be due to stabilisation of ferrihydrite in the rim against transformation by interaction with other components of the soil. Further, the correspondence between higher concentrations of Fe(ii) in pore water and greater lepidocrocite formation with respect to goethite in transformation products differs from results observed in mixed-suspension experiments^21,37,42^ and may indicate the importance of foreign ions for the stabilisation of lepidocrocite relative to goethite in these complex media.

In addition to the chemical complexity, the arrangement of the components in the non-mixed soils influenced the transformation of ferrihydrite and offered new insights into the spatial arrangement of the products of ferrihydrite transformation. Ferrihydrite transformation to lepidocrocite and goethite was fastest in the outer core, where the diffusion limitation on the availability of Fe(ii) was least in the beginning of the experiment. Although the formation of magnetite^38,39,59,94^ and iron sulfide minerals^5,91–93^ might have also been expected to occur in the presence of high Fe(ii) and sulfide concentrations, respectively, limited diffusion rates into the mesh bag and loss of sulfide from the solution by reaction with other soil components could have hindered the formation of these products inside the mesh bags. The association of both lepidocrocite and goethite with the surface of ferrihydrite and disassociation of goethite and lepidocrocite on the micrometre scale, as observed in SE images and μ-Raman maps, confirm the importance of independent nucleation of these mineral phases and suggest that micro-environments in non-mixed media support the growth of one or the other. These effects demonstrate the potential influence of soil heterogeneity on mineral transformation processes, which can only be realistically simulated in mineral transformation experiments using non-mixed porous media.

While the results of this study are largely consistent with ferrihydrite transformation in other laboratory experiments, mineral transformation in soils depends on a range of geochemical effects in complex non-mixed porous media. In contrast to ferrihydrite transformation observed in mixed-suspension systems,^34,36,37^ bulk transformation of ferrihydrite in this study, using mesh bags containing 0.5 g of pure mineral in flooded paddy soils, was one to three orders of magnitude slower. As ferrihydrite is abundant in soils,^25,26^ thermodynamically unstable with respect to other Fe mineral phases,^30^ and has a large surface area and density of surface sites that can interact with other chemical species in soil,^27–29^ its transformation can strongly influence the biogeochemistry of flooded paddy soils. The rate of transformation of ferrihydrite observed in this study potentially implies that the transformation of ferrihydrite influences the geochemical conditions of soil for months after initial flooding and could affect the agronomic conditions of crops such as rice, which grow in soils that are saturated or submerged for weeks at a time, throughout the growing season.^103,104^ The unique results of this study in comparison to previous works highlight the importance of studying mineral transformations in environmentally-relevant conditions in order to understand the biogeochemical processes that occur in complex soil environments.

## Author contributions

A. G. – conceptualisation, methodology, investigation, formal analysis, visualisation, writing – original draft; L. T. A. – conceptualisation, methodology, writing – review & editing; K. S. – methodology (mesh bags), writing – review & editing; K. R. – methodology (Raman spectroscopy), writing – review & editing; R. Kaegi – investigation (electron microscopy), writing – review & editing; R. Kretzschmar – conceptualisation, funding acquisition, methodology, writing – review & editing.

## Conflicts of interest

There are not conflicts to declare.

## Supplementary Material

EM-024-D2EM00290F-s001
