# Supplementary material for: Ferrihydrite transformations in flooded paddy soils: rates, pathways, and product spatial distributions
Source: Environ Sci Process Impacts. 2022 Sep 22;24(10):1867–82. doi: 10.1039/d2em00290f (PMC9580987; doi:10.1039/d2em00290f)
Supplement: EM-024-D2EM00290F-s001 [file EM-024-D2EM00290F-s001.pdf]

Electronic Supplementary Material (ESI) for Environmental Science: Processes & Impacts.
This journal is © The Royal Society of Chemistry 2022

Electronic Supplementary Information for:

Ferrihydrite in flooded paddy soils: reaction rates, transformation pathways and product spatial distributions

Andrew R. C. Grigg[a], Laurel K. ThomasArrigo[a], Katrin Schulz[a], Katherine A. Rothwell[a], Ralf Kaegi[b], Ruben Kretzschmar[a]

[a] *Soil Chemistry Group, Institute of Biogeochemistry and Pollutant Dynamics, Department of Environmental Systems Science, ETH Zurich, Universitätstrasse 16, CHN, CH-8092 Zurich, Switzerland*

[b] *Eawag, Swiss Federal Institute of Aquatic Science and Technology, Überlandstrasse 133, CH-8600 Dübendorf, Switzerland*

(54 pages, 46 figures, 7 tables)

Table of Contents

1. Soil Characteristics 2
2. Experimental Procedure 3
3. Additional pore water analysis 5
4. Elemental content of mineral layers in mesh bags 7
5. Photographs of mineral sampling at week twelve 8
6. XRD patterns 9
7. Crystallinity of products 19
8. Additional secondary electron (SE) images 20
9. Additional information about μ-Raman spectroscopy mapping 21
10. References 54

In addition to the information contained in this electronic supplementary information document, related code and data are available from the ETH Zurich Research Collection digital repository (https://doi.org/10.3929/ethz-b-000569468),[1] including:

- python code and source data required to reproduce all figures;
- python code required to do statistical analysis; and
- all Raman spectral data that was used in the component analysis.

1. Soil Characteristics

Table S1: Properties of the soils used in the microcosm experiments. [a] Soil texture measured by the Laboratoire et Bureau d'Etude au Service de l'Agriculture et de la Protection de l'Environnement (Gland, Switzerland). [b] The pH is measured with a 1:5 ratio of dry soil to 0.01 M $CaCl_2$.

Location					Texture [a]			pH [b]	Total C
Soil Code	Region	WGS84 coordinates		Depth	Clay (%)	Silt (%)	Sand (%)		(%)
PT-T	Pathum Thani province, Thailand	N 14.16°	E 100.81°	0-15 cm	61.0	36.2	2.7	4.10	2.83
PT-S	Pathum Thani province, Thailand	N 14.16°	E 100.81°	~1 m	67.4	24.0	8.6	3.24	0.74
UB	Ubon Ratchathani province, Thailand	N 15.42°	E 104.58°	0-15 cm	5.3	24.6	70.1	3.73	0.79
BD	Munshiganj district, Bangladesh	N 23.53°	E 90.30°	0-10cm	36.0	49.0	15.1	5.81	1.90
CS	Hunan province, China	N 28.44°	E 113.58°	0-50 cm	18.1	27.0	54.9	4.56	1.40

Table S2: Element concentrations in soils used for the microcosm experiments, measured by XRF. *measured on a sample of soil from same batch as that used in experiment. † Average values of measurements of samples taken from four locations in the same rice paddy field from which a large sample was taken for use in the laboratory experiment.[2]

Soil Code	Fe	Al	Si	Ca	Mg	S	P	V	Mn	Co	Ni	Cu	Zn	As	Pb
	($g\ kg^{-1}$)					($mg\ kg^{-1}$)									
PT-T*	38.0	87.1	218	3.2	2.1	2625	430	133	97.4	31	24	23	42	11	26
PT-S*	21.0	101	226	0.7	3.5	620.9	210	121	80.0	23	24	20	44	6.7	18
UB*	11.0	19.1	333	0.4	<0.02	427.6	310	28.7	120	38	2.1	5.7	9.7	2.0	6.5
BD*	44.3	87.9	217	8.3	12.6	651.2	590	125	438	38	53	49	96	13	40
CS †	15.1	113	246	1.5	2.7	637.0	710	23.3	172	17	5.7	13	86	5.2	69

2. Experimental Procedure

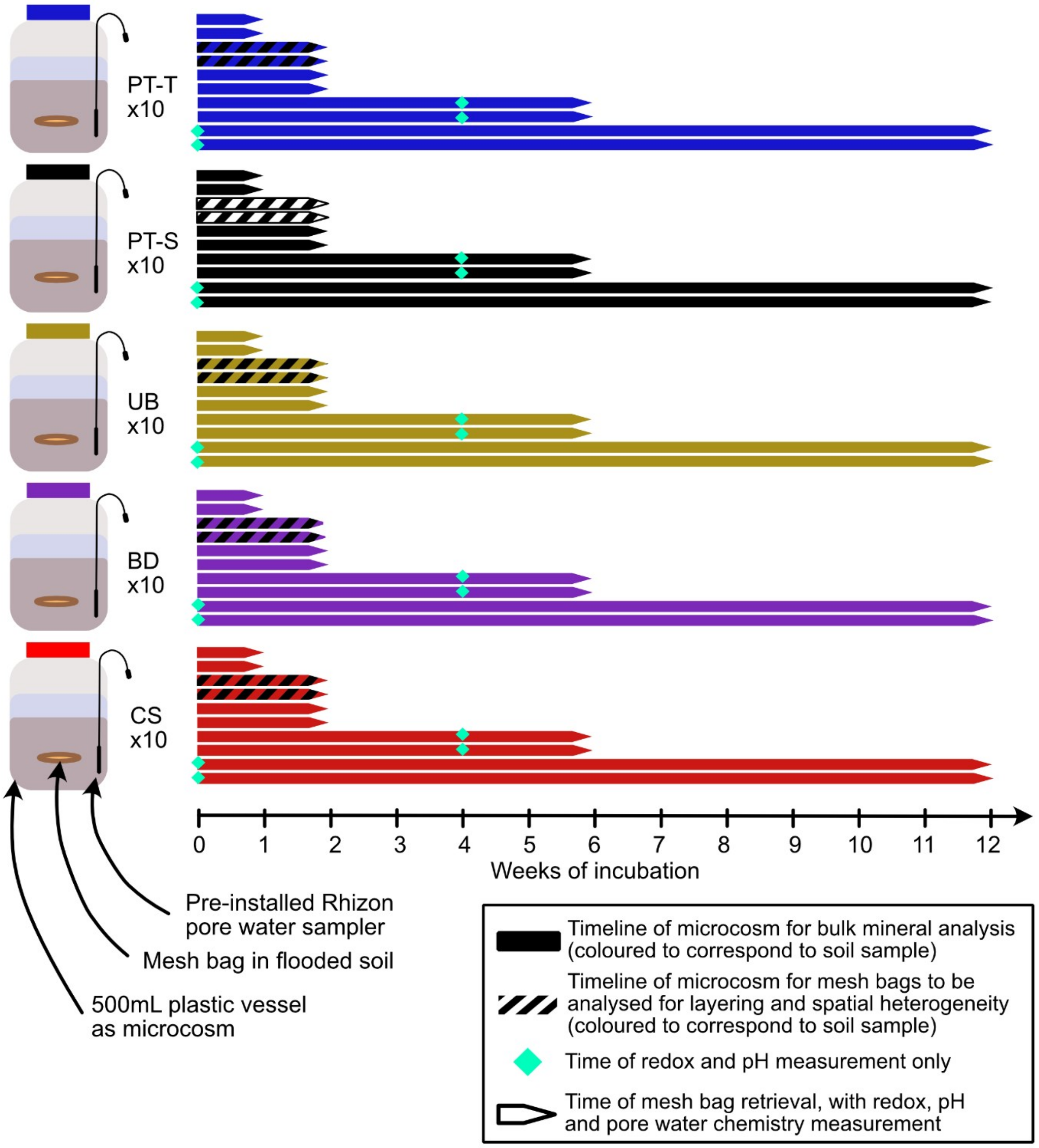

Figure S1: Schematic representation of the incubation experiment design.

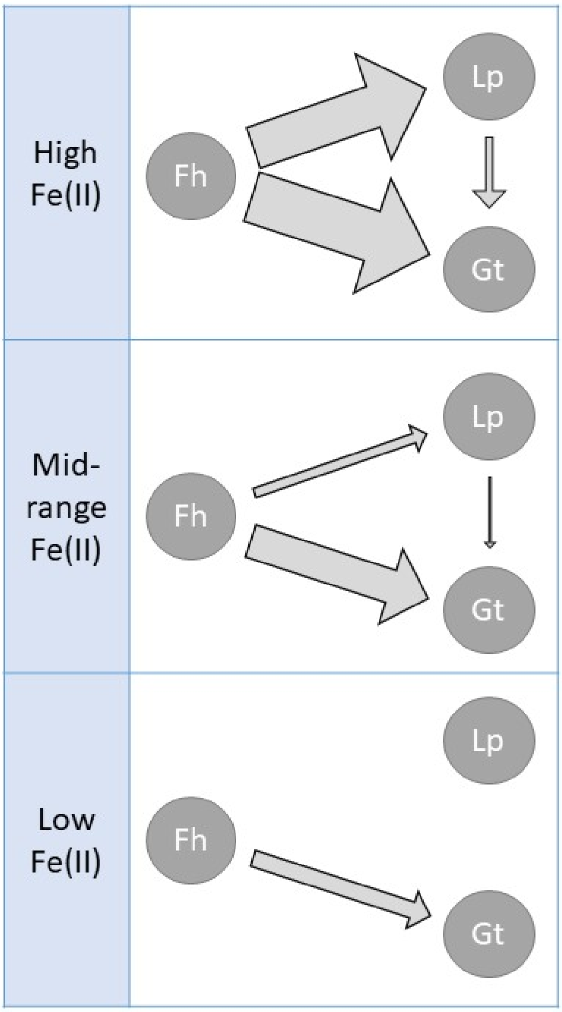

Figure S2: Conceptual diagram of mineral transformation processes occurring in the mesh bags under the influence of varying Fe(II) concentrations in pore water. Arrow widths represent relative rate (not drawn to scale) of the transformation reaction between the indicated pools of ferrihydrite (Fh), goethite (Gt) and lepidocrocite (Lp).

3. Additional pore water analysis

Table S3: Pore water concentration data presented in Figure 1. Measurements were made by in situ probe measurement ([a]), DIMATOC carbon analyser ([b]), ICP-OES ([c]) or by ion chromatography ([d]). Columns headed with 1, 2 or 3 under each element denote measurements of samples from replicate microcosms, and dashes denotes measurements that were not taken. Data points below the limit of determination (LoD) are presented in the table as ≤LoD. The $LoD = 3\frac{SD_y}{m}$, where SD_y is the standard deviation of the y intercepts of the regression line through the calibration standards and m is the gradient of the regression.

Soil	Week	Eh (mV) [a]		pH [a]			DOC (mg L^{-1}) [b]		Fe (mM) [c]		SO_4^{2-} (mM) [d]		Mn (mM) [c]	
		1	2	1	2	3	1	2	1	2	1	2	1	2
PT-T	0	260	267	4.26	4.25	4.25	224	300	≤0.003	≤0.003	16.6	16.9	0.05	0.05
	1	69	88	5.40	5.81	-	163	188	5.98	6.23	15.6	22.5	0.06	0.06
	2	-131	-23	6.44	6.21	-	192	219	9.90	10.6	17.8	19.8	0.06	0.06
	4	-127	-90	6.92	6.62	-	-	-	-	-	-	-	-	-
	6	-203	-143	6.87	6.70	-	126	149	8.15	8.81	9.95	10.8	0.04	0.05
	12	-199	-163	6.82	6.76	-	113	104	3.06	3.13	1.39	1.19	0.02	0.02
PT-S	0	592	592	3.48	3.48	3.45	28.5	35.7	0.005	0.004	1.75	2.52	0.03	0.03
	1	540	541	3.61	3.67	-	20.9	22.8	0.063	0.056	1.33	2.39	0.04	0.03
	2	427	401	3.71	3.7	-	11.7	15.6	0.477	0.461	0.87	2.03	0.03	0.03
	4	333	390	4.22	4.31	-	-	-	-	-	-	-	-	-
	6	74	193	4.12	4.11	-	15.1	17.2	0.745	0.905	1.52	1.37	0.03	0.02
	12	160	54	3.81	3.85	-	11.4	9.26	0.917	0.971	0.924	0.767	0.03	0.03
UB	0	432	362	3.77	3.84	3.8	215	197	0.137	0.125	0.483	0.452	0.84	0.75
	1	16	-11	6.42	6.57	-	168	219	8.36	8.60	0.929	2.65	0.43	0.45
	2	-78	-213	7.28	7.34	-	174	216	9.44	9.65	1.326	1.33	0.36	0.38
	4	-168	-155	7.36	7.37	-	-	-	-	-	-	-	-	-
	6	-165	-210	7.25	7.33	-	127	125	6.58	5.85	≤0.001	<0.001	0.21	0.20
	12	-190	-224	7.10	7.11	-	124	149	5.20	5.78	≤0.001	≤0.001	0.15	0.15
BD	0	368	325	6.07	6.03	6.05	248	508	≤0.003	≤0.003	3.50	6.73	0.02	0.03
	1	-103	-90	6.65	6.76	-	422	537	0.739	1.01	3.24	5.49	0.31	0.36
	2	-71	-112	7.07	6.97	-	493	311	2.06	1.78	0.014	≤0.001	0.39	0.30
	4	-242	-232	7.11	7.17	-	-	-	-	-	-	-	-	-
	6	-275	-139	7.13	6.99	-	317	284	1.01	1.01	≤0.001	0.020	0.22	0.22
	12	-244	-228	7.13	7.01	-	180	177	0.500	0.505	≤0.001	≤0.001	0.13	0.13
CS	0	404	327	4.81	4.83	4.82	246	176	≤0.003	≤0.003	1.175	0.904	0.07	0.07
	1	-139	-146	6.46	6.67	-	159	200	3.25	3.64	≤0.001	0.832	0.09	0.10
	2	-188	-196	6.74	6.88	-	206	231	4.89	5.03	≤0.001	≤0.001	0.11	0.11
	4	-148	-195	7.19	7.19	-	-	-	-	-	-	-	-	-
	6	-202	-273	7.05	6.84	-	159	183	3.90	3.71	≤0.001	0.002	0.09	0.08
	12	-236	-211	6.87	6.88	-	157	181	2.76	3.09	≤0.001	0.002	0.07	0.07

Table S4: Additional pore water concentration data (not presented in Figure 1 and Table S3). Measurements were made by ICP-OES ([a]), by ion chromatography ([b]) or by UV-VIS spectrophotometer ([c]). Columns headed with 1 or 2 under each element refer to measurements of samples from duplicate microcosms. Data points below the limit of determination (LoD) are presented in the table as ≤LoD. The $LoD = 3\frac{SD_y}{m}$, where SD_y is the standard deviation of the y intercepts of the regression line through the calibration standards and m is the gradient of the regression.

Soil	Week	Cl (mM) [b]		Fe(II) (mM) [c]		Total Fe (mM) [c]		K (mM) [a]		Mg (mM) [a]		Na (mM) [a]		P (nM) [a]		S (mM) [a]		Si (mM) [a]	
		1	2	1	2	1	2	1	2	1	2	1	2	1	2	1	2	1	2
PT-T	0	11.5	11.9	0.03	0.03	≤0.01	≤0.01	0.54	0.56	4.40	4.73	11.7	14.8	≤10	≤10	17.1	17.7	0.78	0.78
	1	11.5	13.5	6.15	6.36	5.97	6.00	0.54	0.69	4.98	4.90	8.40	8.61	6.8	9.4	21.0	21.3	0.50	0.46
	2	12.2	12.3	10.1	10.6	9.77	10.8	1.43	0.38	4.77	5.02	6.44	6.87	6.5	11	19.4	20.6	0.28	0.28
	6	13.3	13.3	8.24	9.32	7.89	8.69	0.46	0.36	4.16	4.44	6.18	5.79	3.9	5.5	11.3	14.0	0.32	0.32
	12	14.3	14.5	3.15	3.24	3.14	3.19	0.41	0.59	2.14	2.18	4.39	4.57	≤5	≤5	1.6	1.0	0.36	0.36
PT-S	0	10.1	11.7	0.03	0.03	≤0.01	0.02	0.79	0.77	1.93	2.02	9.17	15.7	≤10	≤10	1.8	2.5	1.00	1.17
	1	10.9	11.3	0.05	0.05	0.04	0.05	1.15	1.02	2.43	2.35	5.00	4.78	≤2	≤2	2.1	2.0	1.64	1.50
	2	11.6	11.5	0.47	0.46	0.45	0.44	0.84	0.79	2.26	2.35	4.22	4.48	≤2	≤2	1.8	2.2	1.46	1.60
	6	12.2	11.7	0.73	0.90	0.71	0.85	0.82	0.61	2.14	1.85	3.74	3.65	≤2	≤2	1.4	1.2	1.46	1.39
	12	11.9	11.7	0.93	0.99	0.89	1.01	0.90	0.90	2.02	1.89	3.65	3.48	≤5	≤5	1.0	0.8	1.28	1.21
UB	0	10.4	10.0	0.14	0.32	0.07	0.15	0.18	0.10	0.70	0.66	5.24	2.28	≤10	≤10	0.5	0.4	0.53	0.46
	1	8.8	11.5	7.92	8.49	8.10	8.26	0.74	0.31	0.58	0.58	1.09	1.04	10	13	2.5	2.3	0.93	0.93
	2	12.2	12.0	9.28	9.96	9.18	10.1	0.26	0.28	0.53	0.53	0.74	0.74	9.0	9.4	1.4	1.4	0.64	0.68
	6	11.7	11.8	6.63	5.82	6.40	5.66	0.26	0.10	0.37	0.37	0.61	0.61	7.7	10	0.2	0.2	0.53	0.50
	12	11.9	11.5	5.29	5.91	5.23	5.57	0.28	0.36	0.33	0.37	0.57	0.52	11	13	≤0.1	≤0.1	0.64	0.68
BD	0	9.6	11.2	0.03	0.03	0.31	≤0.01	0.21	0.31	2.43	4.61	7.86	6.99	11	24	3.5	6.7	0.39	0.43
	1	11.7	11.8	0.37	0.97	0.68	1.02	0.66	0.41	5.39	5.88	1.83	2.09	36	39	3.6	4.6	1.21	1.25
	2	11.5	≤0.002	1.94	1.68	1.97	1.76	0.46	0.15	6.87	4.85	2.04	1.39	100	96	0.8	0.5	1.10	1.10
	6	≤0.002	11.0	0.97	1.00	1.13	1.00	0.20	0.13	4.69	4.40	1.39	1.35	93	96	0.5	0.4	0.82	0.85
	12	12.6	12.1	0.61	0.59	0.51	0.60	0.20	0.26	2.88	2.88	1.09	1.09	82	81	0.2	0.3	0.78	0.82
CS	0	10.3	10.1	0.10	0.06	≤0.01	≤0.01	0.33	0.29	0.74	0.66	6.11	4.80	26	17	1.3	1.0	0.18	0.14
	1	≤0.002	11.3	2.87	3.55	3.10	3.48	0.43	0.41	0.66	0.74	0.48	0.52	64	75	0.7	0.8	0.21	0.21
	2	≤0.002	11.2	4.82	4.95	5.09	4.95	0.41	0.69	0.86	0.95	0.57	0.61	112	115	0.4	0.4	0.28	0.28
	6	11.6	12.0	3.71	3.75	3.75	3.58	0.26	0.28	0.74	0.70	0.48	0.43	84	91	0.3	0.2	0.28	0.28
	12	11.8	12.5	2.83	3.15	2.72	3.05	0.36	0.51	0.66	0.74	0.35	0.61	60	61	≤0.1	0.2	0.36	0.36

4. Elemental content of mineral layers in mesh bags

Table S5: Element concentrations in mineral material that was extracted from the interior (inner and outer core) and untransformed exterior (mostly rim) of two-week-reacted mesh bags. All concentrations are expressed in mg of element per g of dissolved mineral. Relative difference is the percentage increase of concentration in the sample from the exterior compared to the sample from the interior. Data points below the limit of determination (LoD) are presented in the table as ≤LoD. The $LoD = 3\frac{SD_y}{m}$, where SD_y is the standard deviation of the y intercepts of the regression line through the calibration standards and m is the gradient of the regression. Limits of detection vary for the same element because all data are normalised to the initial mass of dissolved mineral.

		Al	As	Ca	Fe	Mn	P	Pb	S	Si	Zn
PT_T	exterior	1.32	≤0.010	7.30	617	0.180	≤0.009	0.703	20.5	9.37	0.054
	interior	0.276	≤0.008	4.33	608	0.262	≤0.006	0.630	14.6	4.08	0.079
	rel. diff.	381		69	1	-31		12	41	130	-31
UB	exterior	0.072	≤0.005	2.52	534	0.493	≤0.004	0.845	4.01	7.30	0.105
	interior	≤0.013	≤0.006	1.06	622	0.435	≤0.005	0.848	4.57	3.81	0.130
	rel. diff.	≥5.54		138	-14	13		0	-12	92	-20
BD	exterior	1.52	0.226	14.8	529	0.827	0.620	0.571	1.09	8.48	0.124
	interior	≤0.011	0.118	11.8	583	0.658	0.141	0.490	4.15	3.90	0.077
	rel. diff.	≥138	93	26	-9	26	339	17	-74	117	61
CS	exterior	1.11	0.307	5.24	537	0.266	1.420	0.553	1.82	4.69	0.105
	interior	0.302	≤0.011	4.07	644	0.201	≤0.009	0.429	3.57	3.31	0.088
	rel. diff.	269	≥27.9	29	-17	32	≥158	29	-49	41	19

5. Photographs of mineral sampling at week twelve

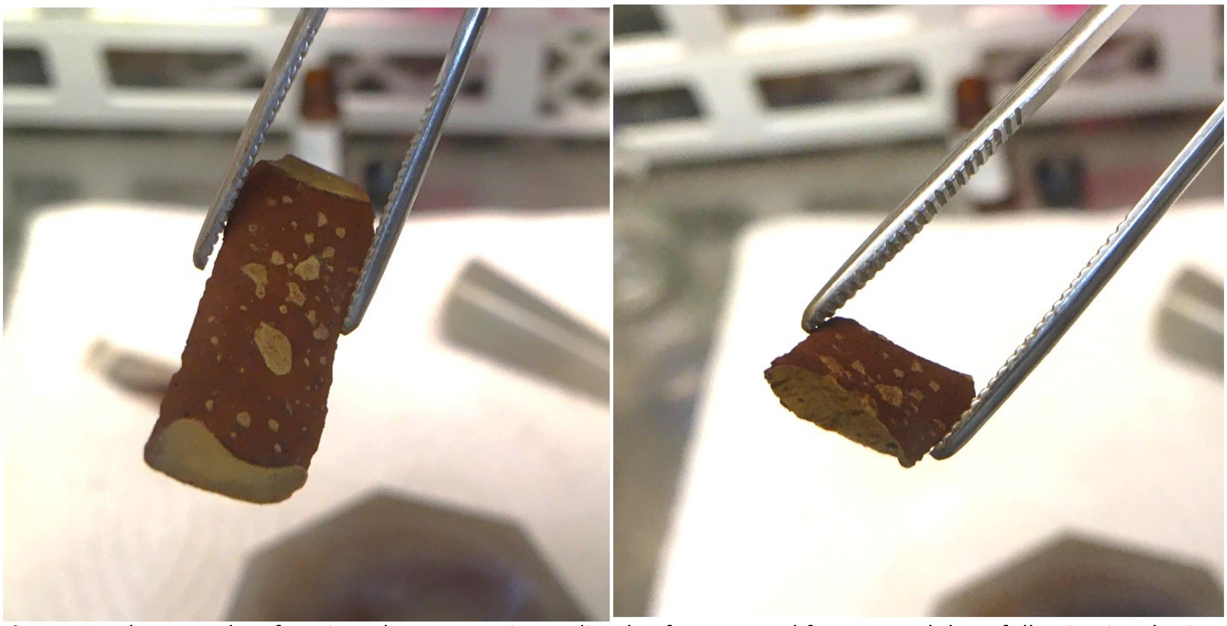

Figure S3: Photographs of a mineral aggregate immediately after removal from a mesh bag, following incubation in CS soil for twelve weeks. On the left, the whole aggregate is presented, and on the right, it is possible to see the cross section, indicating that yellow mineral transformation products were present throughout the cross-section. The orange-brown rim is visible as a covering on most of the mineral aggregate.

6. XRD patterns

Powder X-ray diffraction measurements were performed on dried, crushed and homogenised mineral samples from each soil microcosm. Samples were resuspended in ethanol, transferred onto polished Si wafers without XRD background (711 cut, Sil'tronix Silicon Technologies, France), allowed to dry in place and closed under anoxic seal. Anoxic conditions are maintained for up to 3.5 hours, during which time the sample is measured between 5° and 70° 2θ with a step size of 0.02° 2θ for 4 s at each step (X5 Advance, Bruker, USA). Initial ferrihydrite wafers were prepared for measurement in the same way, but not closed under anoxic seal, and measured between 10° and 70° 2θ with a step size of 0.02° 2θ for 10 s at each step. Soil CS and BD were measured on mechanically milled soil which was gently packed into a holder, and measured between 5° and 70° 2θ with a step size of 0.02° 2θ for 6 s at each step. A knife was used in all measurements to reduce the detection of scattered radiation at low goniometer angles. Samples were analysed in Bragg–Brentano geometry using Cu Kα1 and Cu K α2 radiation (λ = 1.5418 Å, 40 kV, and 40 mA) and a high-resolution energy-dispersive 1D detector (LYNXEYE).

Rietveld quantitative phase analysis (QPA) was performed on TOPAS software (Version 5, Bruker, USA). Ferrihydrite was quantified using PONKCS [3] phase calibration of the synthetic ferrihydrite minerals that were used in the experiment.[4] The XRD pattern of the initial ferrihydrite samples are included in Figures S5 – S9. The PONKCS calibration was carried out on mixtures of 50% (w/w) ferrihydrite and 50% (w/w) corundum (measured in the same way as the pure ferrihydrite samples described above), to obtain an empirical description of the ferrihydrite diffraction pattern as a mass-calibrated hkl-phase. Ferrihydrite from the same synthesis batch was used in the experiments that finished at weeks one, two and six. Ferrihydrite from a second batch was used in microcosms that were sampled at the twelve-week timepoint. Other phases were only fit if they could be attributed to crystalline peaks that were observed in the diffraction patterns, and if their fitted area made up more than 1% of the total mineral abundance in the sample (except for lepidocrocite which produces a very strong characteristic (0 2 0) peak and could be confidently identified with abundance of 0.5%). Crystalline phases were fit using published structure files of goethite (International Crystal Structure Database (ICSD) no. 239321, accessed 22.2.2019) [5], lepidocrocite (ICSD no. 93948, accessed 20.4.2017),[6] quartz (American Crystal Structure Database (AMCSD), no. 0000789, accessed 13.4.2016),[7] rhodocrosite (ICSD no. 80867, accessed

18.4.2017),[8] and kaolinite (ICSD no. 107377, accessed 5.5.2017).[9] Preferred orientation was considered for goethite (0 2 1 and 1 1 0) and lepidocrocite (0 2 0) and implemented in Topas by application of the March-Dollase equation. Previous investigations of mixtures of ferrihydrite and crystalline Fe (oxyhydr)oxide minerals have produced estimates of ferrihydrite abundance that are accurate to within ~10% (absolute error).[4, 10, 11] Estimates are most accurate when fractional abundance of ferrihydrite is higher.[4, 12] Small quantities of poorly crystalline minerals such as mackinawite may not have been detected by this method.

The crystallite size of crystalline products of ferrihydrite transformation was estimated in TOPAS by calculation of the volume weighted size parameter (LVol-IB) with combined Lorentzian and Gaussian curve fitting models. The models were fit to whole spectra, and therefore average the crystallite size across all crystal diffraction orientations.

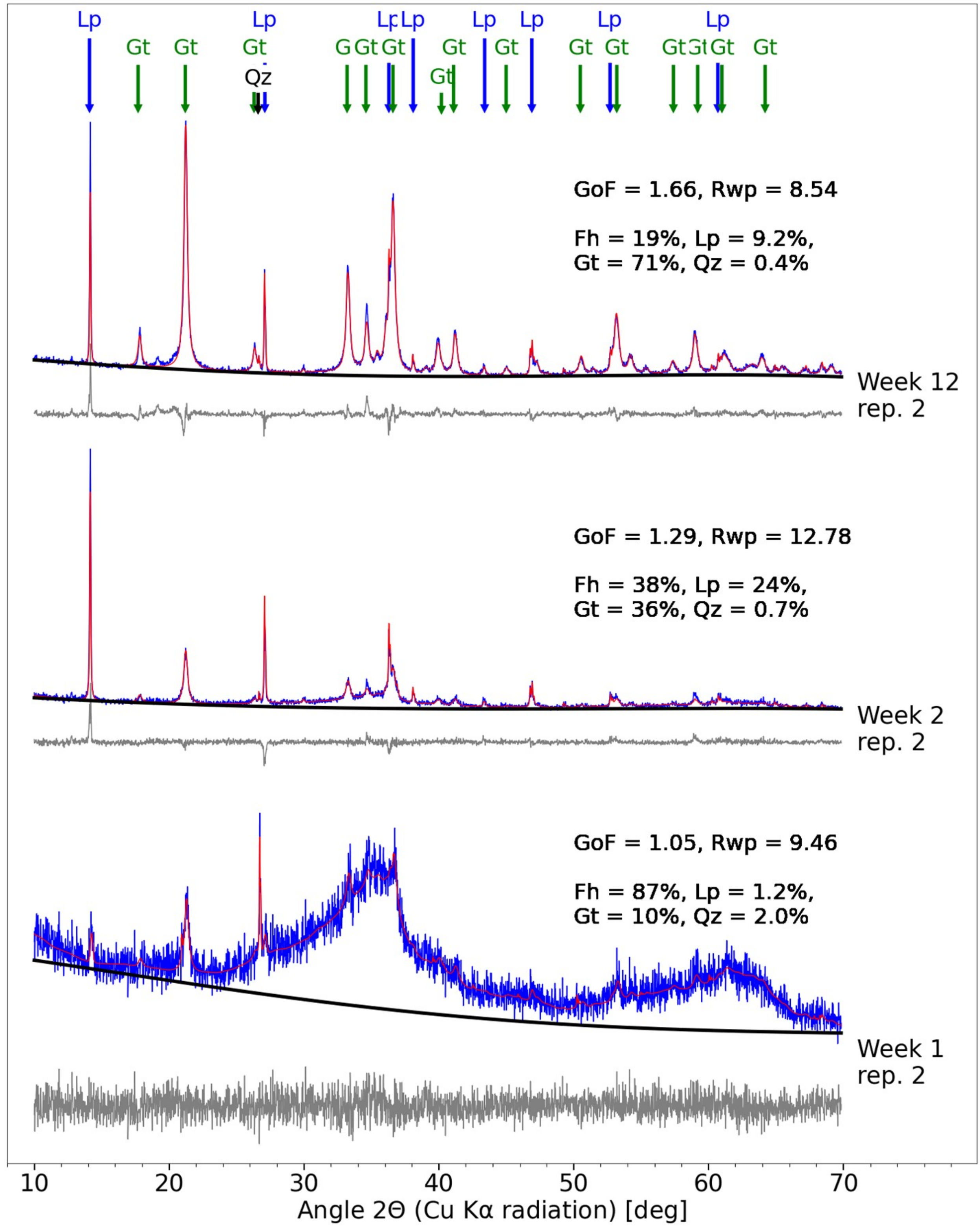

Figure S4: Example Rietveld fitting for three XRD patterns from PT-T soil. The blue curve is the plot of the measured data, the red is the fitted model, the bold black line is the fitted background scatter, and the grey plot indicates the residual. All plots are normalised to the range of the respective raw data. Major diffraction peaks are assigned to minerals used in the Rietveld fitting (Lp for lepidocrocite, Gt for goethite and Qz for quartz), excluding ferrihydrite (Fh), and the QPA results are reported next to the plots to two significant figures. 'GoF refers to the goodness of fit of the model (see Table S6 for explanation and comparison). The labels 'rep. 1' and 'rep. 2' denote results from mesh bags that were incubated in replicate microcosms.

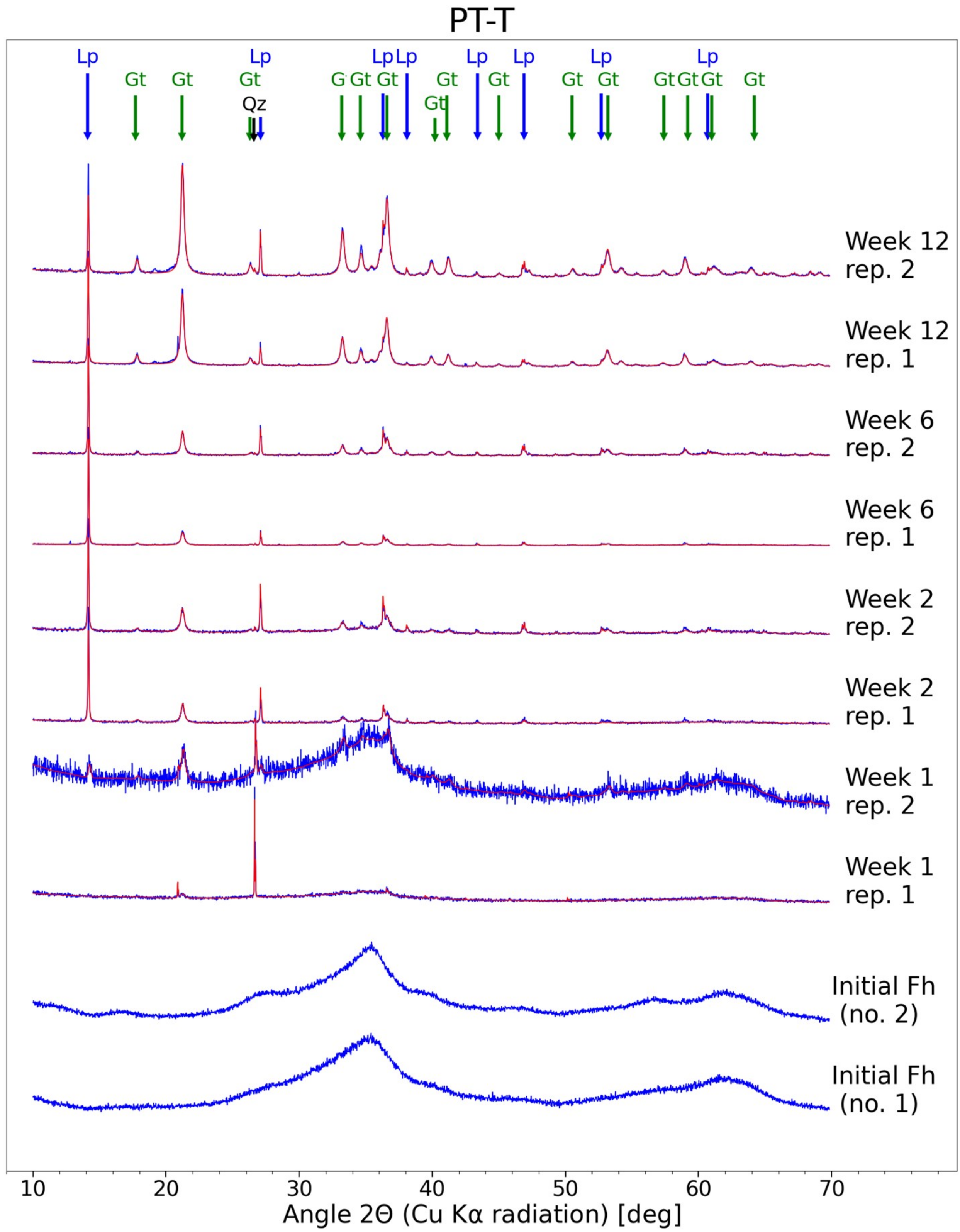

Figure S5: XRD patterns of minerals in mesh bags from PT-T soil microcosms (including those plotted with more details of the Rietveld fitting in Figure S3). Raw data is plotted in blue and the Rietveld fit (total of all modelled phases) is plotted in red. The intensities of all spectra are normalised by the range. 'Initial Fh (no. 1)' refers to the starting material for microcosms that was removed from soil after one, two and six weeks and 'initial Fh (no. 2)' for material removed from soil after twelve weeks. 'Rep. 1' and 'rep. 2' denote results from mesh bags that were incubated in replicate microcosms. Major diffraction peaks are identified above the plots, excluding ferrihydrite.

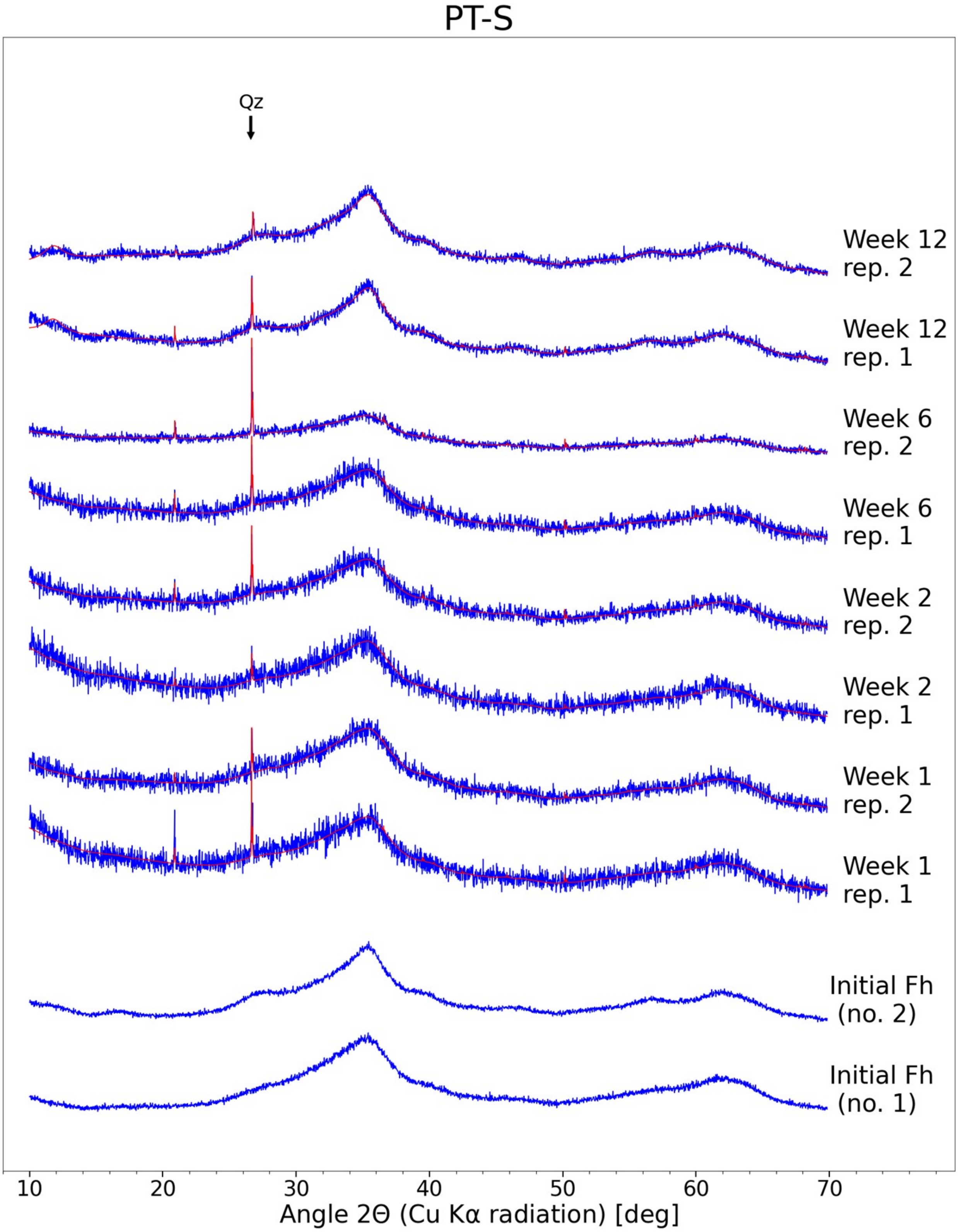

Figure S6: XRD patterns of minerals in mesh bags from PT-T soil microcosms (including those plotted with more details of the Rietveld fitting in Figure S3). Raw data is plotted in blue and the Rietveld fit (total of all modelled phases) is plotted in red. The intensities of all spectra are normalised by the range. 'Initial Fh (no. 1)' refers to the starting material for microcosms that was removed from soil after one, two and six weeks and 'initial Fh (no. 2)' for material removed from soil after twelve weeks. 'Rep. 1' and 'rep. 2' denote results from mesh bags that were incubated in replicate microcosms. Major diffraction peaks are identified above the plots, excluding ferrihydrite.

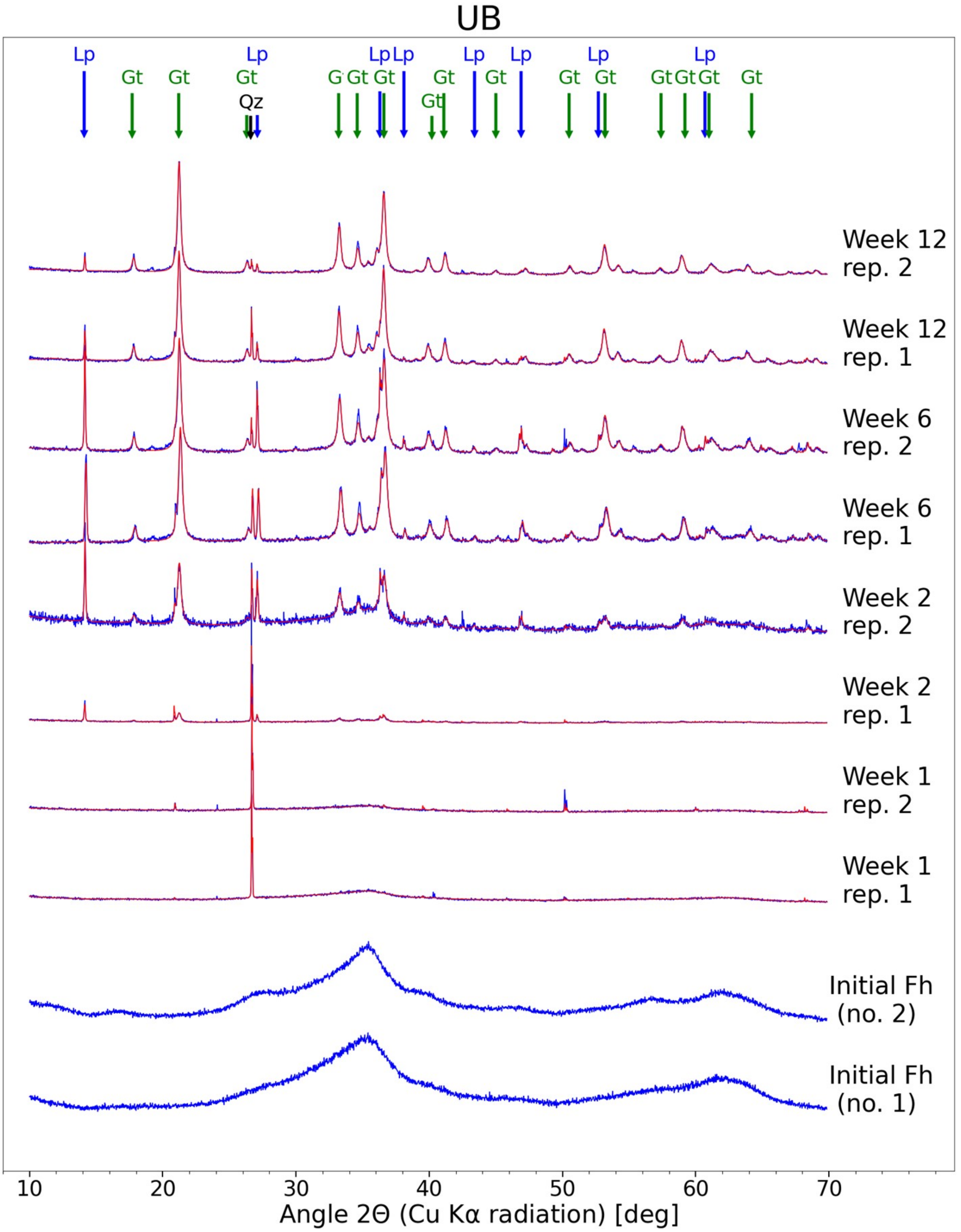

Figure S7: XRD patterns of minerals in mesh bags from PT-T soil microcosms (including those plotted with more details of the Rietveld fitting in Figure S3). Raw data is plotted in blue and the Rietveld fit (total of all modelled phases) is plotted in red. The intensities of all spectra are normalised by the range. 'Initial Fh (no. 1)' refers to the starting material for microcosms that was removed from soil after one, two and six weeks and 'initial Fh (no. 2)' for material removed from soil after twelve weeks. 'Rep. 1' and 'rep. 2' denote results from mesh bags that were incubated in replicate microcosms. Major diffraction peaks are identified above the plots, excluding ferrihydrite.

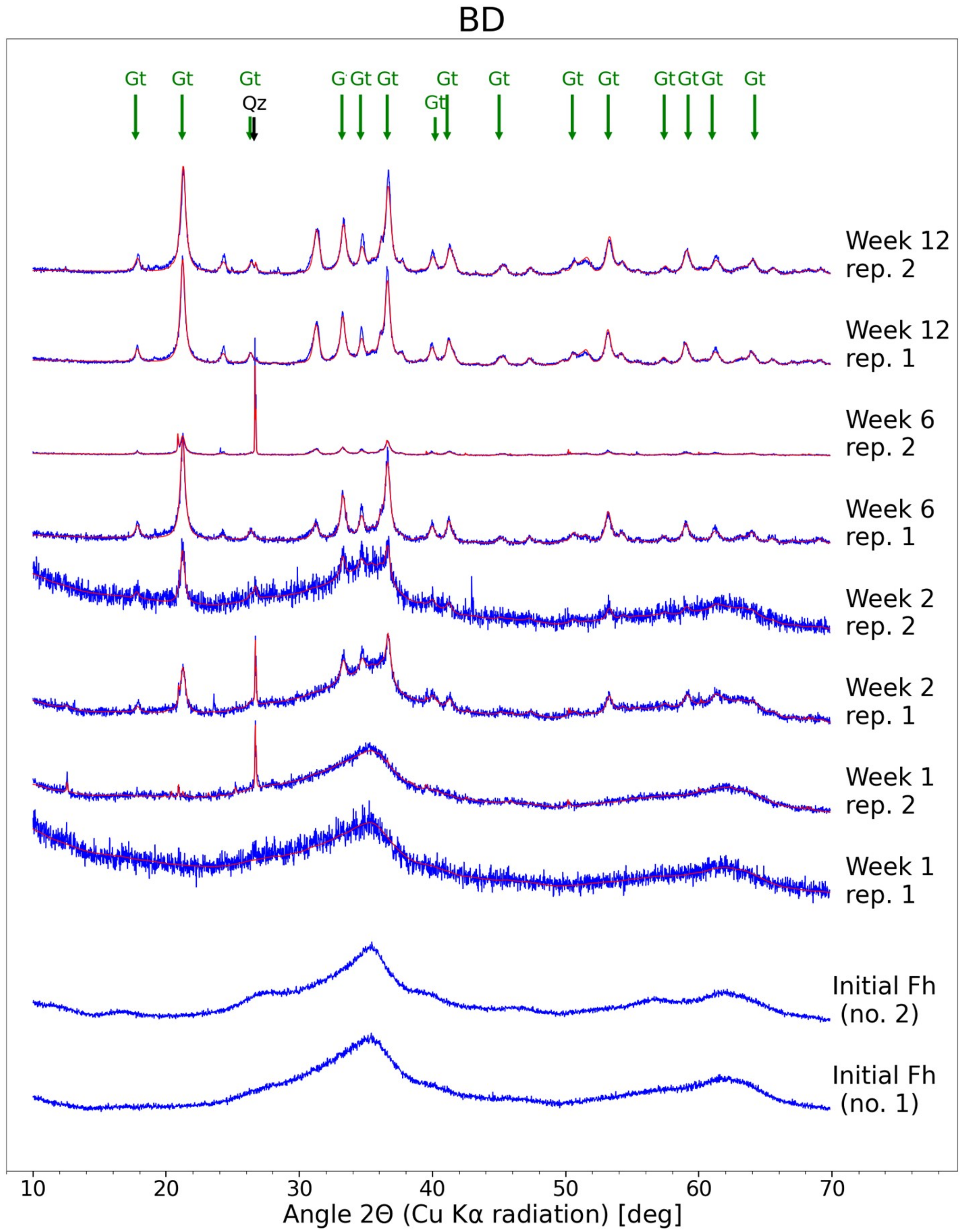

Figure S8: XRD patterns of minerals in mesh bags from PT-T soil microcosms (including those plotted with more details of the Rietveld fitting in Figure S3). Raw data is plotted in blue and the Rietveld fit (total of all modelled phases) is plotted in red. The intensities of all spectra are normalised by the range. 'Initial Fh (no. 1)' refers to the starting material for microcosms that was removed from soil after one, two and six weeks and 'initial Fh (no. 2)' for material removed from soil after twelve weeks. 'Rep. 1' and 'rep. 2' denote results from mesh bags that were incubated in replicate microcosms. Major diffraction peaks are identified above the plots, excluding ferrihydrite.

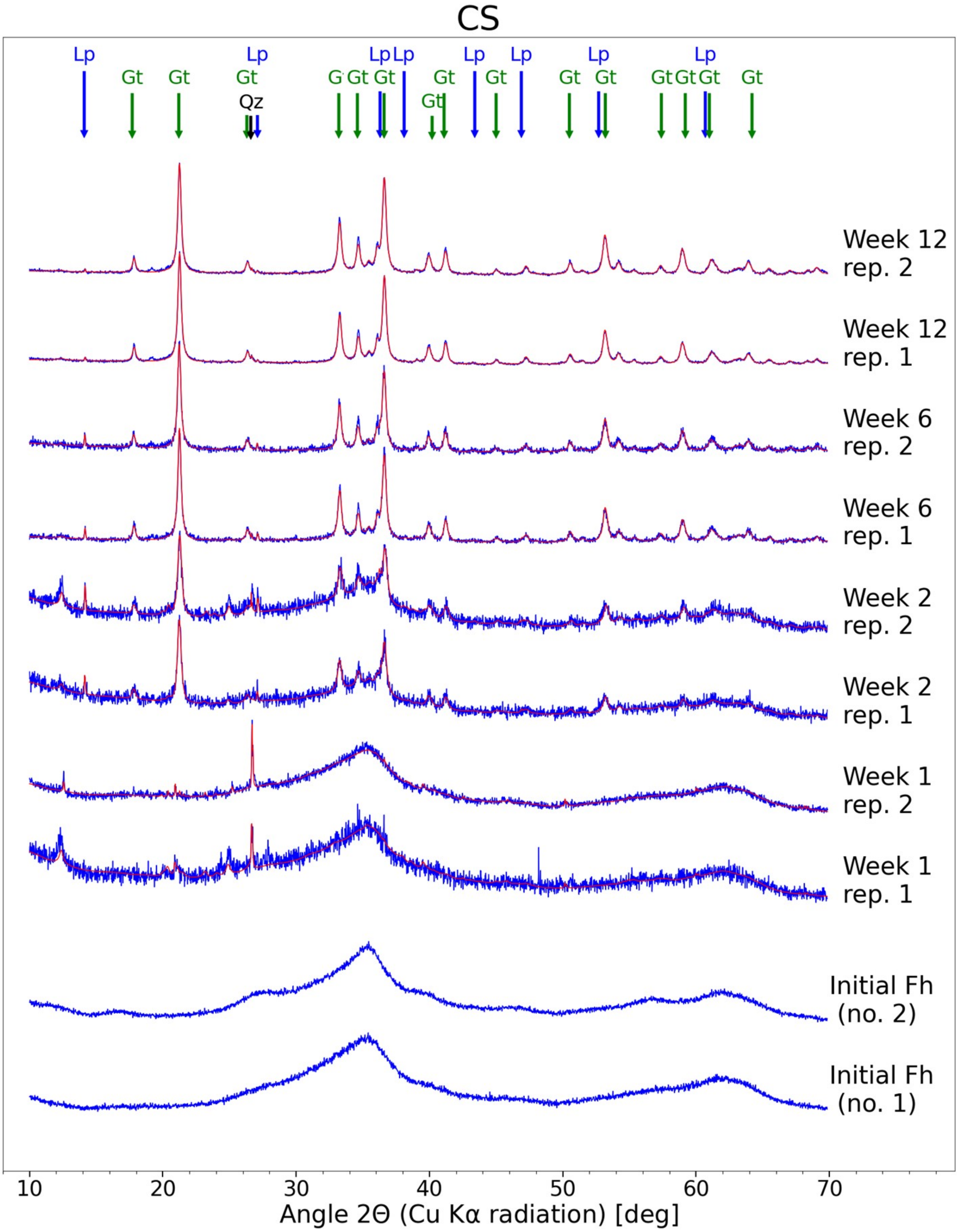

Figure S9: XRD patterns of minerals in mesh bags from PT-T soil microcosms (including those plotted with more details of the Rietveld fitting in Figure S3). Raw data is plotted in blue and the Rietveld fit (total of all modelled phases) is plotted in red. The intensities of all spectra are normalised by the range. 'Initial Fh (no. 1)' refers to the starting material for microcosms that was removed from soil after one, two and six weeks and 'initial Fh (no. 2)' for material removed from soil after twelve weeks. 'Rep. 1' and 'rep. 2' denote results from mesh bags that were incubated in replicate microcosms. Major diffraction peaks are identified above the plots, excluding ferrihydrite.

Table S6: Fractions of minerals detected in bulk mineral sachets by XRD following incubation, expressed as percentages. [a] The Goodness of Fit (GoF) is defined as R_{wp}/R_{exp}, where R_{wp} is the weighted profile R-factor and R_{exp} the expected R-factor. Lower GoF values may indicate better fits. XRD patterns were fit using a PONKCS[3] phase calibration of the ferrihydrite used in the experiment and published structure files of goethite (International Crystal Structure Database (ICSD) no. 239321, accessed 22.2.2019),[5] lepidocrocite (ICSD – 93948, accessed 20.4.2017),[6] quartz (American Crystal Structure Database (amcsd) no. 0000789, accessed 13.4.2016),[7] rhodochrosite (ICSD no. 80867, accessed 18.4.2017) [8] and kaolinite (ICSD no. 107377, accessed 5.5.2017).[9]

**Sample code**	**Week**	**Good-ness of fit[a]**		**Ferrihydrite [%]**			**Goethite [%]**			**Lepidocrocite [%]**			**Rhodochrosite [%]**			**Quartz [%]**			**Kaolonite [%]**		
		**Rep 1**	**Rep 2**	**Rep 1**	**Rep 2**	***Average***	**Rep 1**	**Rep 2**	***Average***	**Rep 1**	**Rep 2**	***Average***	**Rep 1**	**Rep 2**	***Average***	**Rep 1**	**Rep 2**	***Average***	**Rep 1**	**Rep 2**	***Average***
PT-T	1	1.1	1.1	81	87	***84***	6	10	***8***	0	1	***1***	0	0	***0***	13	2	***7***	0	0	***0***
	2	1.4	1.3	26	38	***32***	42	36	***39***	31	24	***28***	0	0	***0***	1	1	***1***	0	0	***0***
	6	1.4	1.2	12	20	***16***	61	55	***58***	26	24	***25***	0	0	***0***	1	1	***1***	0	0	***0***
	12	1.6	1.7	19	19	***19***	73	71	***72***	7	9	***8***	0	0	***0***	0	0	***0***	0	0	***0***
PT-S	1	1.1	1.0	99	100	***100***	0	0	***0***	0	0	***0***	0	0	***0***	1	0	***1***	0	0	***0***
	2	1.1	1.1	100	100	***100***	0	0	***0***	0	0	***0***	0	0	***0***	0	0	***0***	0	0	***0***
	6	1.0	1.1	99	98	***98***	0	0	***0***	0	0	***0***	0	0	***0***	1	2	***2***	0	0	***0***
	12	1.1	1.1	99	100	***99***	0	0	***0***	0	0	***0***	0	0	***0***	1	0	***1***	0	0	***0***
UB	1	1.2	1.3	96	84	***90***	0	0	***0***	0	0	***0***	0	0	***0***	4	16	***10***	0	0	***0***
	2	1.5	1.1	37	52	***45***	26	35	***30***	9	9	***9***	0	0	***0***	28	4	***16***	0	0	***0***
	6	1.4	1.6	15	22	***19***	69	64	***67***	11	11	***11***	0	0	***0***	4	2	***3***	0	0	***0***
	12	1.4	1.5	44	34	***39***	51	64	***58***	2	1	***2***	0	0	***0***	3	1	***2***	0	0	***0***
BD	1	1.1	1.1	100	96	***98***	0	0	***0***	0	0	***0***	0	0	***0***	0	2	***1***	0	2	***1***
	2	1.1	1.0	76	82	***79***	17	19	***18***	0	0	***0***	0	0	***0***	2	1	***2***	3	0	***1***
	6	1.2	1.5	15	13	***14***	77	58	***67***	0	0	***0***	9	9	***9***	0	20	***10***	0	0	***0***
	12	1.7	1.7	15	15	***15***	69	68	***69***	0	0	***0***	16	16	***16***	0	0	***0***	0	1	***0***
CS	1	1.1	1.2	87	86	***87***	0	0	***0***	0	0	***0***	0	0	***0***	2	4	***3***	11	10	***10***
	2	1.0	1.1	63	66	***64***	29	21	***25***	1	1	***1***	0	0	***0***	0	0	***0***	6	12	***9***
	6	1.1	1.1	24	17	***20***	69	80	***74***	1	1	***1***	0	0	***0***	0	0	***0***	6	2	***4***
	12	1.4	1.5	24	21	***22***	71	76	***73***	0	0	***0***	0	0	***0***	0	0	***0***	4	3	***4***

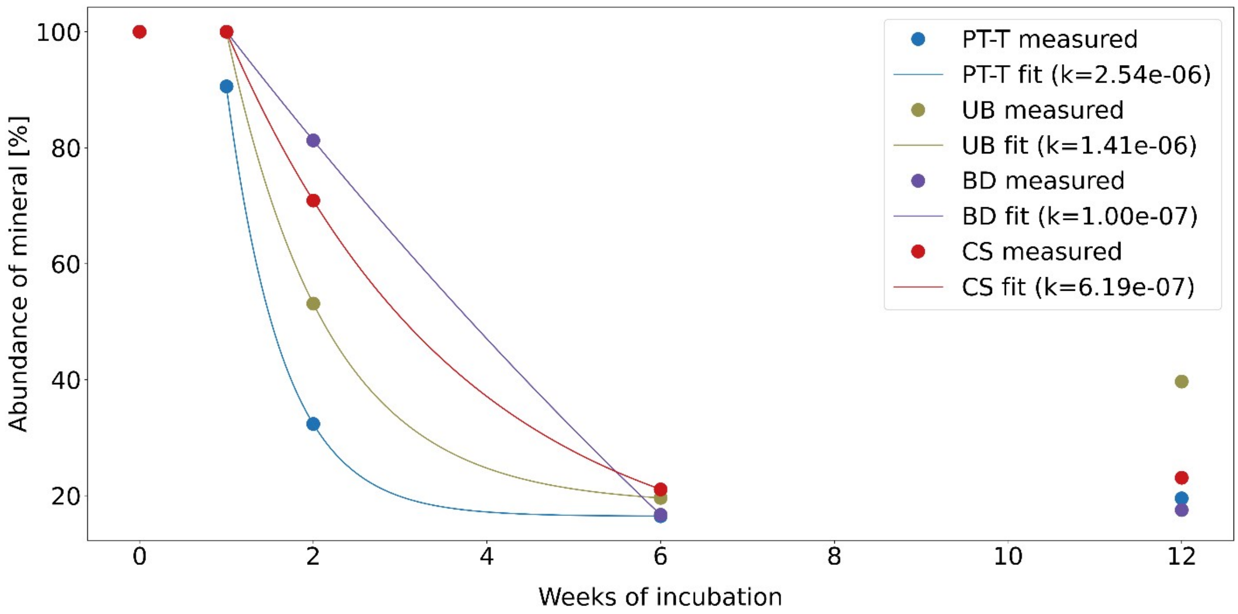

Figure S10: Exponential decay of ferrihydrite in mesh bags, as estimated by Rietveld fitting of XRD patterns, and fit for samples measured after one, two and six weeks. The exponential decay constants are abbreviated as k.

7. Crystallinity of products

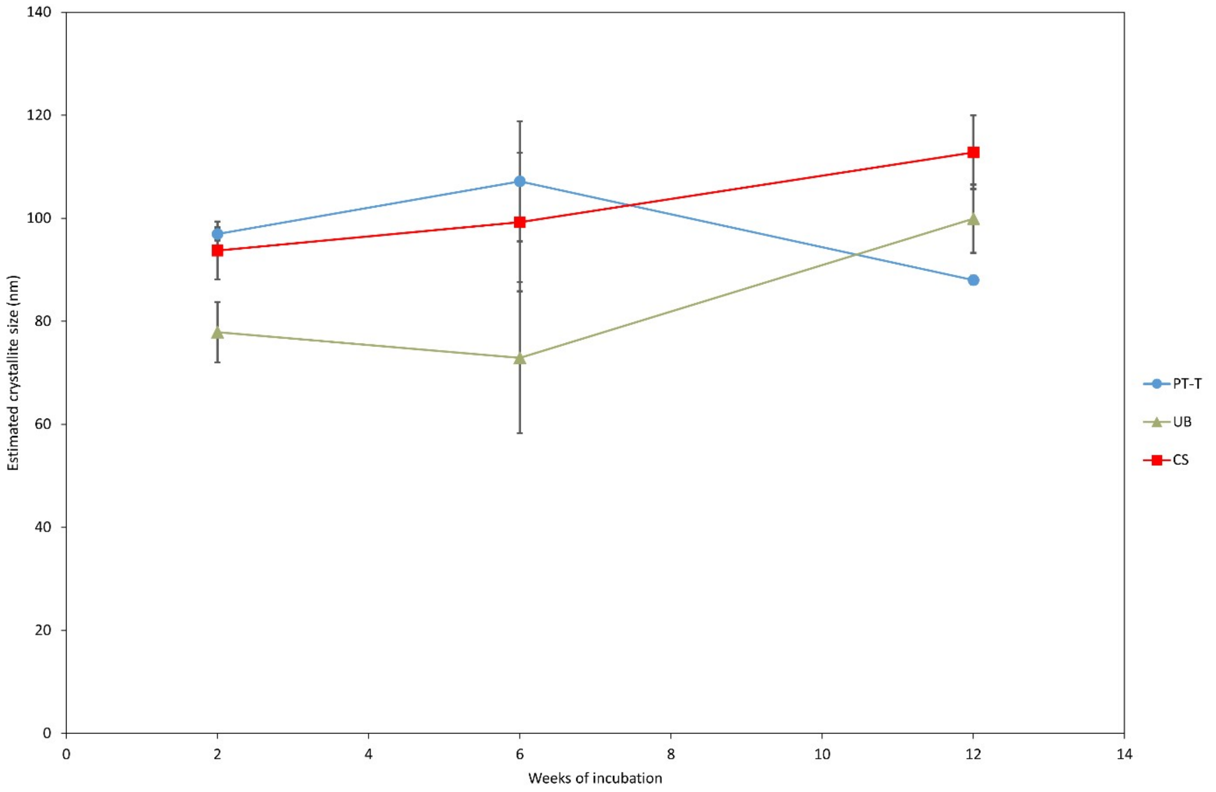

Figure S11: Crystallite size of lepidocrocite (LVol-IB) as estimated from Rietveld fitting of complete XRD spectra. Error bars indicate the range of measurements on samples that were incubated in duplicate microcosms.

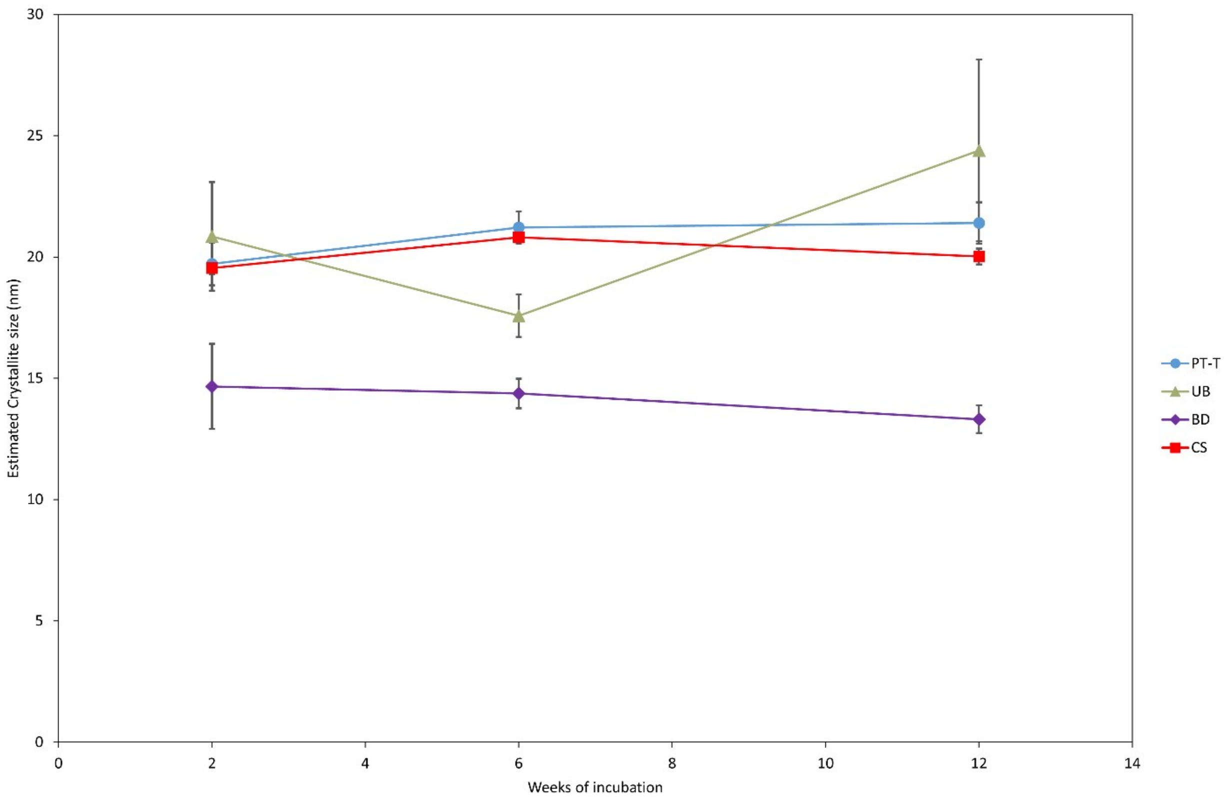

Figure S12: Crystallite size of goethite (LVol-IB) as estimated from Rietveld fitting of complete XRD spectra. Error bars indicate the range of measurements on samples that were incubated in duplicate microcosms.

8. Additional secondary electron (SE) images

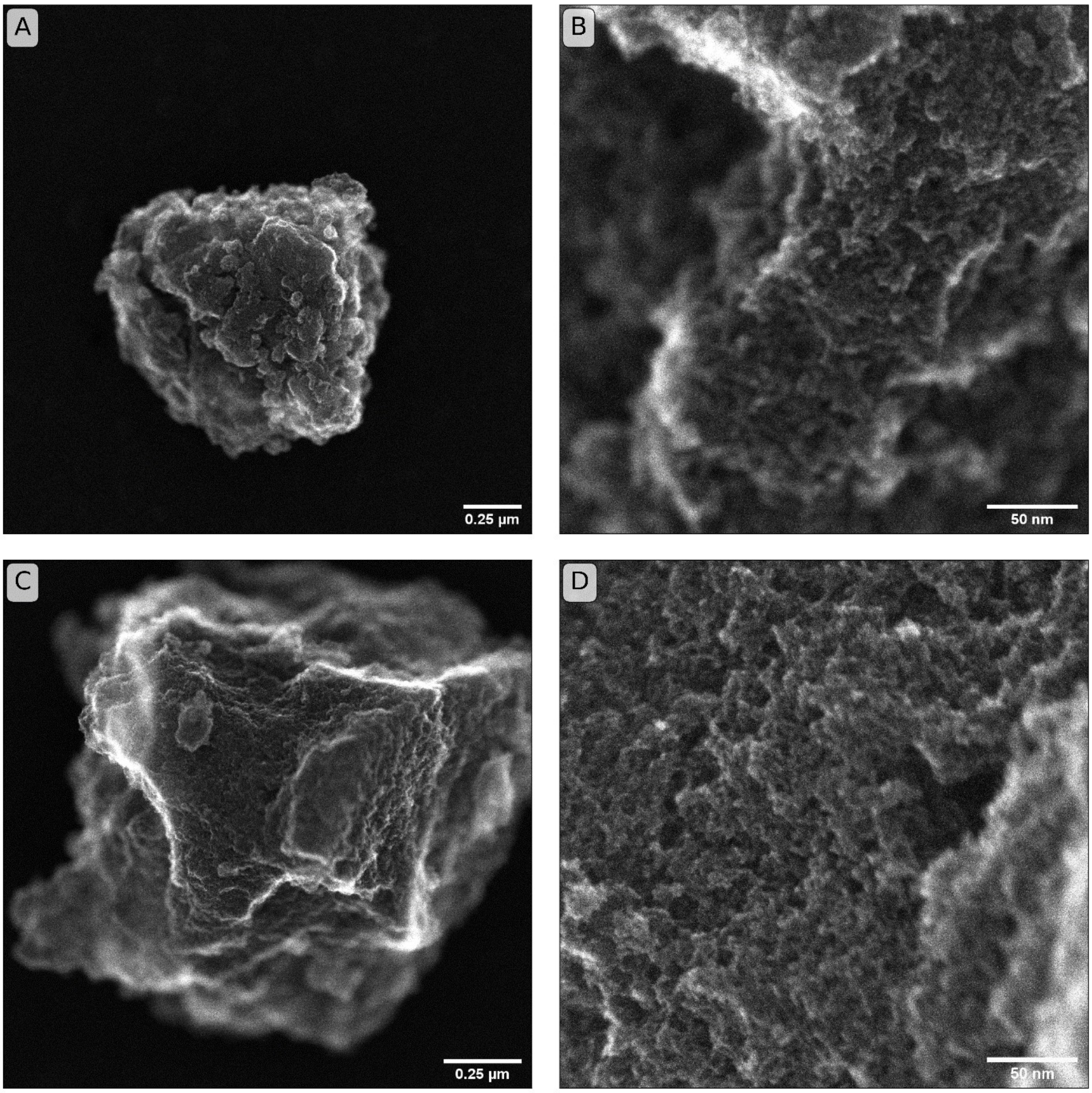

Figure S13: Secondary electron (SE) images of ferrihydrite before the incubation experiments. A and B are images of the ferrihydrite synthesis batch used in microcosms that were sampled after one, two and six weeks for bulk analysis. Images C and D are of the ferrihydrite synthesis batch used in microcosms that were sampled after twelve weeks for bulk analysis, and two weeks for Raman spatial analysis.

9. Additional information about µ-Raman spectroscopy mapping

Reference materials used in Raman study

Reference spectra were the average of at least 1500 spectra, each collected for 8 s from independent locations on the same reference mineral sample, using the same instrument and optical set-up as used for the sample collections. Sample minerals were distributed on silicon wafers, using the same preparation method as used for XRD measurements. The ferrihydrite reference is the starting mineral from this experiment. The lepidocrocite, goethite and hematite reference spectra are measurement of minerals acquired from a commercial source (Bayferrox 943, Bayferrox 910, and Bayferrox 105M, respectively; Bayer, Germany; additional characterisation in ref [13]; Figure 4G and Figure S18). The reference for laser damaged mineral was measured on a section of severely laser-damaged ferrihydrite.

Limitations of Raman spectroscopy in this study

The principle of Raman spectrometry is fundamentally quantitative but numerous factors can affect the quantification process. Firstly, measured Raman spectra can depend on specific features of the crystals or measurement set-up. For example, spectral peak intensity and integrated area can vary according to the specific properties of the crystals being measured and the crystal orientation with regard to the laser light.[14] Secondly, the quantification depends on the numerical methods used to perform the component analysis, and different component analysis algorithms create biases towards certain components in some spectra. Thirdly, the large 'lack-of-fit' component in the component analysis used in this study quantifies parts of the spectra that cannot be identified as being part of one of the input spectra from known minerals in the sample. The 'lack of fit' can be explained by differences in peak intensity between the measured spectra and references due to different measurement conditions of mineral structural properties; background noise due to poor auto-focusing of the laser limitations on laser intensity and exposure time, and other sources of noise that are inherent in Raman spectroscopy;[15] and contaminants such as clay minerals. Lack of fit is mostly associated with minerals containing greater ferrihydrite abundance. It was not possible to control many of the factors that may alter the quantification of crystal phases and it is infeasible to produce calibration standards with similar crystal features to the samples, because the samples are the products of transformation

processes in complex environments. Therefore, the absolute values should be considered semi-quantitative and concentration estimates must be interpreted with care. The experiment is internally consistent, and spatial trends within Raman maps are reliable.

Beam damage caused by the laser used in the Raman spectroscopy is identifiable by the formation of a new mineral phase, which may be associated with faint colour changes. Key peaks of the reference spectrum used to fit beam damage matched a reference hematite spectrum (Figure S18), indicating that high laser intensity transforms ferrihydrite, goethite and/or lepidocrocite to hematite. Since hematite is not identified in bulk XRD measurements, and severe beam damage occurs in regular identifiable patterns, the presence of hematite is attributed to beam damage. Beam damage was minimised by using a low laser power and limiting the time of exposure of the laser on the mineral surface. This must be balanced against the measurement time and laser power required to produce Raman spectra with a sufficient signal-to-noise ratio for reliable spectral analysis. A component of beam-damaged mineral is identified by the component analysis in each of the maps. A small percentage can be assigned to noise in cases where beam damage is fit but characteristic peaks are not identifiable. However, in most cases where beam damage is identified, characteristic peaks of the reference are identifiable in the sample spectrum. Small amounts of beam damage do not hinder the qualitative interpretation of mineral distribution.

Table S7: Summary of mineral abundance estimates from µ-Raman component analyses and lepidocrocite particle statistics from Raman maps presented in Figure 4C, Figure 4E and Figures S14–S46.

Sample identification				Segment analysis			Iron mineral relative abundance estimated by component analysis (%)				Iron mineral relative abundance estimated by XRD QPA of equivalent samples (from Table S6) (%)			
Map scale	Microcosm soil	Description of map location on chunk cross section with reference to zones of mineral transformation	Raman map Figure reference	Lepidocrocite segment density (particles mm^{-2})	Average lepidocrocite segment area (um^2)	Average lepidocrocite segment nearest neighbour distance (µm)	Lepidocrocite abundance	Goethite abundance	Ferrihydrite abundance	Hematite abundance	Lepidocrocite abundance	Goethite abundance	Ferrihydrite abundance	Hematite abundance
Large scale (25 to 35 µm resolution)	BD		Fig S12				0	28	65	7	0	19	81	0
	PT-T		Fig S13				18	43	28	12	28	40	32	0
	CS		Fig 4C				2	42	52	4	1	28	71	0
	UB		Fig 4E				15	28	48	9	11	36	53	0
Small scale (1 µm resolution)	CS	Inner core	Fig 4F.1	1000	2.6	14.4	1	25	70	4				
		Transition zone	Fig 4F.2	3333	1.7	10.2	3	56	36	5				
		Outer core	Fig 4F.3	11261	2.5	5.5	6	55	33	6				
	UB	Inner core	Fig 4F.4	6250	2.4	7.3	4	13	77	6				
		Transition zone	Fig 4F.5	12244	4.6	5.3	9	25	57	9				
		Outer core	Fig 4F.6	16667	7.5	5.0	15	40	29	16				
	PT-T	Upper-middle, intrusion	Fig S33	13703	13.1	5.3	19	41	25	15				
		Upper edge, inside rim	Fig S34	8823	13.0	5.8	11	41	40	8				
		Middle	Fig S35	10476	5.9	5.6	8	53	28	10				
	BD	Middle	Fig S43				1	28	65	7				

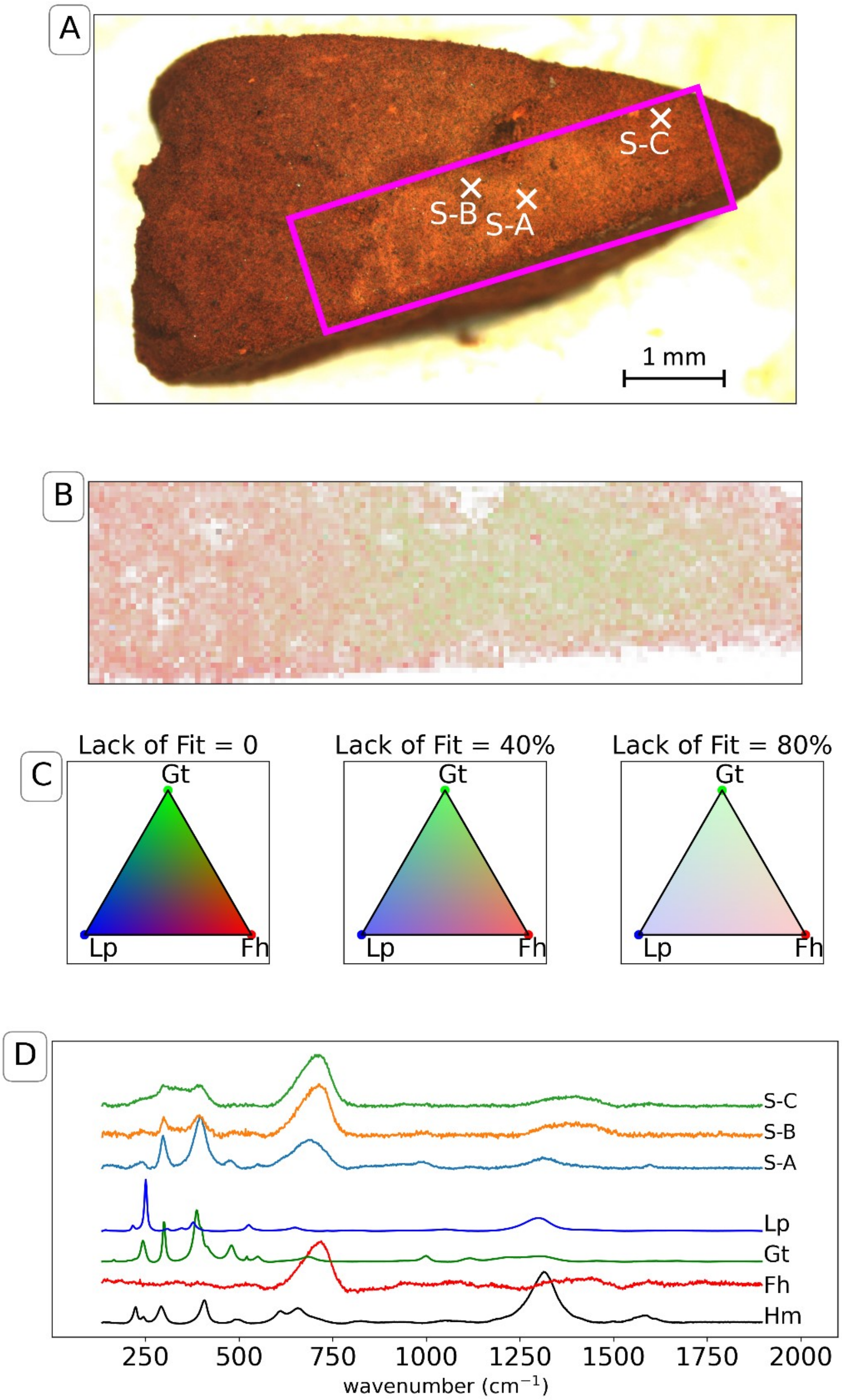

Figure S14: Raman spectroscopy from a cross section of the mineral in a mesh bag from soil BD after two weeks of incubation. A: Microscope image with locations of Raman spectra and spectral map area overlayed in purple. B: Spectral map (resolution 25 µm), analysed with component analysis. Colours are based on a Maxwell colour triangle [16] where the ferrihydrite (Fh), goethite (Gt) and lepidocrocite (Lp) from component analysis are plotted as the red, green and blue colour components, respectively. The transparency of the colours is set according to the lack of fit in the component analysis, with bright colours corresponding with complete fits using the three available components, white corresponding to the calculated lack of fit component, and dark colours corresponding to regions where other minerals included in the component analysis but not displayed on the chart (specifically hematite (Hm) from beam damage. C: Legend for colours in spectral map (panel B). D: Spectra from individual locations indicated by labels in panel A.

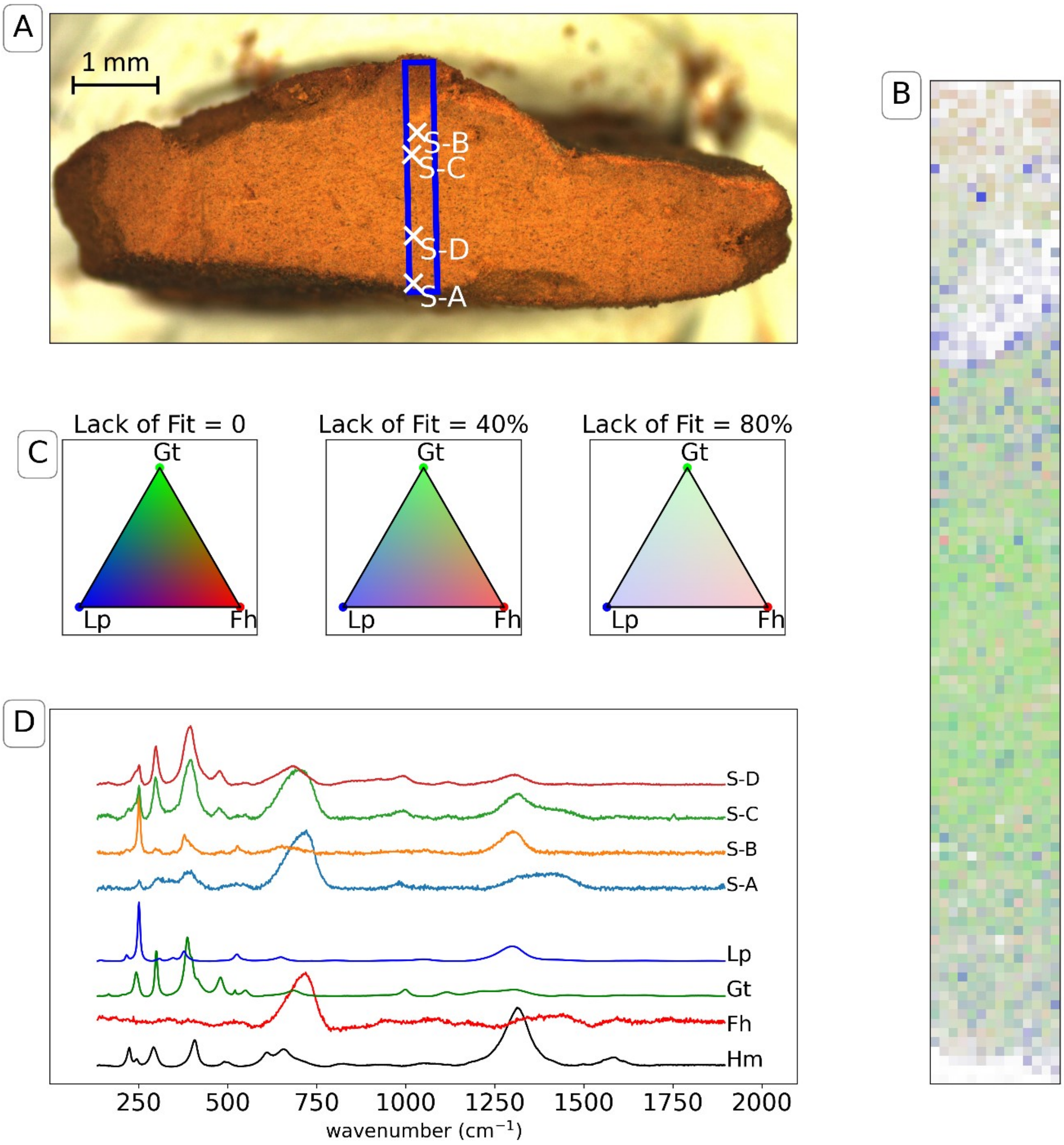

Figure S15: Raman spectroscopy from of a cross section of the mineral in a mesh bag from soil PT-T after two weeks of incubation. A: Microscope image with locations of Raman spectra and spectral map area overlayed in blue. B: Spectral map (resolution 25 µm), analysed with component analysis. Colours are based on a Maxwell colour triangle [16] where the ferrihydrite (Fh), goethite (Gt) and lepidocrocite (Lp) from component analysis are plotted as the red, green and blue colour components, respectively. The transparency of the colours is set according to the lack of fit in the component analysis, with bright colours corresponding with complete fits using the three available components, white corresponding to the calculated lack of fit component, and dark colours corresponding to regions where other minerals included in the component analysis but not displayed on the chart (specifically hematite (Hm) from beam damage. C: Legend for colours in spectral map (panel B). D: Spectra from individual locations indicated by labels in panel A.

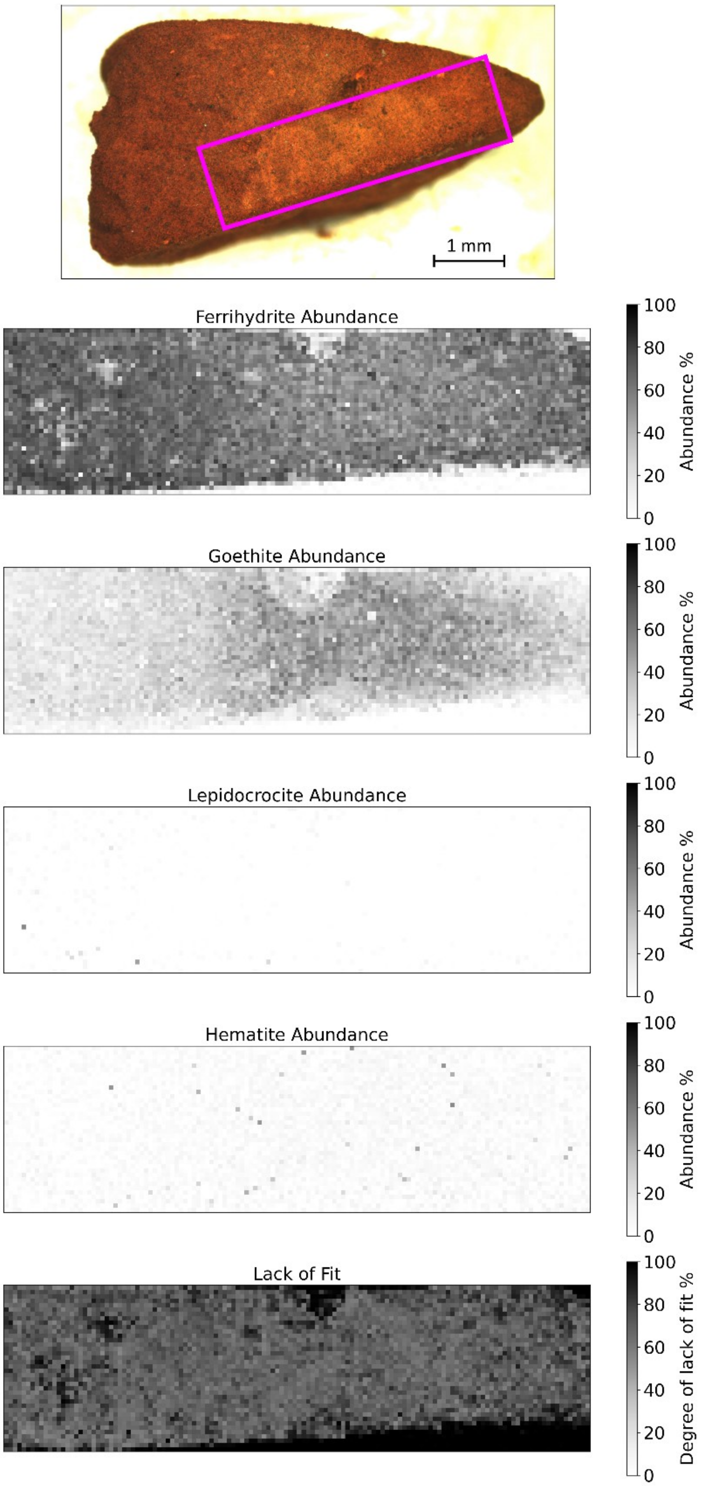

Figure S16: Black and white representation of the Raman map of a mineral aggregate cross section from soil BD as presented in Figure S14.

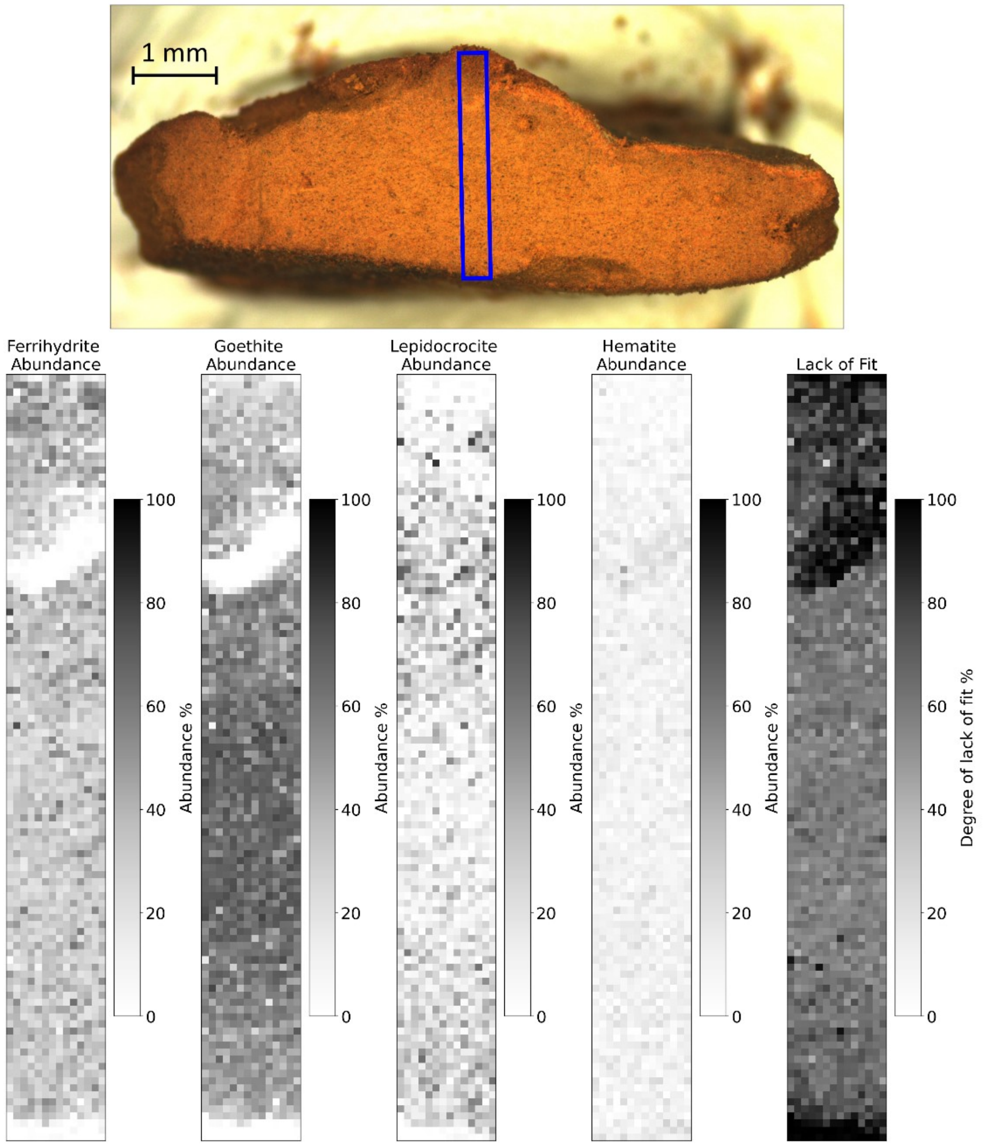

Figure S17: Black and white representation of the Raman map of a mineral aggregate cross section from soil PT-T as presented in Figure S15.

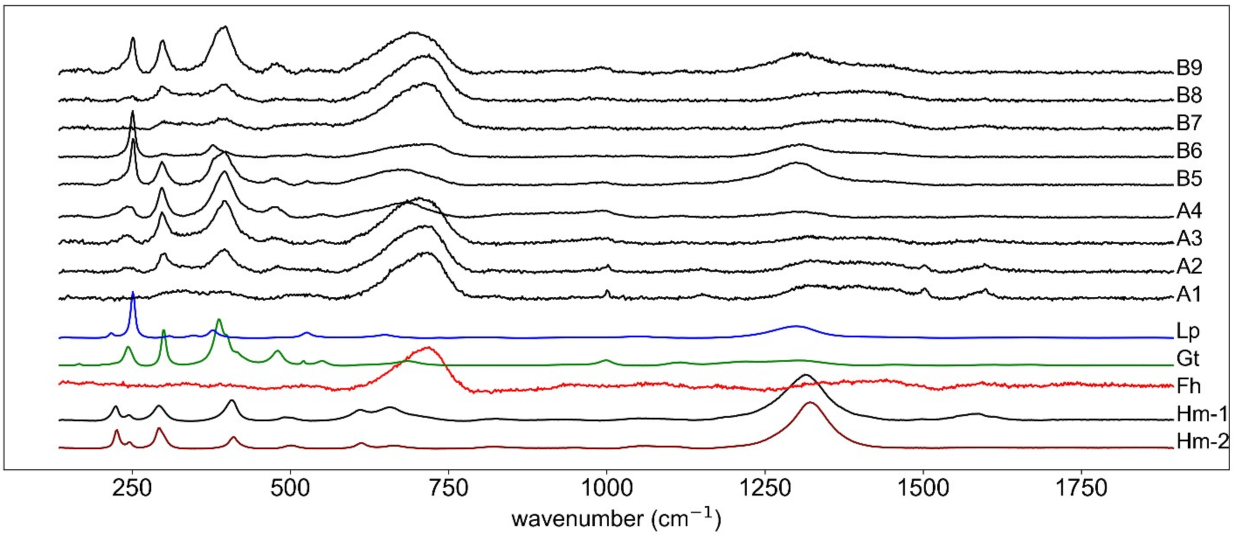

Figure S18: Raman spectra that are displayed in Figure 4G, extended to the full measurement domain (135 to 1900 cm^{-1}) that was used in the component analysis. Spectrum labels refer to the locations marked on the microscope images in Figure 4. Reference spectra for lepidocrocite (Lp), goethite (Gt), ferrihydrite (Fh), beam damage (BD), and hematite (Hm) are measurements using the same instrument and optical set-up. All spectra are normalised to a standard range.

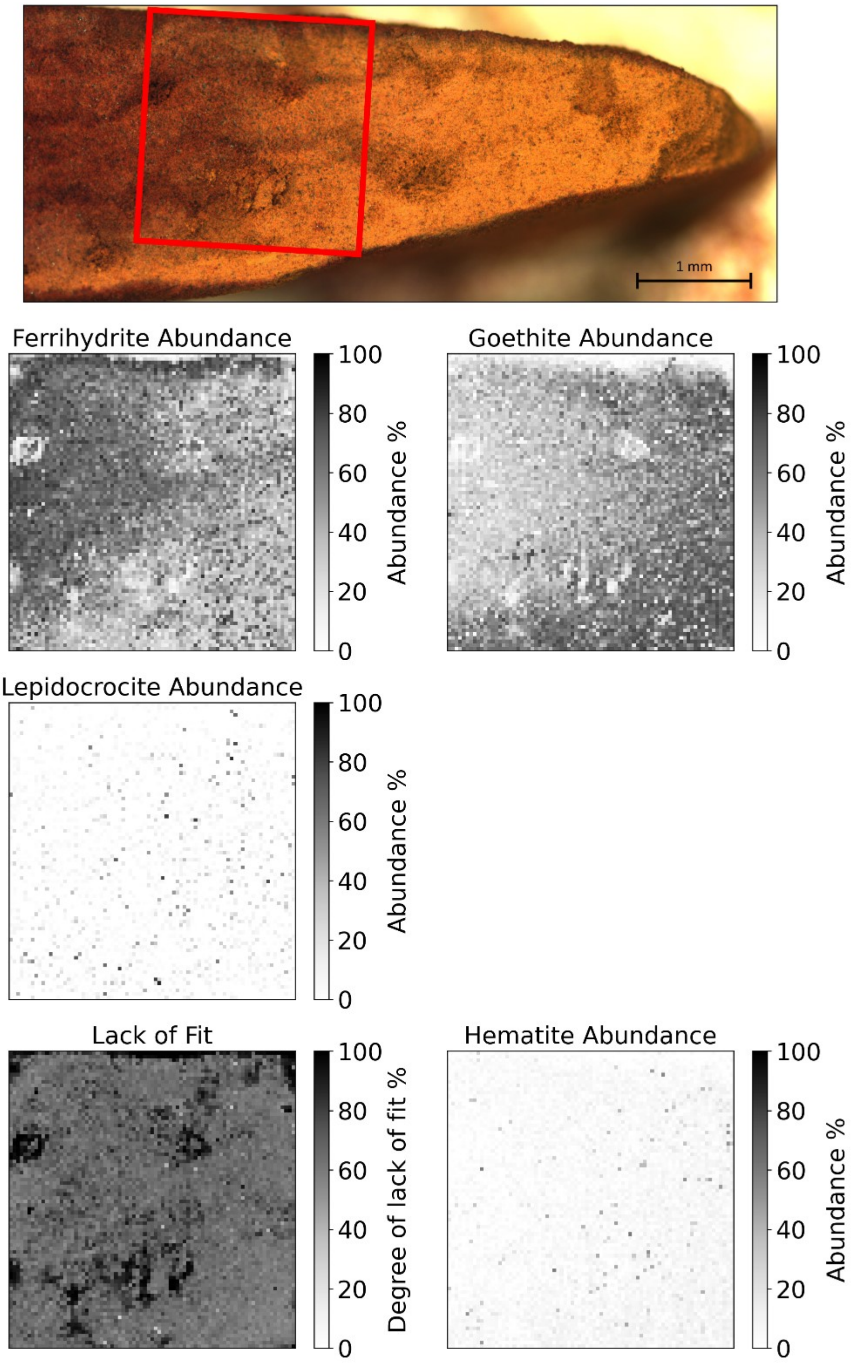

Figure S19: Black and white representation of the Raman map of a mineral aggregate cross section from soil CS as presented in Figure 4.

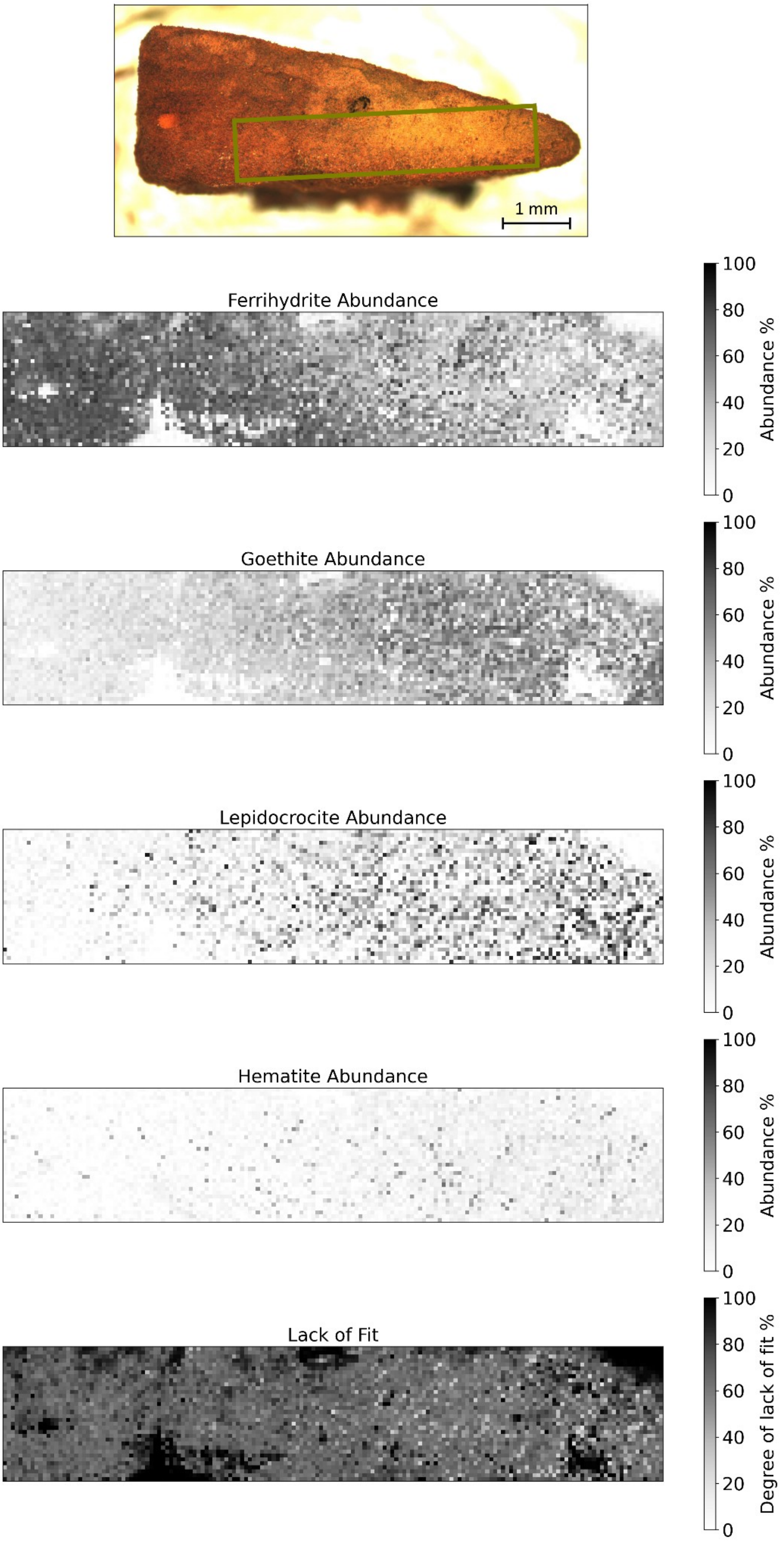

Figure S20: Black and white representation of the Raman map of a mineral aggregate cross-section from soil UB as presented in Figure 4.

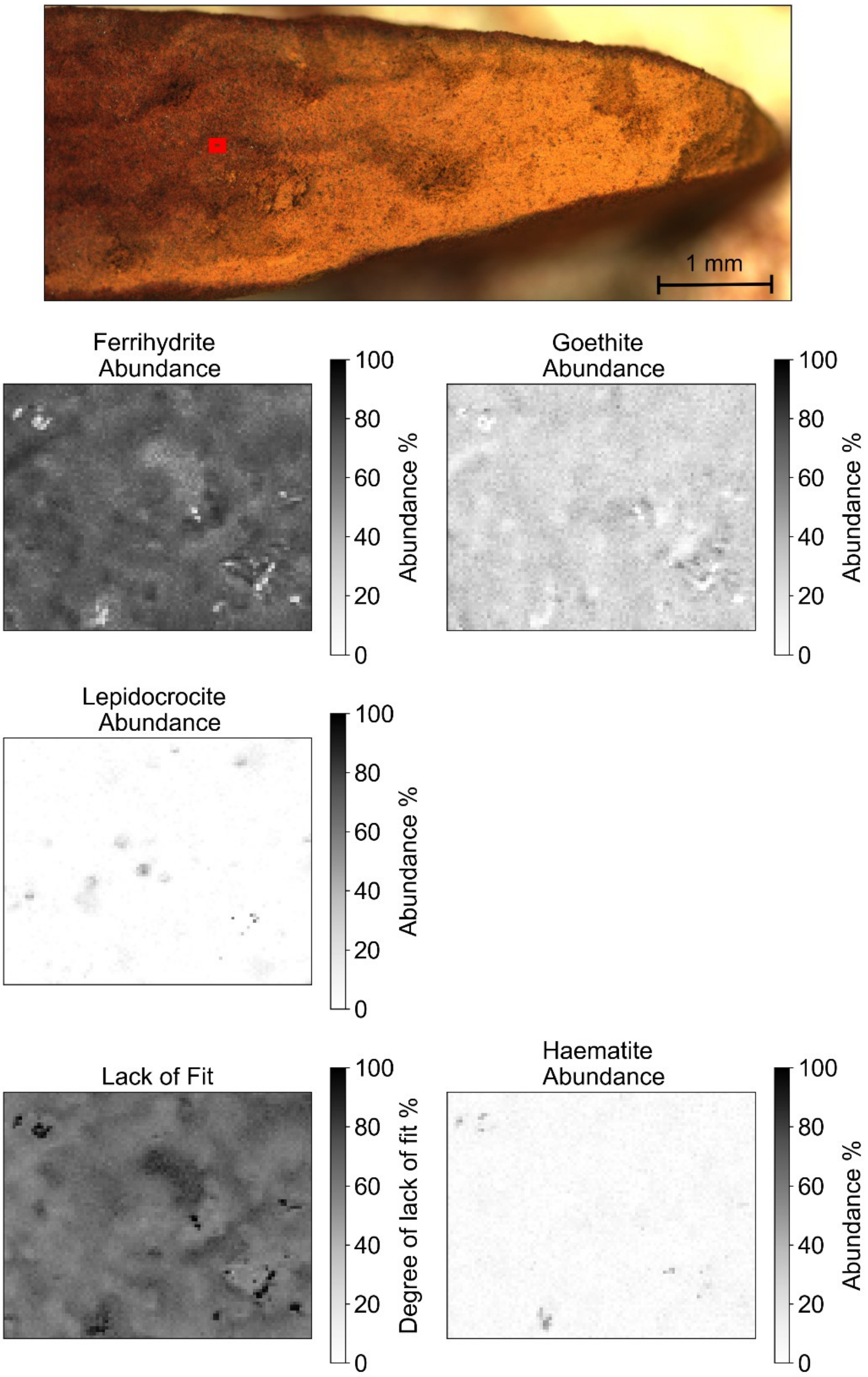

Figure S21: Black and white representation of a 1 µm Raman map of a mineral aggregate from soil CS as presented in Figure 4F.1.

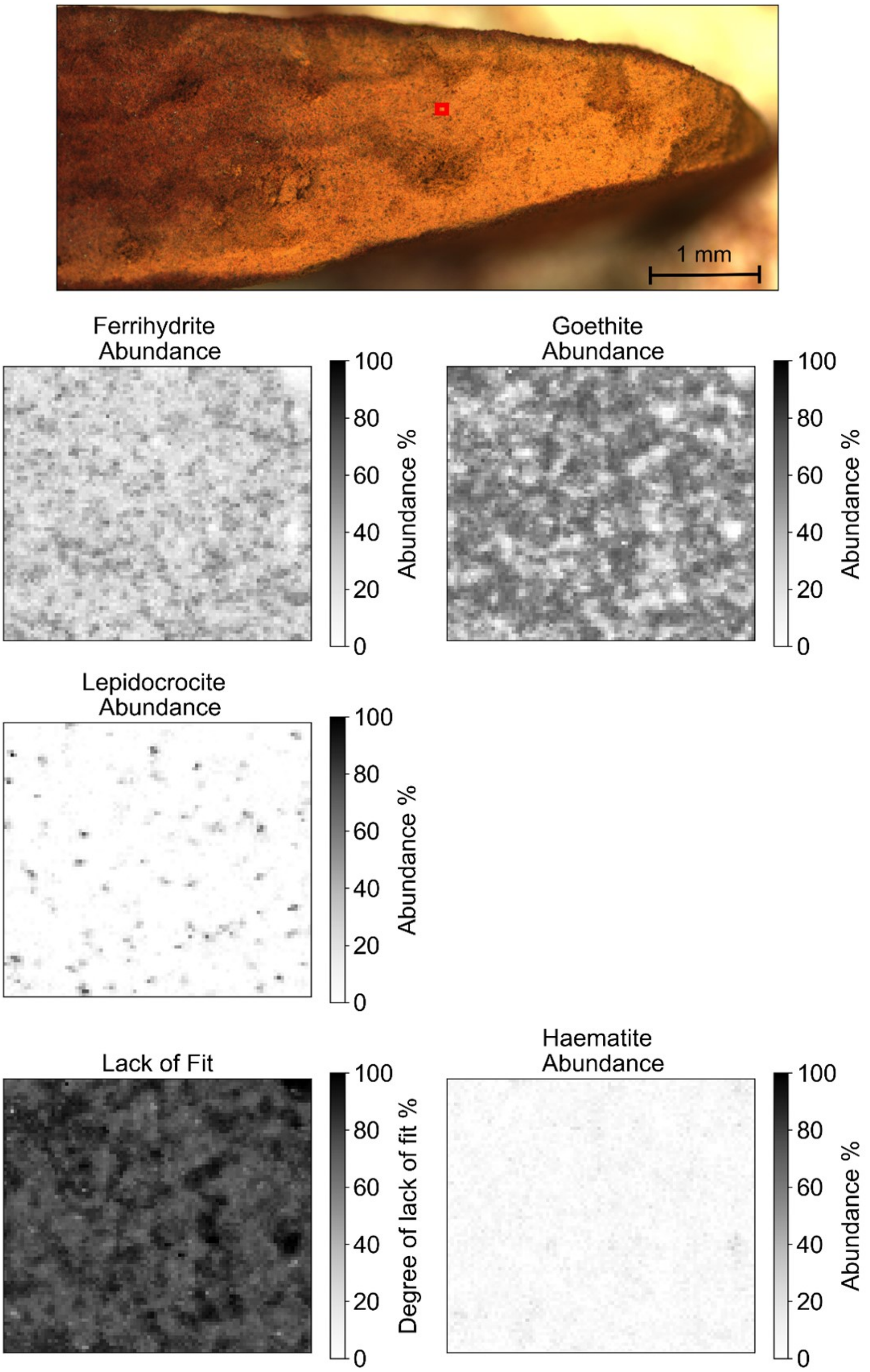

Figure S22: Black and white representation of a 1 µm Raman map of a mineral aggregate cross section from soil CS as presented in Figure 4F.2.

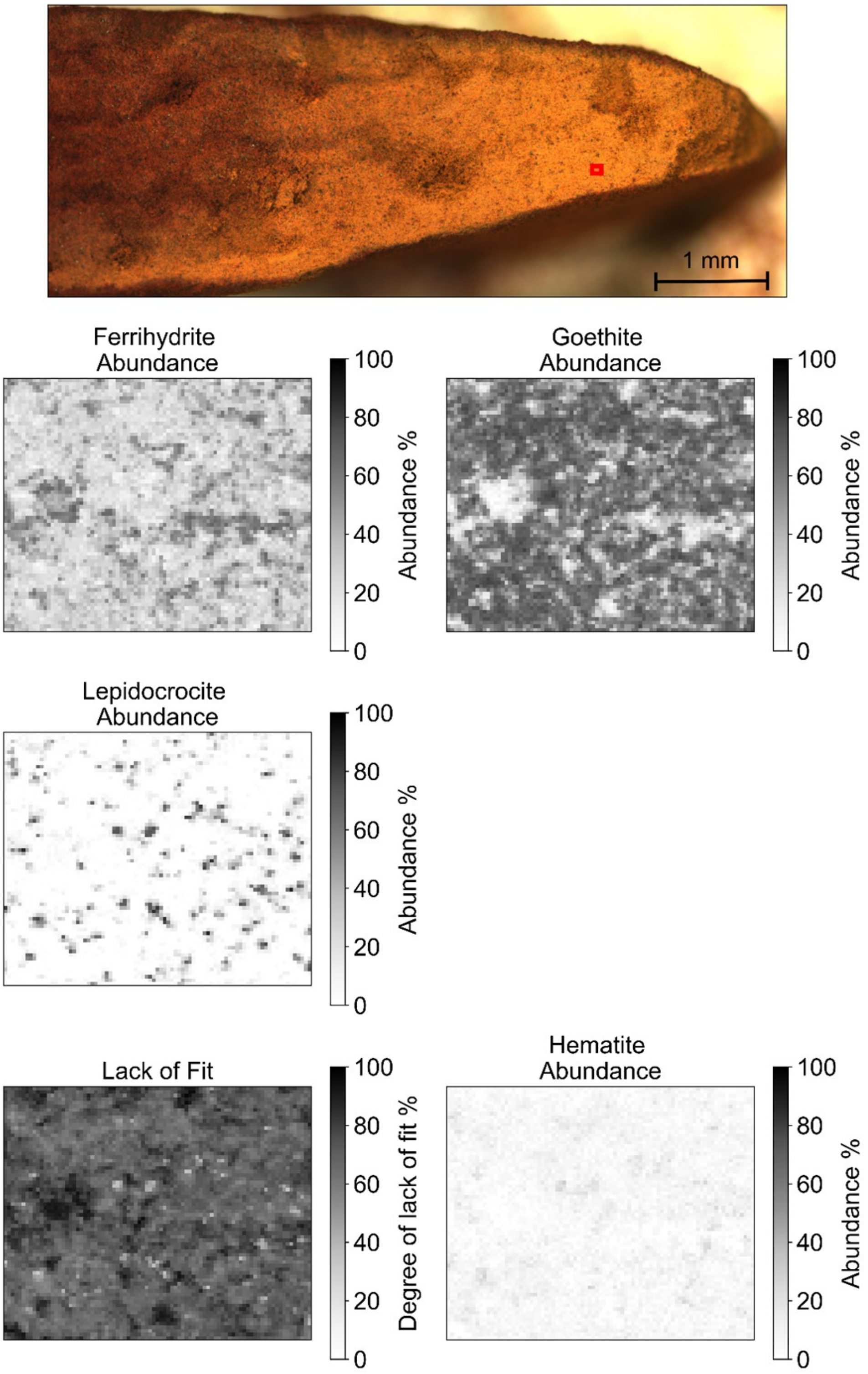

Figure S23: Black and white representation of a 1 µm Raman map of a mineral aggregate cross section from soil CS as presented in Figure 4F.3.

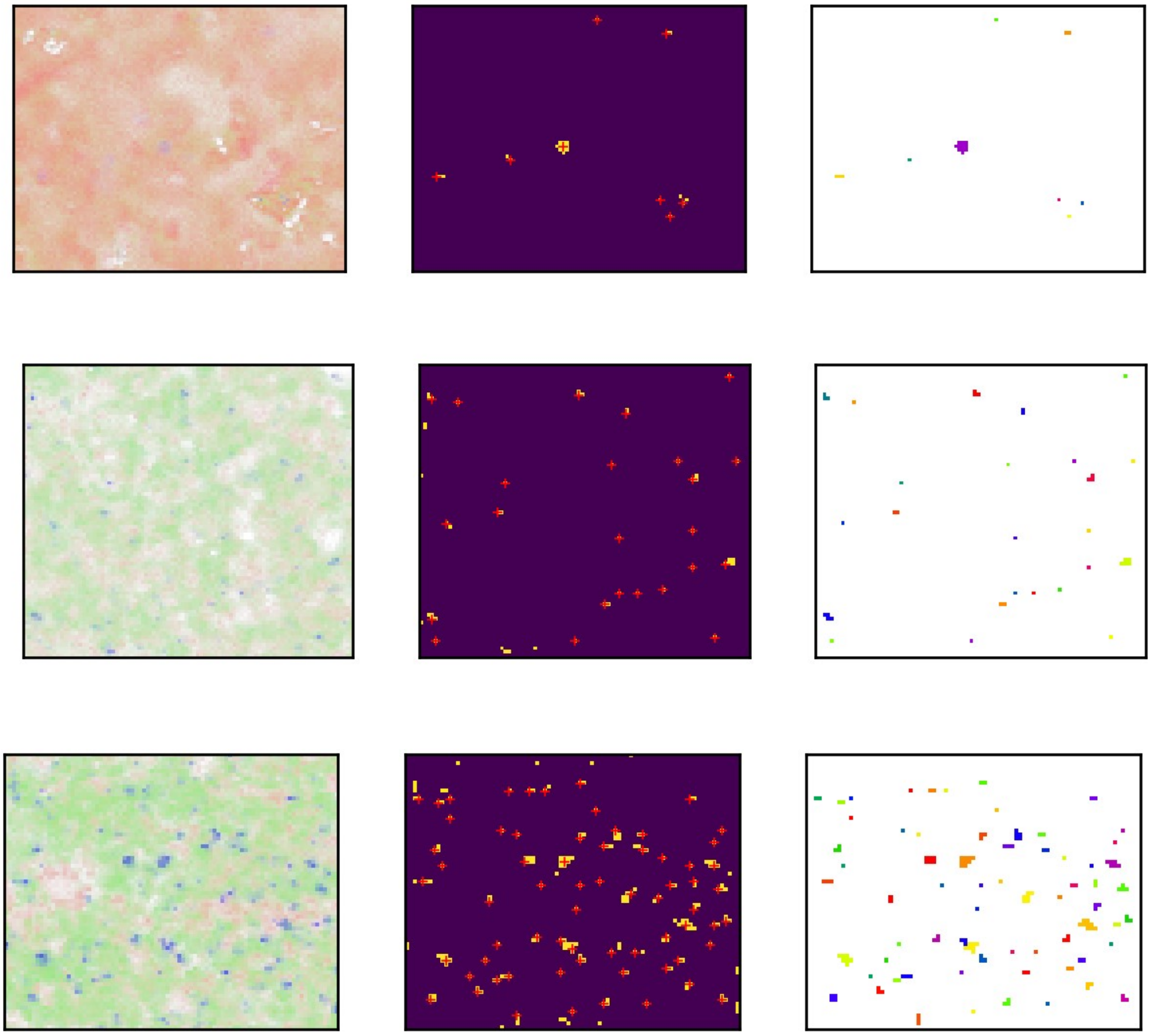

Figure S24: Segmentation of lepidocrocite from 1 µm spectral maps of mineral aggregates from soil CS. Top row corresponds to Figure 4F.1 (all images are 100 x 80 µm), middle row corresponds to Figure 4F.2 (all images are 90 x 80 µm), bottom row corresponds to Figure 4F.3 (all images are 85 x 70 µm). Left: Reproduction of component analysis map (Raman spectral map with component analysis, coloured red for ferrihydrite, green for goethite and blue for lepidocrocite). Middle: Identified lepidocrocite segments (yellow regions) as determined by the watershed model from centres at the red crosses. Particle statistics were measured on these segments. Right: Alternative representation of the lepidocrocite particles with each assigned a different colour for clarity.

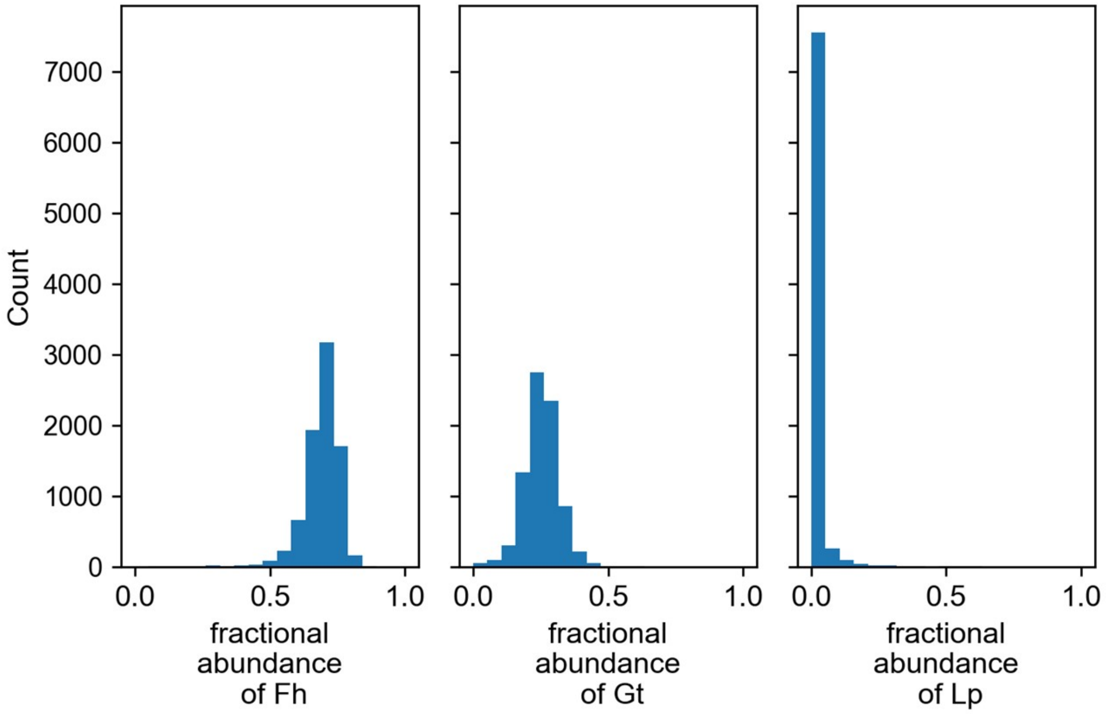

Figure S25: Fractional abundance of ferrihydrite (Fh), goethite (Gt) and lepidocrocite (Lp) in pixels of Figure 4F.1.

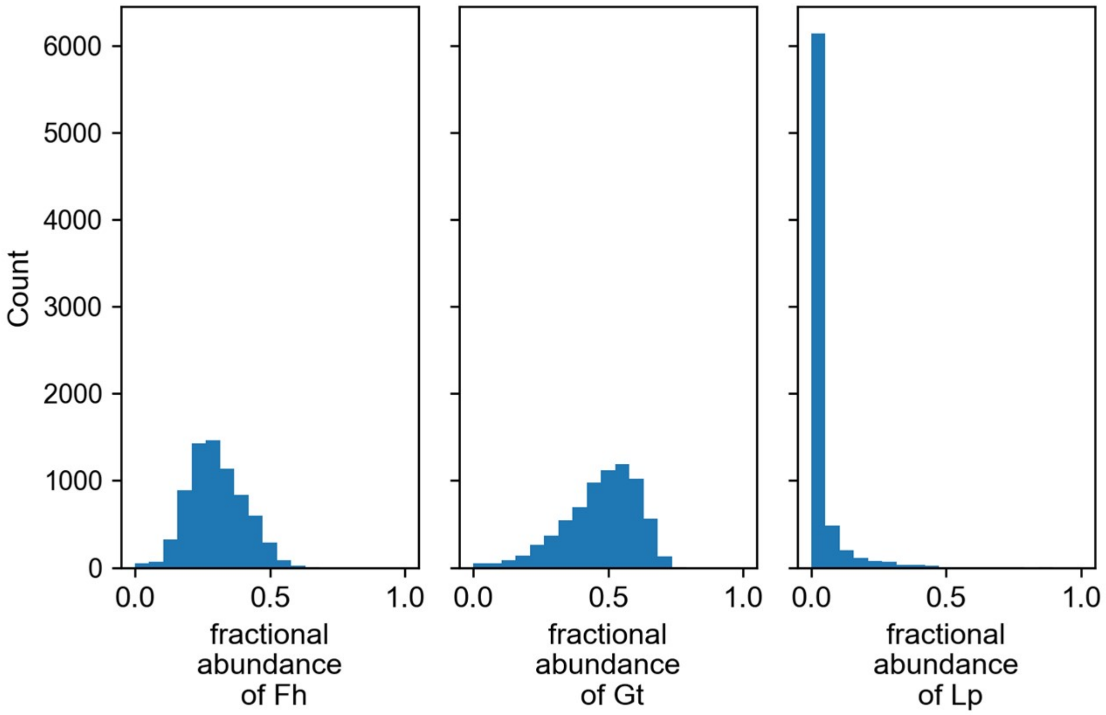

Figure S26: Fractional abundance of ferrihydrite (Fh), goethite (Gt) and lepidocrocite (Lp) in pixels of Figure 4F.2.

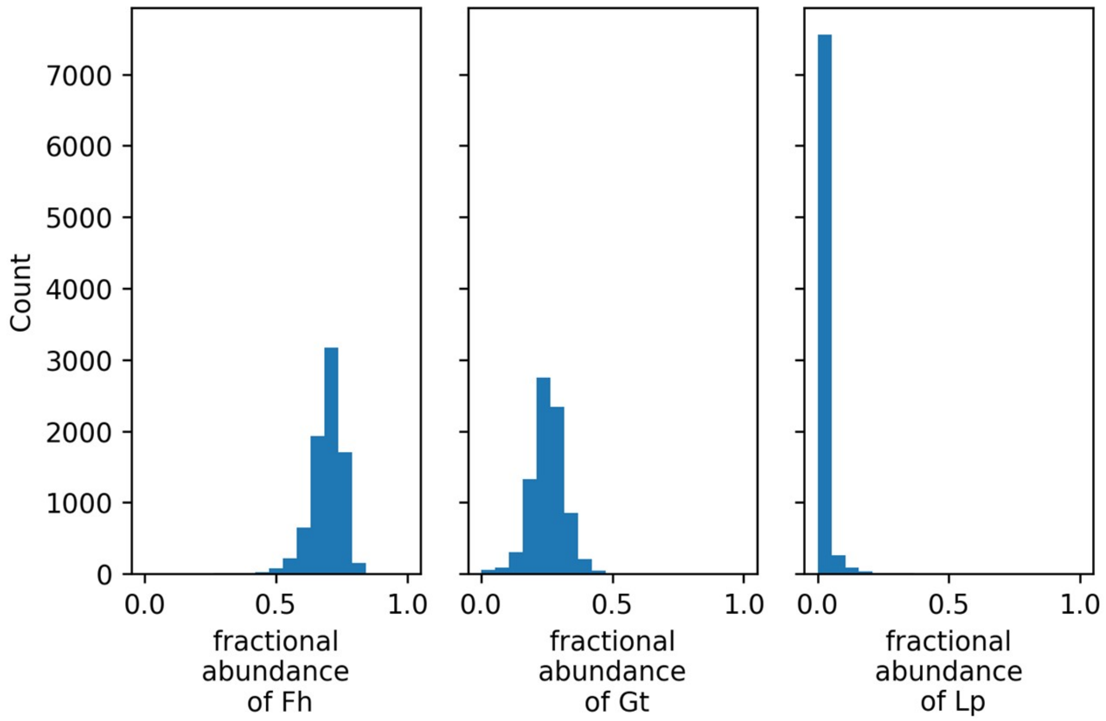

Figure S27: Fractional abundance of ferrihydrite (Fh), goethite (Gt) and lepidocrocite (Lp) in pixels of Figure 4F.3.

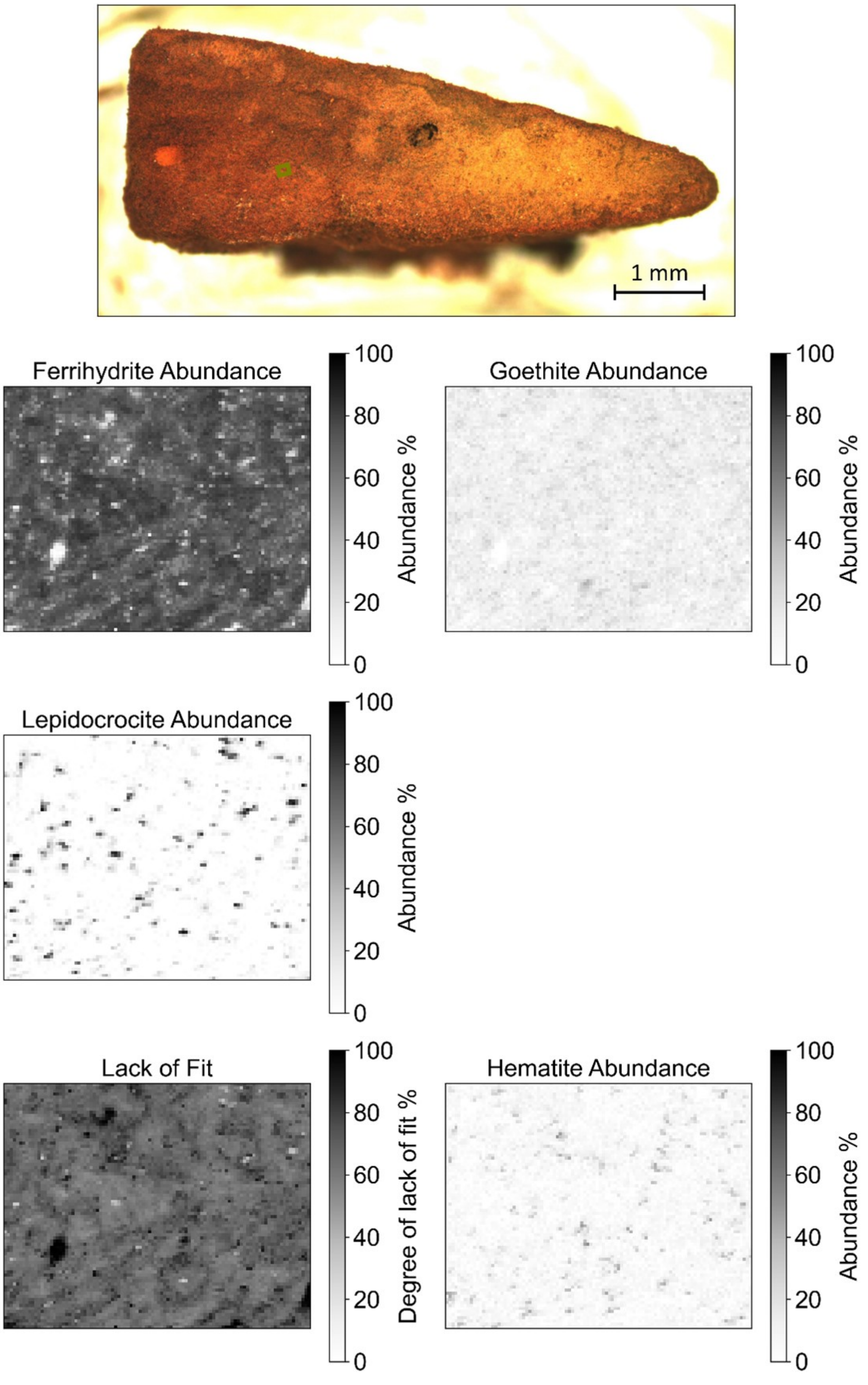

Figure S28: Black and white representation of a 1 µm Raman map of a mineral aggregate cross-section from soil UB as presented in Figure 4F.4.

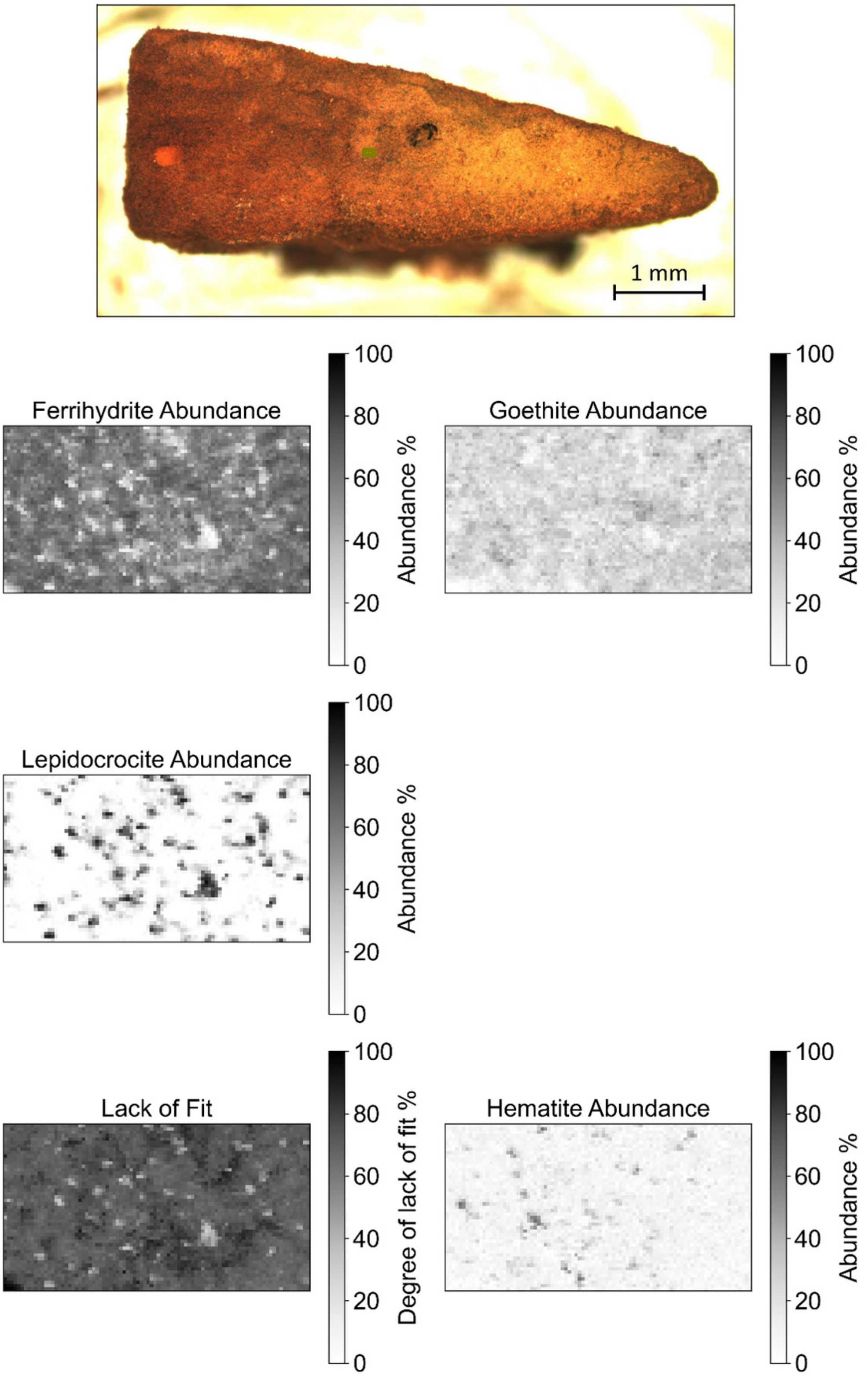

Figure S29: Black and white representation of a 1 µm Raman map of a mineral aggregate cross section from soil UB as presented in Figure 4F.5.

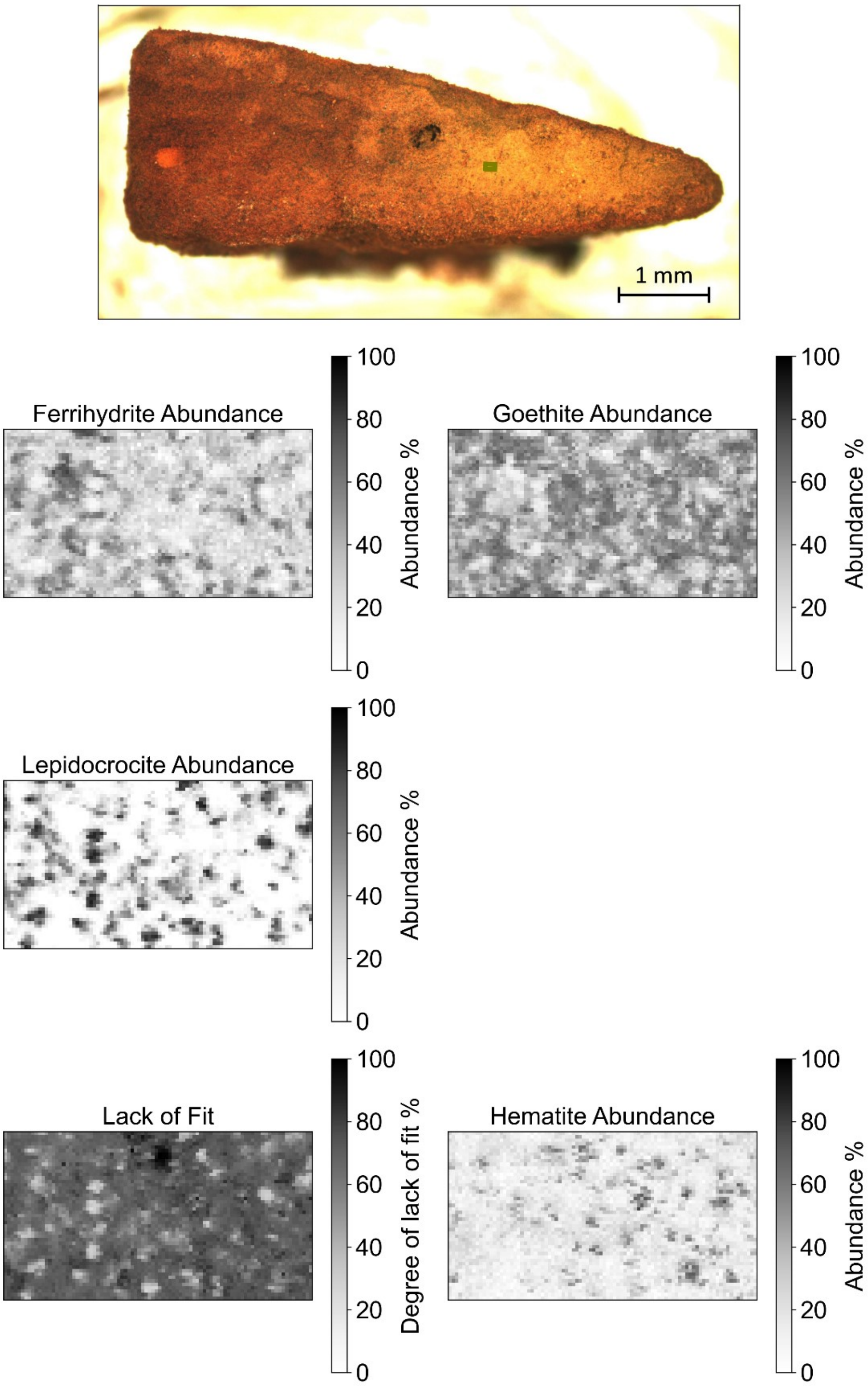

Figure S30: Black and white representation of a 1 µm Raman map of a mineral aggregate cross section from soil UB as presented in Figure 4F.6.

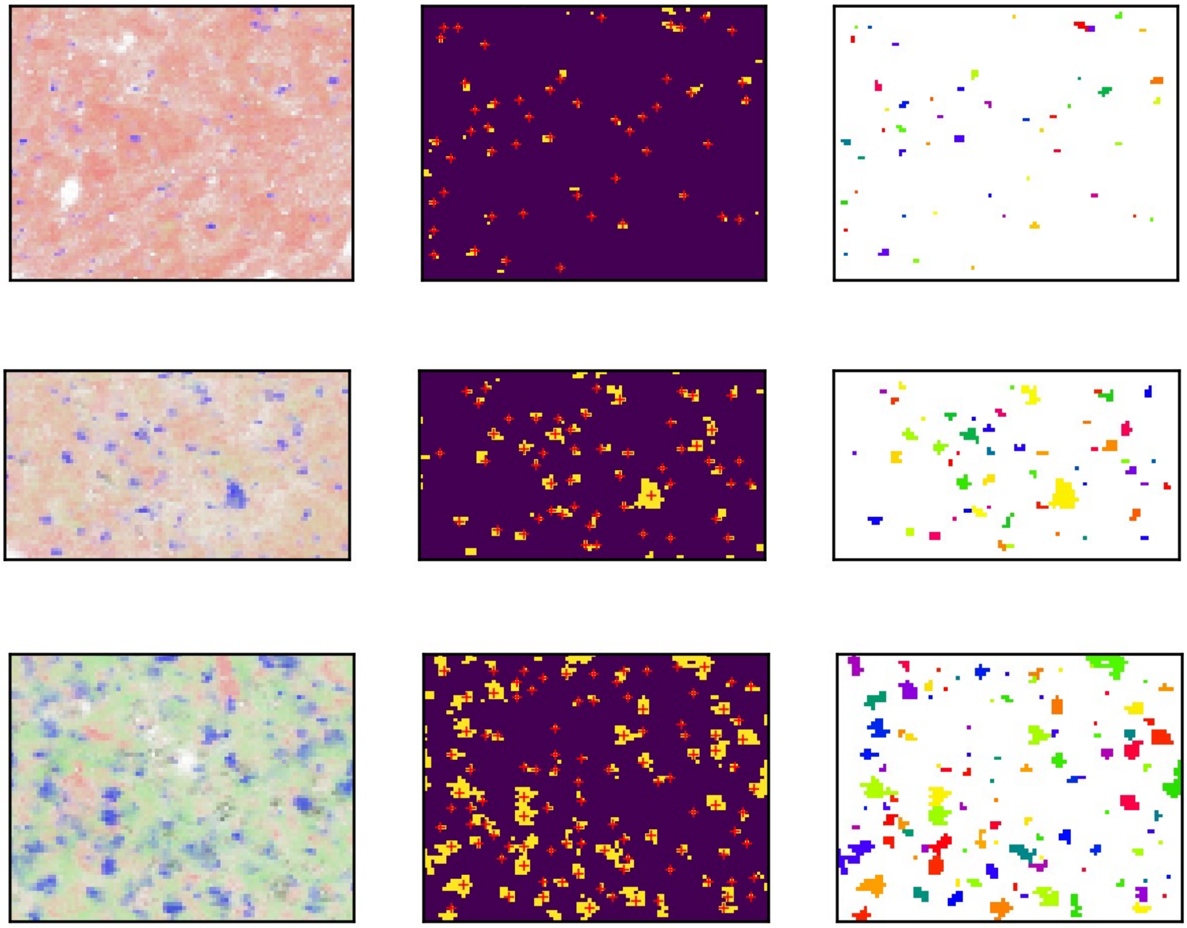

Figure S31: Segmentation of lepidocrocite from spectral maps of soil UB. Top row corresponds to Figure 4F.4 (all images are 100 x 80 µm), middle row corresponds to Figure 4F.5 (all images are 90 x 49 µm), bottom row corresponds to Figure 4F.6 (all images are 90 x 70 µm). Left: Reproduction of component analysis map (Raman spectral map with component analysis, coloured red for ferrihydrite, green for goethite and blue for lepidocrocite). Middle: Identified lepidocrocite segments (yellow regions) as determined by the watershed model from centres at the red crosses. Particle statistics were measured on these segments. Right: Alternative representation of the lepidocrocite particles with each assigned a different colour for clarity.

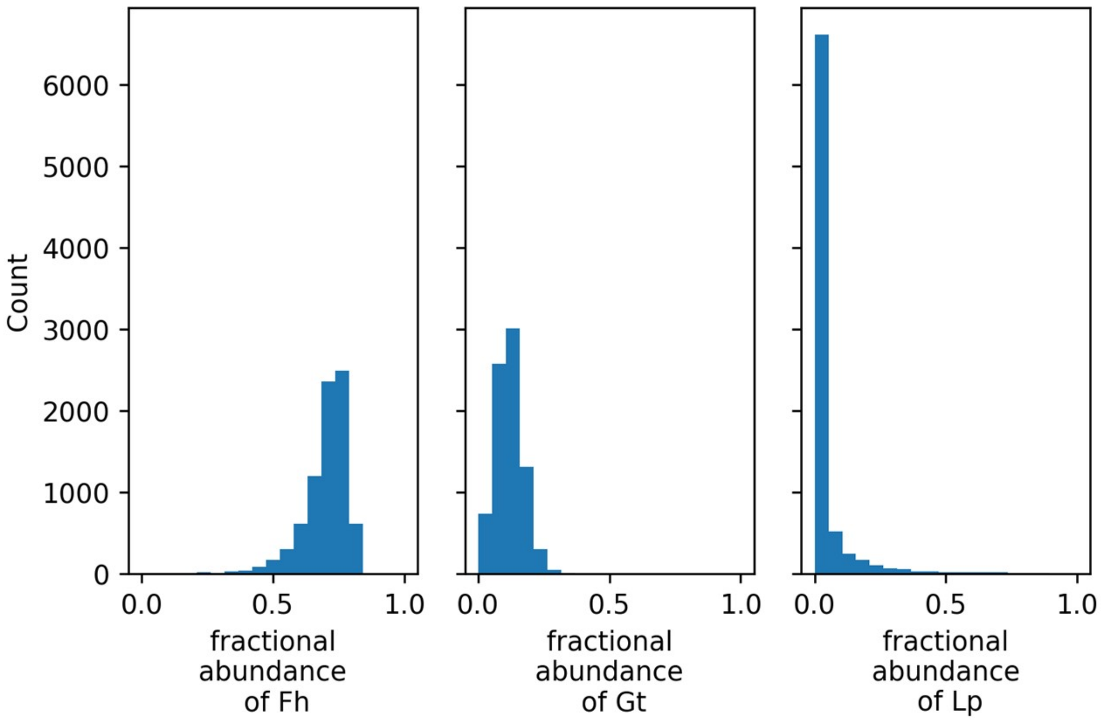

Figure S32: Fractional abundance of ferrihydrite (Fh), goethite (Gt) and lepidocrocite (Lp) in spectral map of UB Figure 4F.4.

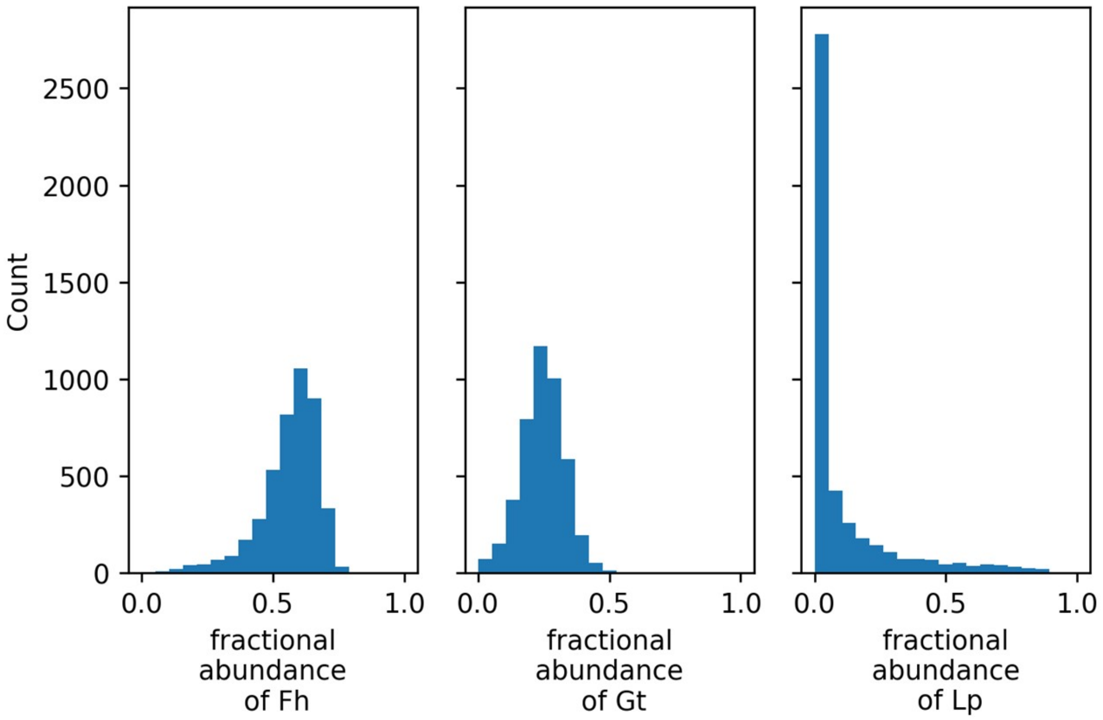

Figure S33: Fractional abundance of ferrihydrite (Fh), goethite (Gt) and lepidocrocite (Lp) in pixels of Figure 4F.5.

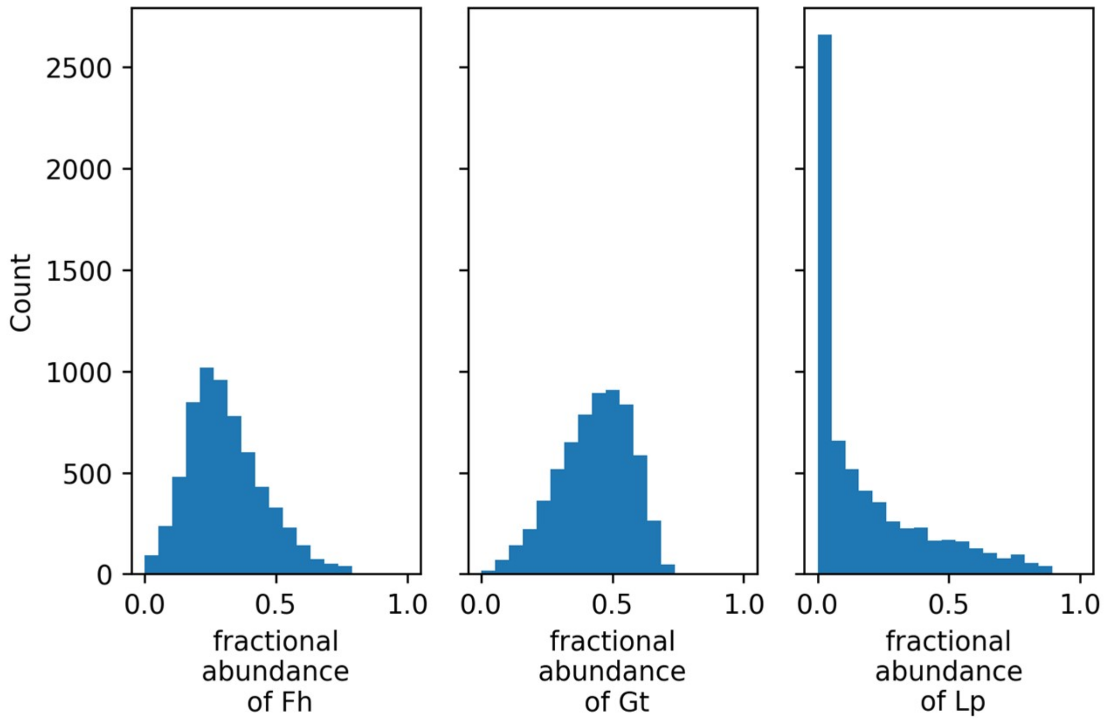

Figure S34: Fractional abundance of ferrihydrite (Fh), goethite (Gt) and lepidocrocite (Lp) in pixels of Figure 4F.6.

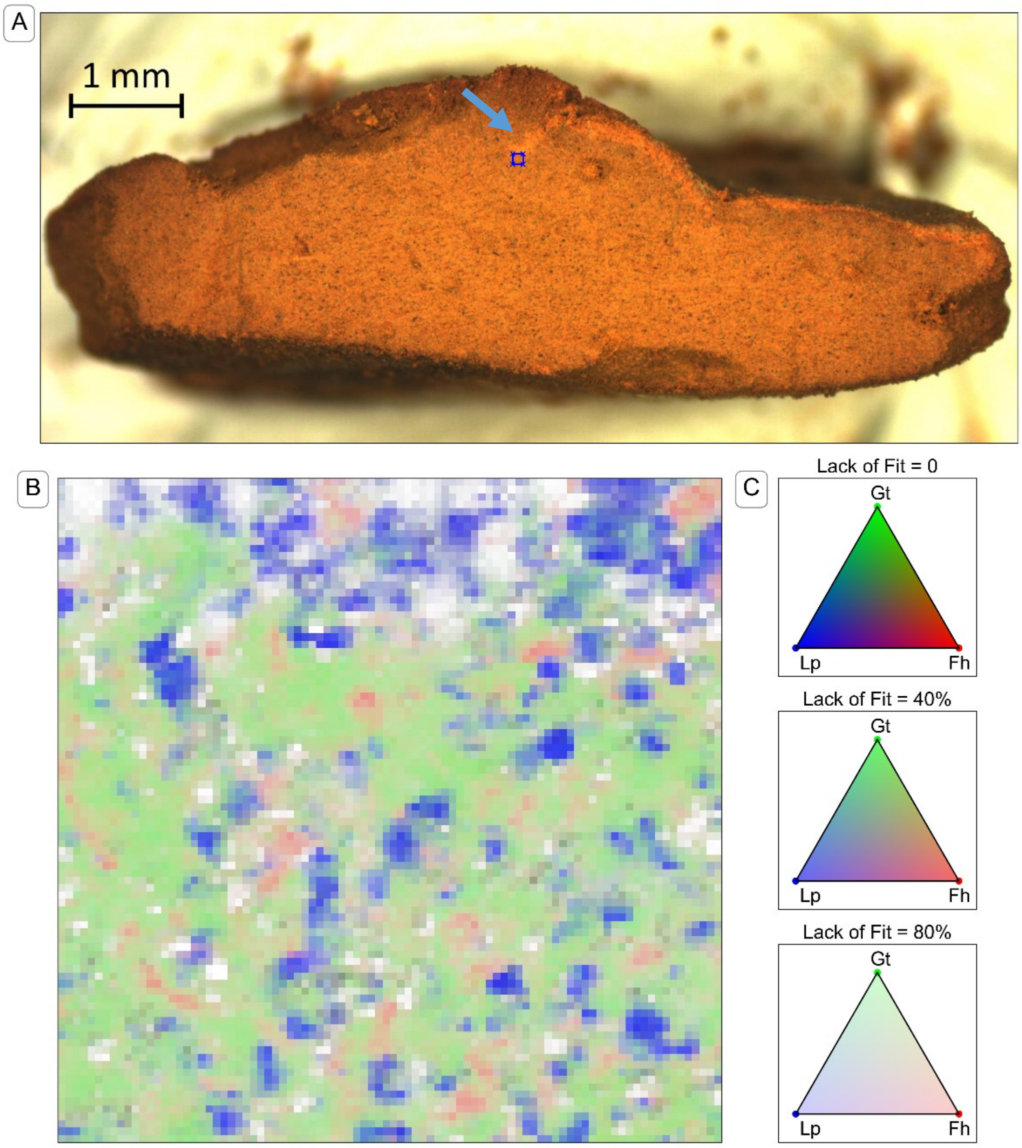

Figure S35: Raman spectroscopy from a sample of a mineral aggregate cross section from soil PT after two weeks of incubation. Panel A: Microscope image with spectral map area overlayed. The arrow points out an intrusion of unidentified Raman-inactive substance that is mentioned in the results section of the main text. Panel B: Spectral map (resolution 1 µm), analysed with component analysis. Colours are based on a Maxwell colour triangle [16] where the ferrihydrite (Fh), goethite (Gt) and lepidocrocite (Lp) from component analysis are plotted as the red, green and blue colour components, respectively. The transparency of the colours is set according to the lack of fit in the component analysis, with bright colours corresponding with complete fits using the three available components, white corresponding to the calculated lack of fit component, and dark colours corresponding to regions where other minerals included in the component analysis but not displayed on the chart (specifically hematite (Hm) from beam damage. Panel C: Legend for colours in spectral map.

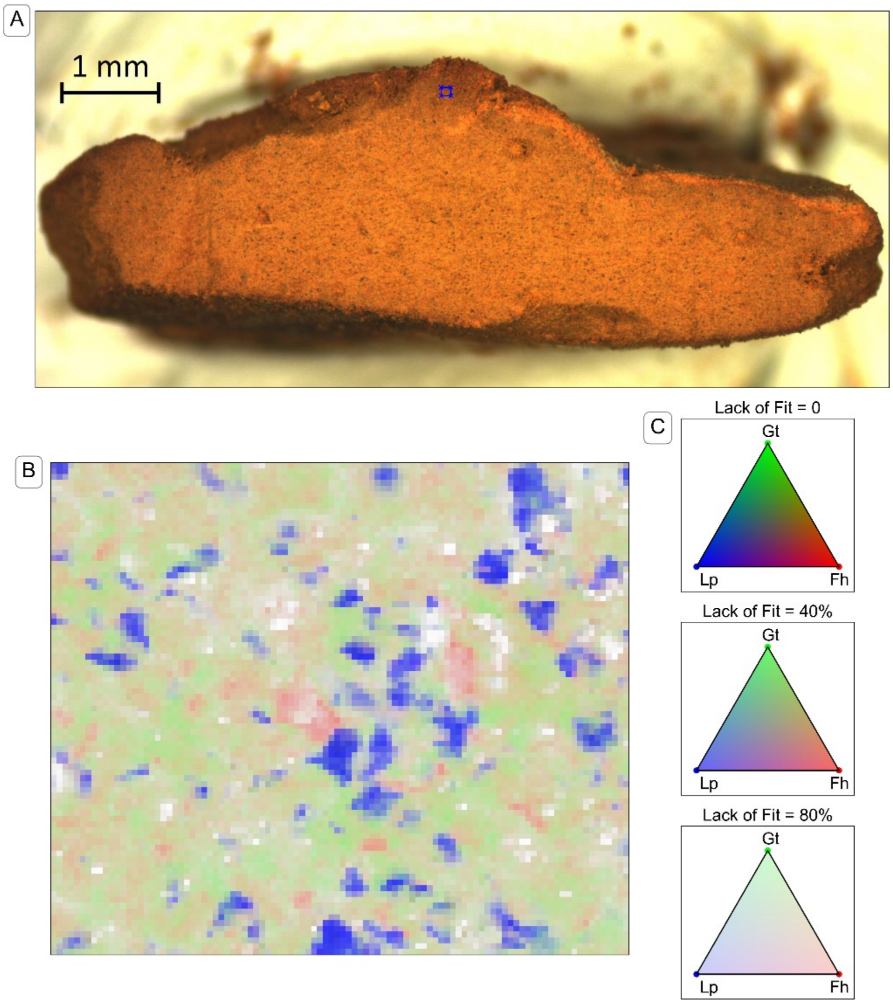

Figure S36: Raman spectroscopy from a sample of a mineral aggregate cross section from soil PT-T after two weeks of incubation. Panel A: Microscope image with spectral map area overlayed. Panel B: Spectral map (resolution 1 µm), analysed with component analysis. Colours are based on a Maxwell colour triangle[16] where the ferrihydrite (Fh), goethite (Gt) and lepidocrocite (Lp) from component analysis are plotted as the red, green and blue colour components, respectively. The transparency of the colours is set according to the lack of fit in the component analysis, with bright colours corresponding with complete fits using the three available components, white corresponding to the calculated lack of fit component, and dark colours corresponding to regions where other minerals included in the component analysis but not displayed on the chart (specifically hematite (Hm) from beam damage. Panel C: Legend for colours in spectral map.

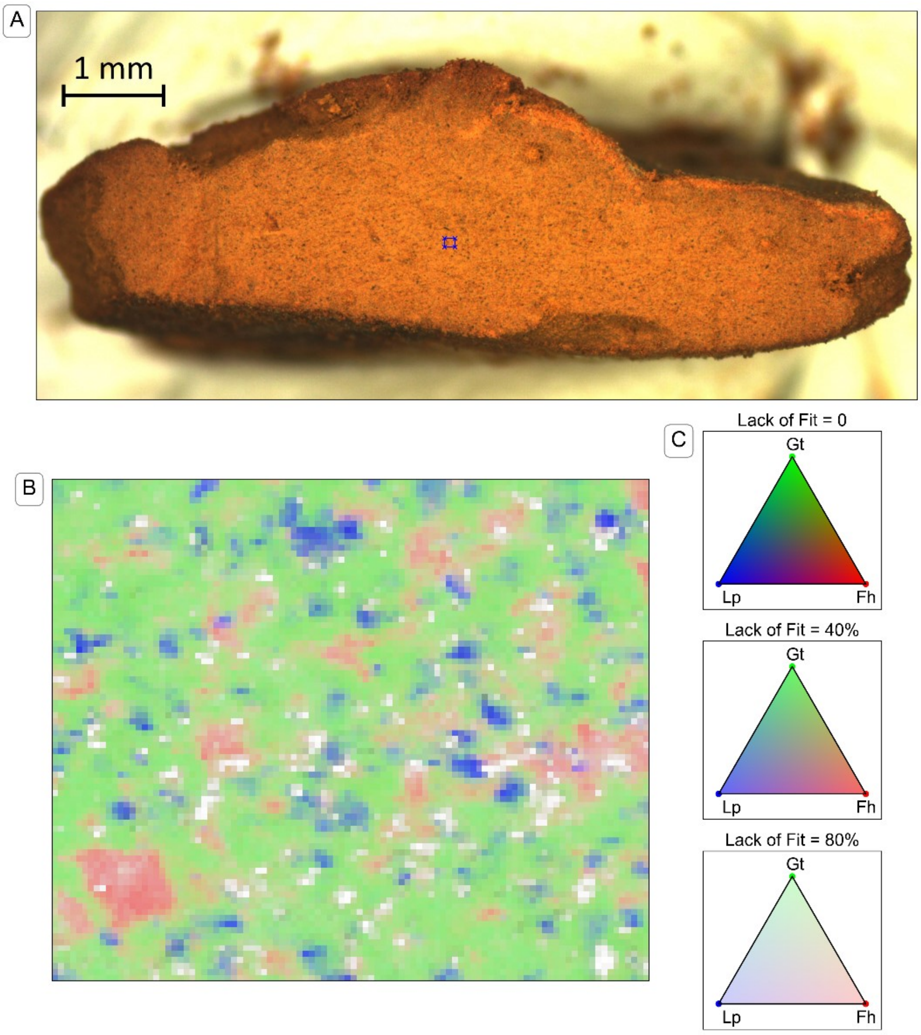

Figure S37: Raman spectroscopy from a sample of a mineral aggregate cross section from soil PT-T after two weeks of incubation. Panel A: Microscope image with spectral map area overlayed. Panel B: Spectral map (resolution 1 µm), analysed with component analysis. Colours are based on a Maxwell colour triangle[16] where the ferrihydrite (Fh), goethite (Gt) and lepidocrocite (Lp) from component analysis are plotted as the red, green and blue colour components, respectively. The transparency of the colours is set according to the lack of fit in the component analysis, with bright colours corresponding with complete fits using the three available components, white corresponding to the calculated lack of fit component, and dark colours corresponding to regions where other minerals included in the component analysis but not displayed on the chart (specifically hematite (Hm) from beam damage. Panel C: Legend for colours in spectral map.

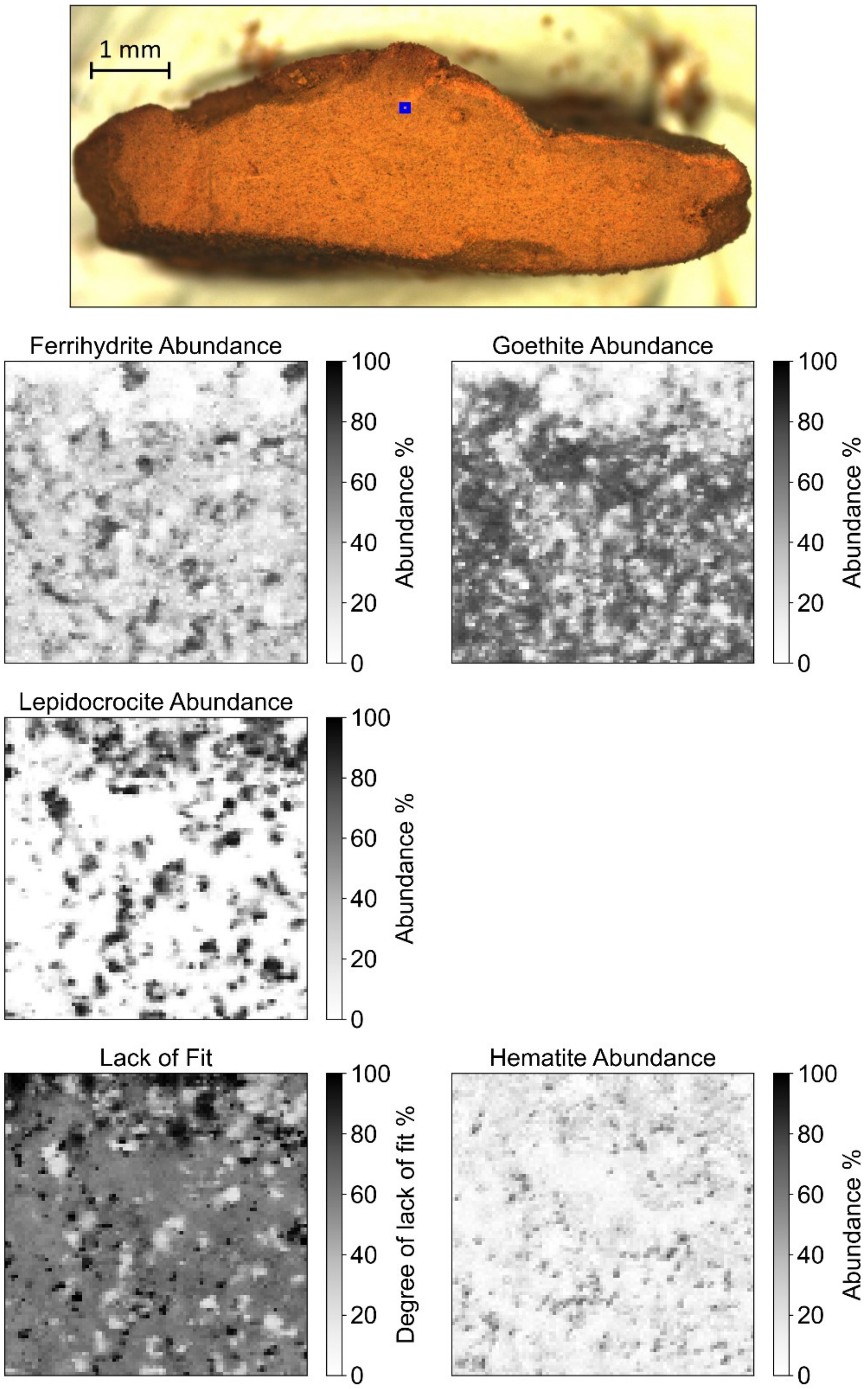

Figure S38 Black and white representation of a 1 µm Raman map of a mineral aggregate cross section from soil PT-T as presented in Figure S35.

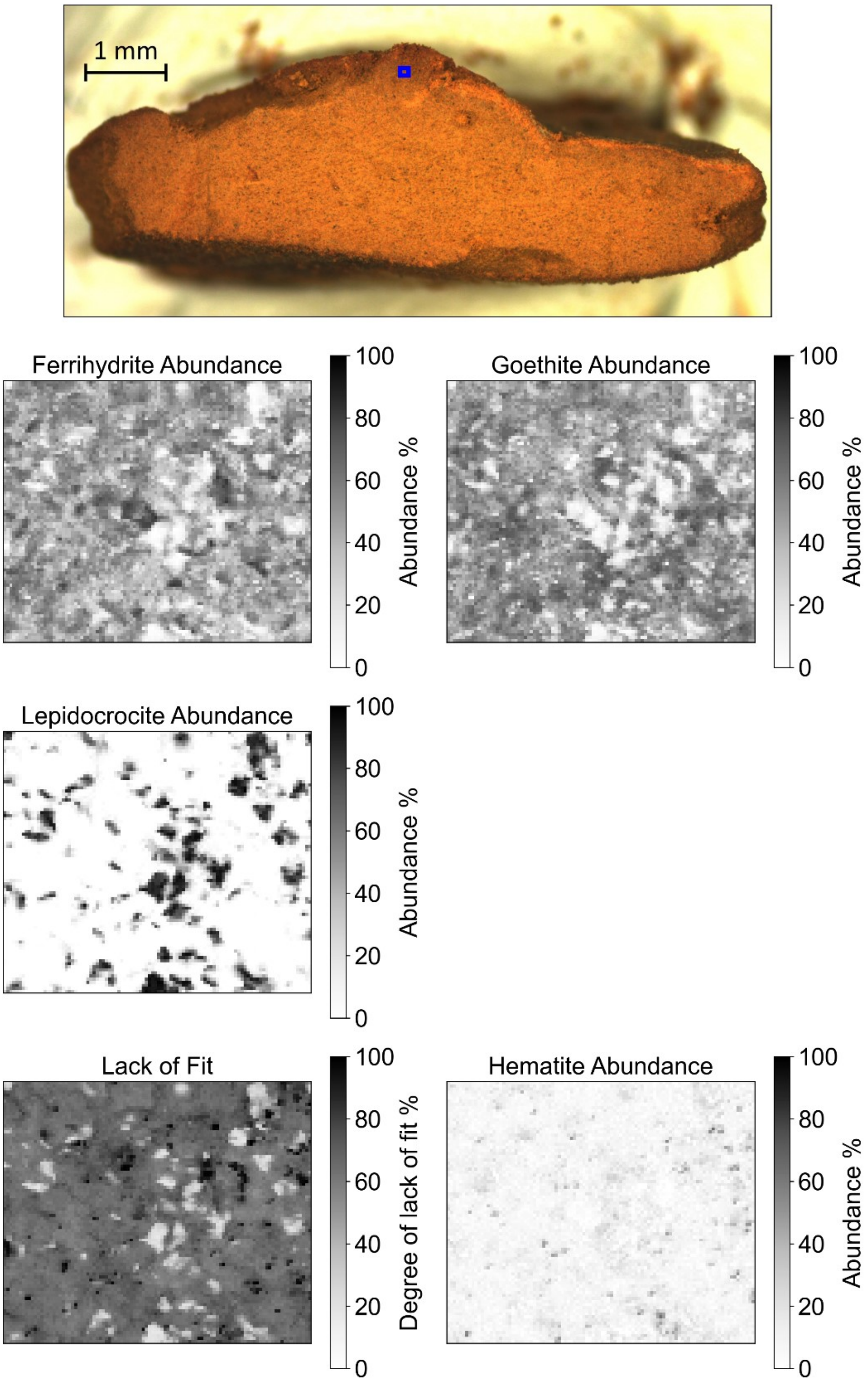

Figure S39: Black and white representation of a 1 µm Raman map of a mineral aggregate cross section from soil PT-T as presented in Figure S36.

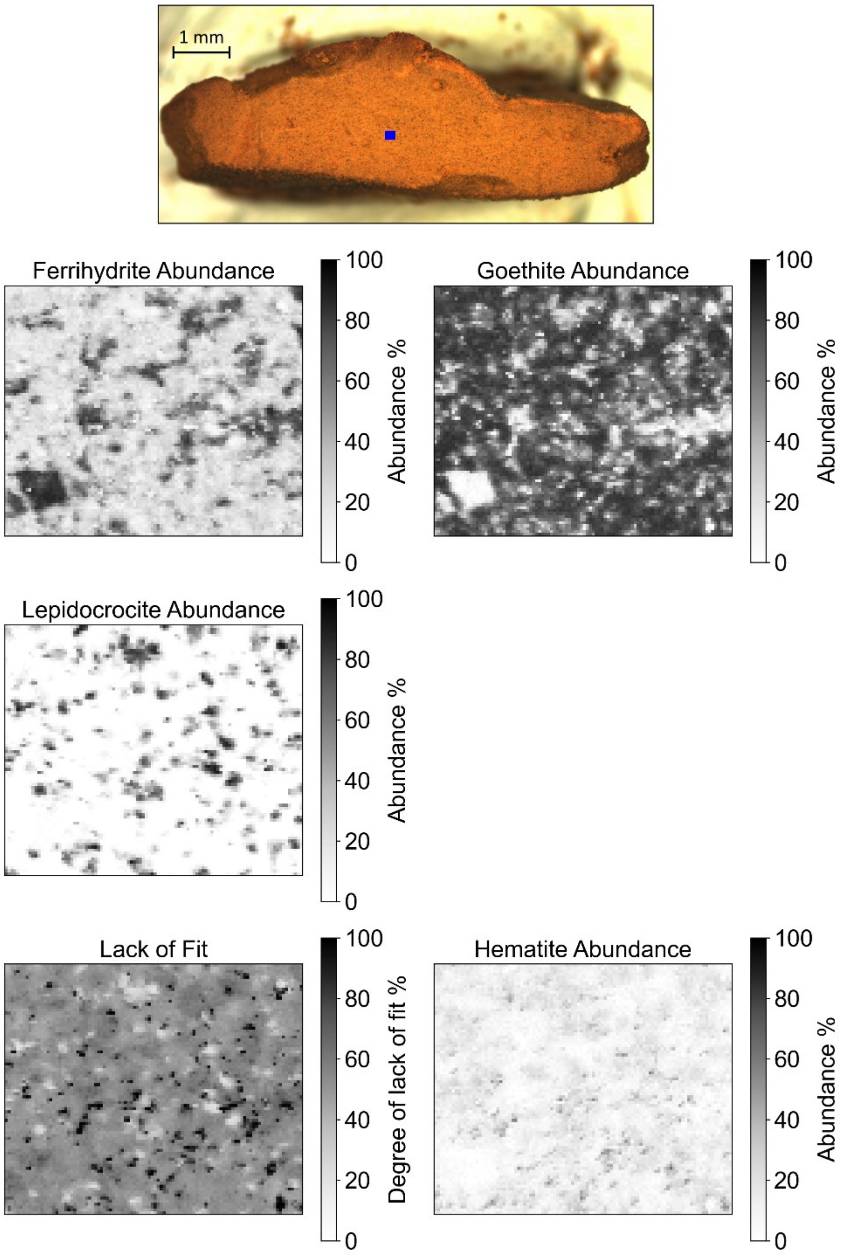

Figure S40: Black and white representation of a 1 µm Raman map of a mineral aggregate cross section from soil PT-T as presented in Figure S37.

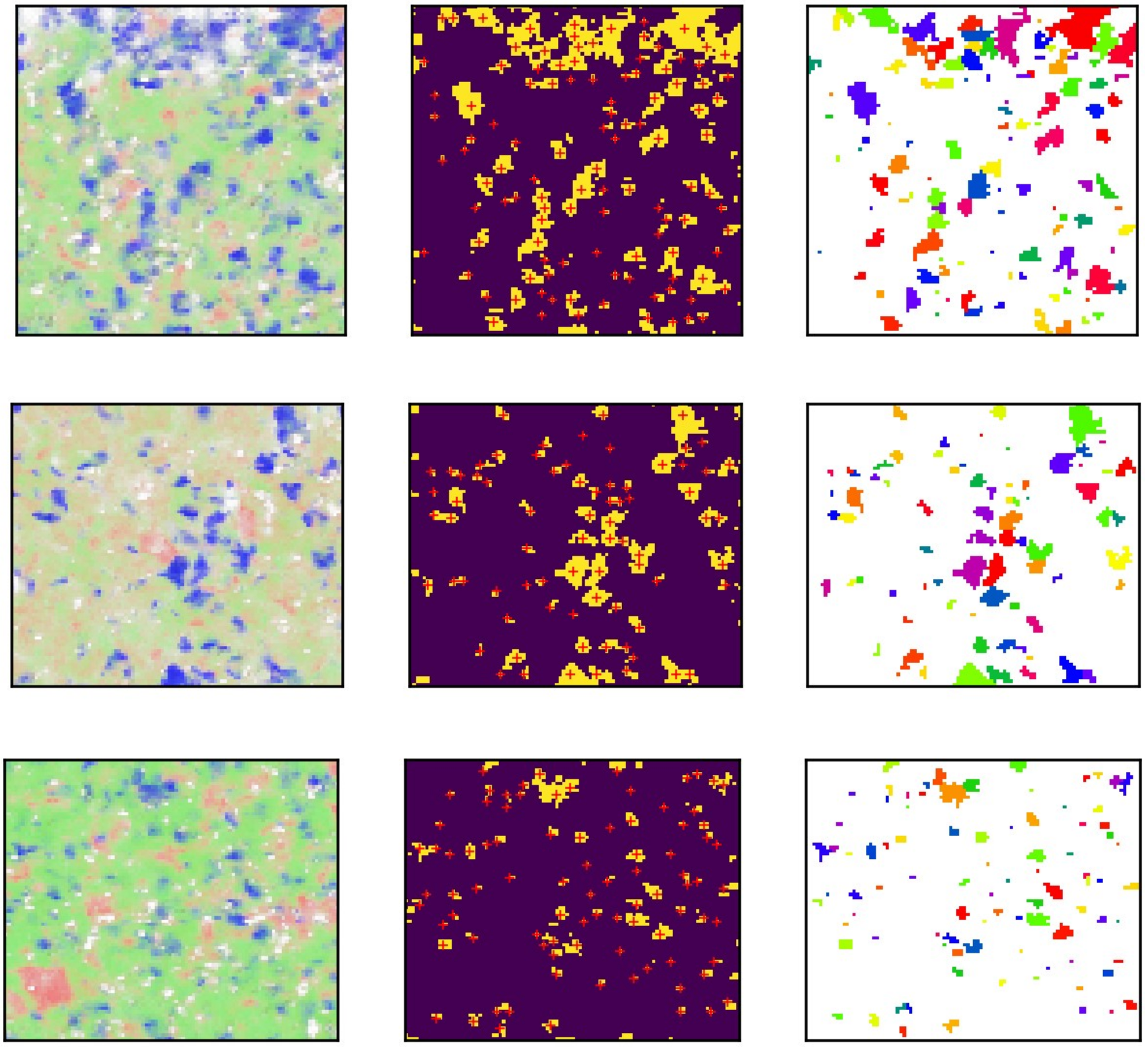

Figure S41: Segmentation of Lp from spectral maps of mineral aggregate cross sections from soil PT-T. Top row corresponds to Figure S35 (all images are 90 x 90 µm), middle row corresponds to Figure S36 (all images are 100 x 85 µm), bottom row corresponds to Figure S37 (all images are 100 x 84 µm). Left: Reproduction of component analysis map (Raman spectral map with component analysis, coloured red for ferrihydrite, green for goethite and blue for lepidocrocite). Middle: Identified lepidocrocite segments (yellow regions) as determined by the watershed model from centres at the red crosses. Particle statistics were measured on these segments Right: Alternative representation of the lepidocrocite particles with each assigned a different colour for clarity.

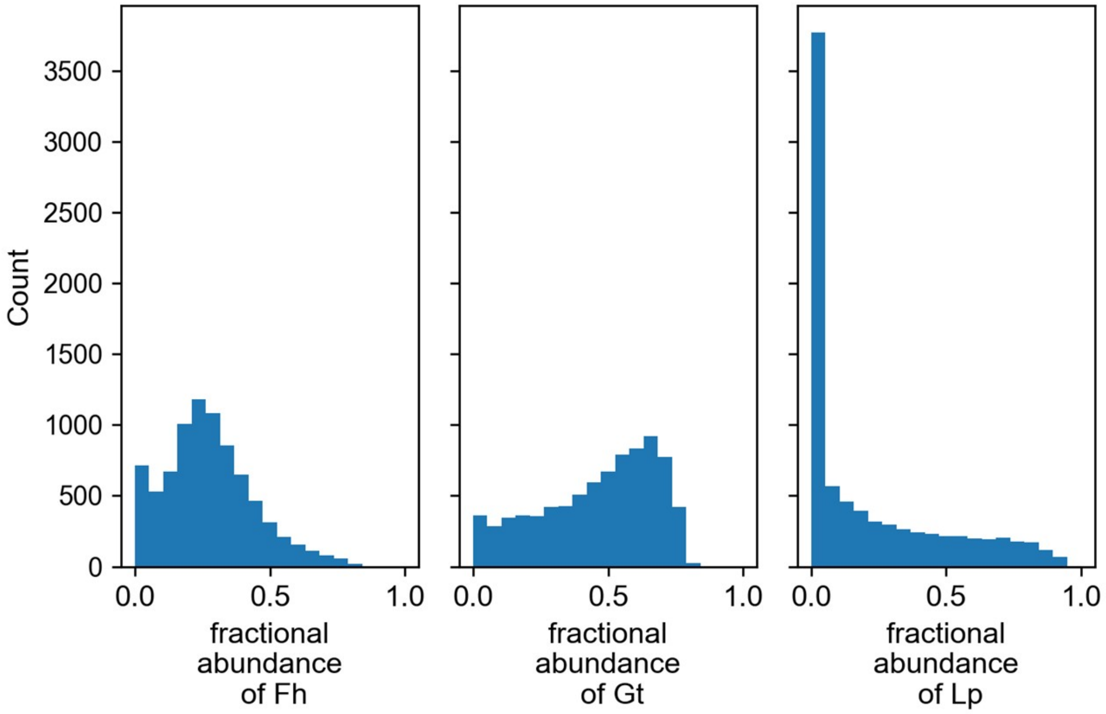

Figure S42: Fractional abundance of ferrihydrite (Fh), goethite (Gt) and lepidocrocite (Lp) in pixels of Figure S35.

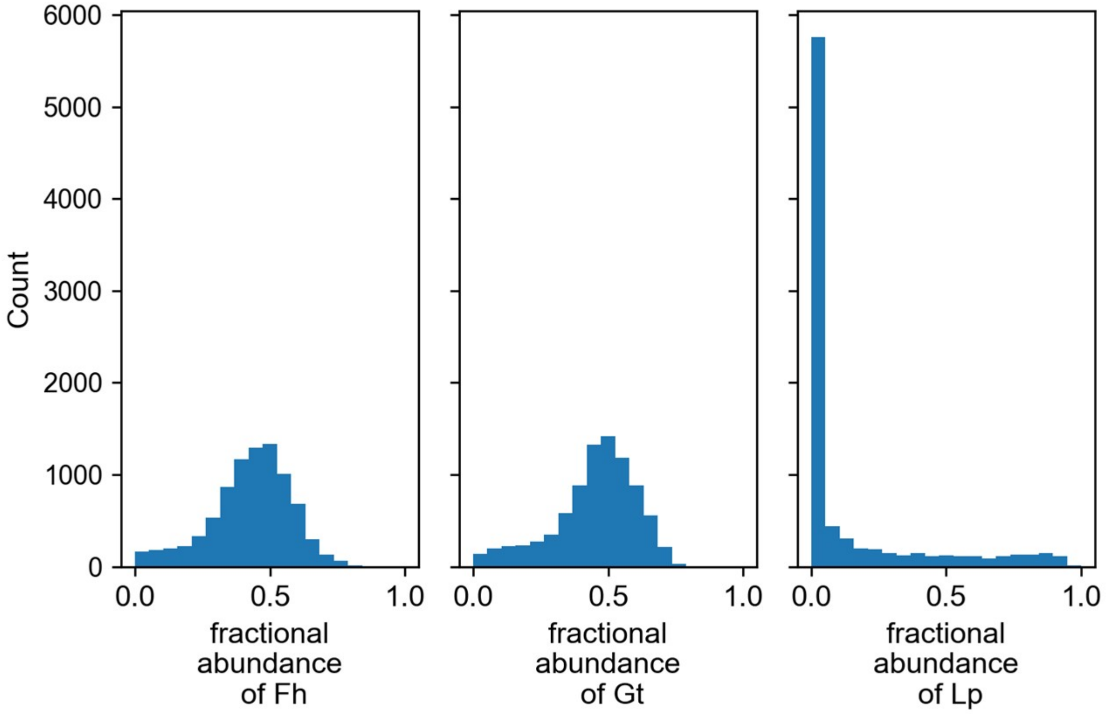

Figure S43: Fractional abundance of ferrihydrite (Fh), goethite (Gt) and lepidocrocite (Lp) in pixels of Figure S36.

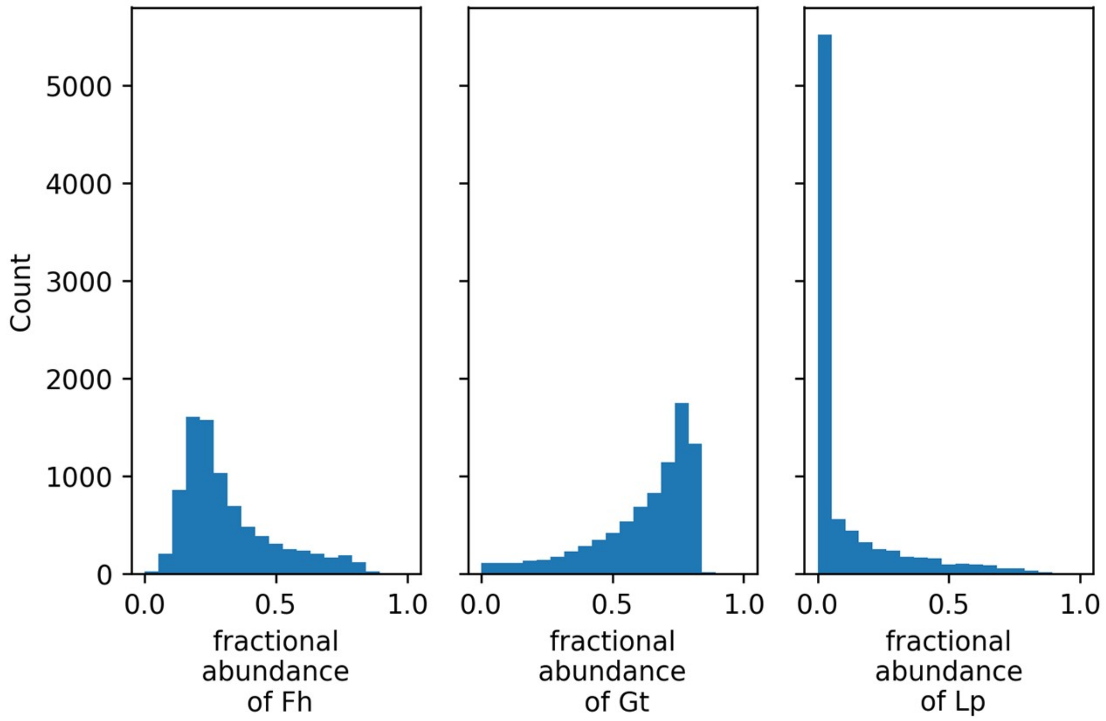

Figure S44: Fractional abundance of ferrihydrite (Fh), goethite (Gt) and lepidocrocite (Lp) in pixels of Figure S37.

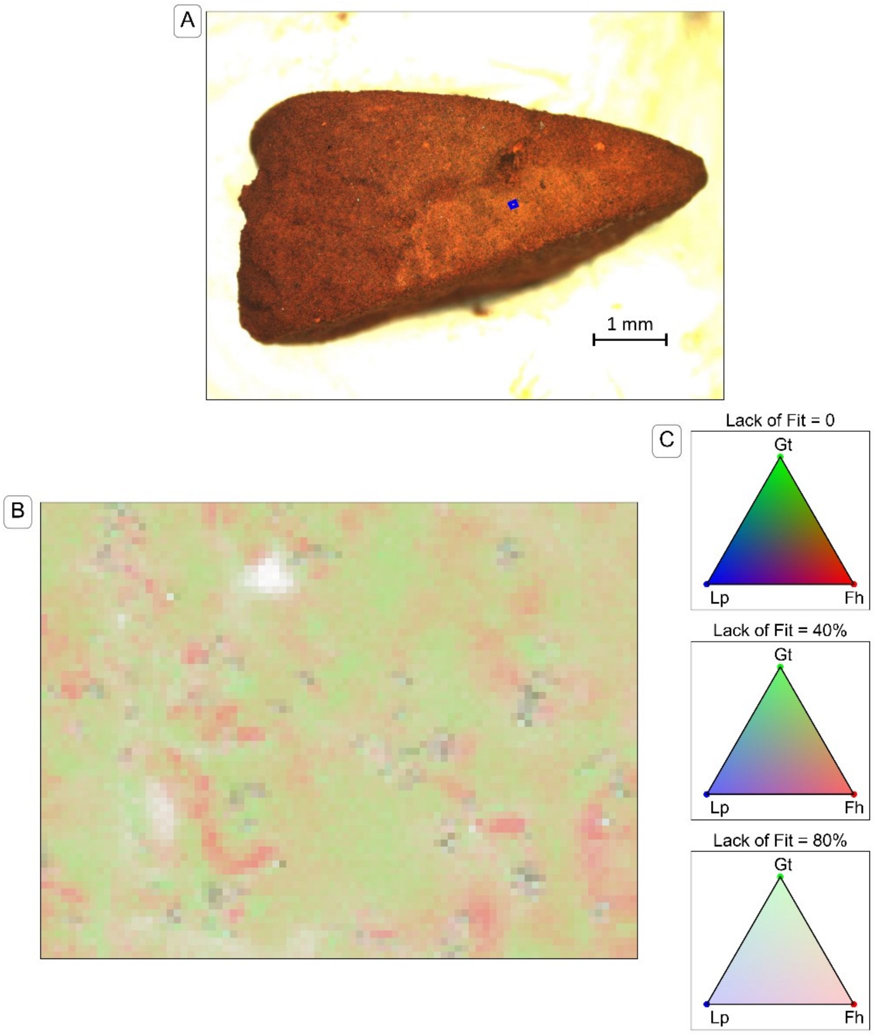

Figure S45: Raman spectroscopy from a sample of a mineral aggregate from soil BD after two weeks of incubation. Panel A: Microscope image with spectral map area overlayed. Panel B: Spectral map (resolution 1 µm), analysed with component analysis. Colours are based on a Maxwell colour triangle [16] where the ferrihydrite (Fh), goethite (Gt) and lepidocrocite (Lp) from component analysis are plotted as the red, green and blue colour components, respectively. The transparency of the colours is set according to the lack of fit in the component analysis, with bright colours corresponding with complete fits using the three available components, white corresponding to the calculated lack of fit component, and dark colours corresponding to regions where other minerals included in the component analysis but not displayed on the chart (specifically hematite (Hm) from beam damage. Panel C: Legend for colour.

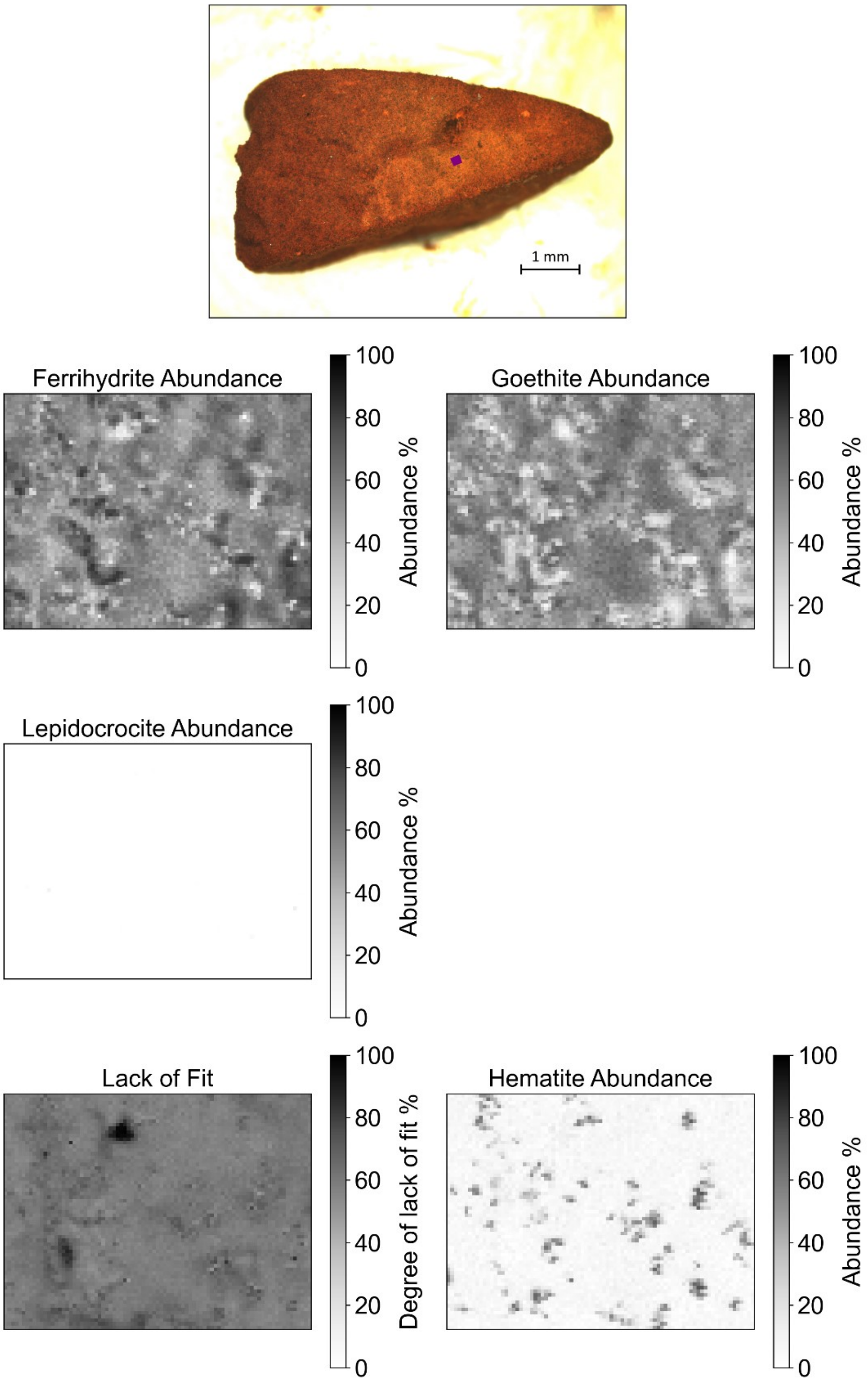

Figure S46: Black and white representation of a 1 µm Raman spectral map of a mineral aggregate cross section from soil BD as presented in Figure S43.

10. References

1. A. Grigg, L. K. Thomas Arrigo, K. Schulz, K. A. Rothwell, R. Kaegi and R. Kretzschmar, Supplementary dataset for "Ferrihydrite transformations in flooded paddy soils: Rates, pathways, and product spatial distributions", *Dataset in the ETH Zurich Research Collection*, 2022, DOI: 10.3929/ethz-b-000569468.
2. X. Fang, J. Wang, H. Chen, I. Christl, P. Wang, R. Kretzschmar and F.-J. Zhao, Two-year and multi-site field trials to evaluate soil amendements for controlling cadmium accumulation in rice grain, *Environ. pollut.*, 2021, **289**, 117918.
3. N. V. Y. Scarlett and I. C. Madsen, Quantification of phases with partial or no known crystal structures, *Powder Diffr.*, 2006, **21**, 278–284.
4. L. K. ThomasArrigo, J. Byrne, A. Kappler and R. Kretzschmar, Impact of organic matter on iron(II)-catalyzed mineral transformations in ferrihydrite-organic matter coprecipitates, *Environ. Sci. Technol.*, 2018, **52**, 12316–12326.
5. E. Zepeda-Alarcon, H. Nakotte, A. F. Gualtieri, G. King, K. Page, S. C. Vogel, H.-W. Wang and H.-R. Wenk, Magnetic and nuclear structure of goethite (α-FeOOH): A neutron diffraction study, *J. Appl. Crystallogr.*, 2014, **47**.
6. A. P. Zhukhlistov, Crystal structure of lepidocrocite FeO(OH) from electron diffraction data, *Kristallografiya*, 2001, **46**.
7. L. Levien, C. T. Prewitt and D. J. Weidner, Structure and elastic properties of quartz at pressure, *Am. Mineral.*, 1980, **65**, 920–930.
8. E. N. Maslen, V. A. Streltsov, N. R. Streltsova and N. Ishizawa, Electron density and optical anisotropy in rhombohedral carbonates. III. Synchrotron X-ray studies of $CaCO_3$, $MgCO_3$ and $MnCO_3$, *Acta Cryst.*, 1995, **51**, 929–939.
9. C. E. White, J. L. Provis, D. P. Riley, G. J. Kearley and J. S. J. van Deventer, What is the structure of kaolinite? reconciling theory and experiment, *J. Phys. Chem. B*, 2009, **113**, 6756–6765.
10. M. Aeppli, R. Kaegi, R. Kretzschmar, A. Voegelin, T. B.-. Hofstetter and M. Sander, Electrochemical analysis of changes in iron oxide reducibility during abiotic ferrihydrite transformation into goethite and magnetite, *Environ. Sci. Technol.*, 2019, **53**, 3568–3578.
11. K. Schulz, L. K. ThomasArrigo, R. Kaegi and R. Kretzschmar, Stabilization of ferrihydrite and lepidocrocite by silicate during Fe(II)-catalyzed mineral transformation: Impact on particle morphology and silicate distribution, *Environ. Sci. Technol.*, 2022.
12. C. W. Childs, Ferrihydrite: A review of structure, properties and occurence in relation to soils, *Z. Pflanzenernähr. Bodenk.*, 1992, **155**, 441–448.
13. K. Sodnikar, K. M. Parker, S. R. Stump, L. K. ThomasArrigo and M. Sander, Adsorption of double-stranded ribonucleic acids (dsRNA) to iron (oxyhydr-)oxide surfaces: Comparative analysis of model dsRNA molecules and deoxyribonucleic acids (DNA), *Environ. Sci.: Process. Impacts*, 2021, **23**, 605–620.
14. L. Nasdala, D. C. Smith, R. Kaindl and M. A. Ziemann, in *Spectroscopic methods in mineralogy*, eds. A. Beran and E. Libowitzky, Mineralogical Society of Great Britain and Ireland, London, 2004.
15. M. J. Pelletier, Quantitative analysis using Raman spectrometry, *Appl. Spectrosc.*, 2003, **57**, 20A–42A.
16. F. Legrand, Espace des couleurs RVB et triangle de Maxwell, https://www.f-legrand.fr/scidoc/srcdoc/image/niveaux/couleurs/couleurs-pdf.pdf, (accessed 12 January 2021).
